# Mind your Ps: A probabilistic model to aid the interpretation of molecular epidemiology data

**DOI:** 10.1016/j.ebiom.2022.103989

**Published:** 2022-04-07

**Authors:** Ana Raquel Penedos, Aurora Fernández-García, Mihaela Lazar, Kajal Ralh, David Williams, Kevin E. Brown

**Affiliations:** a Virus Reference Department, United Kingdom Health Security Agency, London NW9 5EQ, United Kingdom; b National Reference Laboratory for Measles and Rubella, Centro Nacional de Microbiología, Instituto de Salud Carlos III, Madrid, Majadahonda, Spain; c CIBER de Epidemiología y Salud Pública (CIBERESP), Madrid, Spain; d Cantacuzino, National Military-Medical Institute for Research and Development, Bucharest, Romania; e Immunisation and Countermeasures, United Kingdom Health Security Agency, London NW9 5EQ, United Kingdom

**Keywords:** Measles, Outbreak, Elimination, Epidemiology, Molecular epidemiology, Clinical virology

## Abstract

**Background:**

Assessing relatedness of pathogen sequences in clinical samples is a core goal in molecular epidemiology. Tools for Bayesian analysis of phylogeny, such as the BEAST software package, have been typically used in the analysis of sequence/time data in public health. However, they are computationally-, time-, and knowledge-intensive, demanding resources that many laboratories do not have available or cannot allocate frequently.

**Methods:**

To evaluate a faster and simpler alternative method to support the routine interpretation of sequence data for epidemiology, we obtained sequences for two regions in the measles virus genome, N-450 and MF-NCR, from patient samples of genotypes B3, D4 and D8 taken between 2011 and 2017 in the UK and Romania. A mathematical model incorporating time, possible shared ancestry and the Poisson distribution describing the number of expected substitutions at a given time point was developed to exclude epidemiological relatedness between pairs of sequences. The model was validated against the commonly used Bayesian phylogenetic method using an independent dataset collected in 2017–19.

**Findings:**

We demonstrate that our model, using time and sequence information to predict whether two samples may be related within a given time frame, minimises the risk of erroneous exclusion of relatedness. An easy-to-use implementation in the form of a guide and spreadsheet is provided for convenient application.

**Interpretation:**

The proposed model only requires a previously calculated substitution rate for the locus and pathogen of interest. It allows for an informed but quick decision on the likelihood of relatedness between two samples within a time frame, without the need for phylogenetic reconstruction, thus facilitating rapid epidemiological interpretation of sequence data.

**Funding:**

This work was funded by the United Kingdom Health Security Agency (UKHSA). The World Health Organization European Regional Office funded Aurora Fernández-García and Mihaela Lazar training visits to UKHSA.

**Research in context**

**Evidence before this study**

The application of molecular epidemiology is fundamental in the fight against many infectious diseases. This is particularly true for measles as evidenced by searching the PubMed archive for “measles” and “molecular epidemiology”. Countries approaching measles elimination are more often resorting to complementary sequencing windows in addition to the N-450, typically used for genotyping, to distinguish transmission chains and facilitate public health decisions. Tools for analysis of sequence and time data such as BEAST are increasingly used by laboratories and epidemiologists worldwide but are knowledge-, time-, and computationally-intensive, making them inaccessible for those working in under-staffed and under-resourced settings, and impractical for routine application. The identification of measles virus importations is made more challenging by the low number of non-N-450 sequences publicly available: approximately 300 sequences are available in GenBank for the MF-NCR, a non-coding region increasingly employed to distinguish transmission chains, and 100 for the whole genome.

**Added value of this study**

We demonstrate the robustness of a novel and easy-to-use application of molecular evolution modelling to an important public health question: can two cases be epidemiologically related? A simple implementation in the form of a guide and spreadsheet is provided to facilitate the use of this model as a tool. In the course of this work, two measles genome regions were sequenced from over 400 measles patient samples: the N-450 genotyping window and the MF-NCR. These samples were collected during measles outbreaks in the UK and Romania and will be helpful in the interpretation of MF-NCR data collected in other countries during this period and provide more information about this alternative sequencing window.

**Implications of all the available evidence**

The model suggested is simpler than the conventional tools for analysis of sequence/time data and applicable to small sets of data. Its simplicity and low-resource requirement make it amenable to routine deployment. The approach is particularly relevant in the context of measles elimination but, upon validation, can be adapted to different pathogens and contexts.

Alt-text: Unlabelled box

## Introduction

Improvements in sequencing technology have meant that laboratories worldwide can obtain pathogen sequences from patient specimens more cheaply and easily. Sequence data are increasingly employed in support of epidemiology studies to characterise outbreaks and chains of transmission, detect events of nosocomial transmission, or identify immunisation gaps.^1^, ^2^, ^3^, ^4^, ^5^, ^6^ Software using Bayesian approaches to estimate phylogenetic time-scaled trees based on both sequence and time data such as BEAST have gained prominence in molecular epidemiology.^3^^,^^7^, ^8^, ^9^

The World Health Organization (WHO) measles elimination programme is one context where molecular epidemiology has come to the fore in studying transmission chains and identifying gaps in immunisation programs.^10^ Measles virus (MeV), the causative agent of measles, is the most contagious human pathogen currently known with a basic reproductive ratio (R_0_) placed between 12 and 18, posing major challenges for a pure classical epidemiology approach (not employing sequence data) to outbreak control.^11^

**Figure 1:**
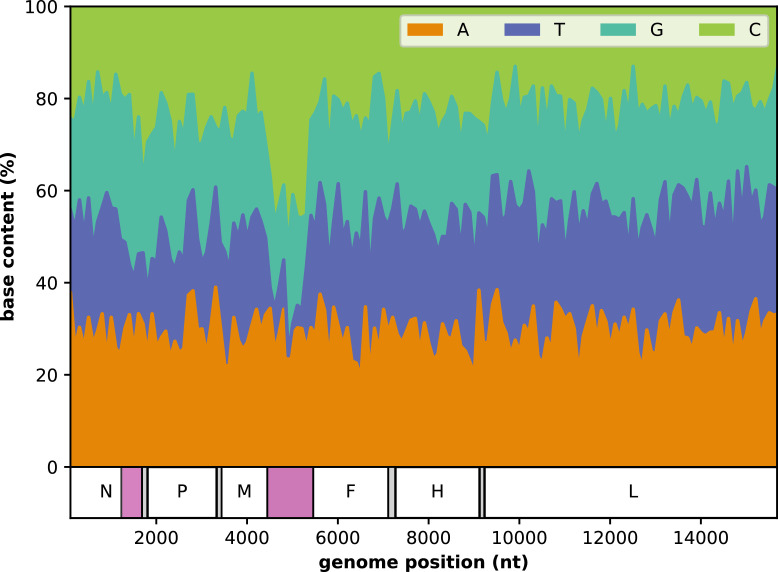
Measles virus (MeV) genome. 15,894 nucleotides (nt) long (15,900 nt in some genotype D4 specimens) which encode for: nucleoprotein (N), phosphoprotein (P), matrix protein (M), fusion protein (F), hemagglutinin (H) and large polymerase (L). Two non-structural proteins, C and V, are also encoded by the P gene. The top panel shows base content across the genome and the bottom panel the genome regions encoding each gene. The N gene region used for genotyping, N-450, and the non-coding region (NCR) between the M and F genes, MF-NCR, are highlighted in pink. The base content is calculated by averaging the fraction of A, T, G and C over each non-overlapping 100 nucleotide window. B3 and D8 sequences used for this plot can be found in Supplement S1 applying the following filters: any except those samples with “GenBank” in the “Sample type” column and a “yes” in the “From WGS” column.

The MeV belongs to the *Paramyxoviridae* family, *Morbillivirus* genus. Its negative-sense single-stranded RNA genome is 15,894 nucleotides (nt) long (15,900 nt in some D4 strains) and encodes six structural proteins (nucleoprotein, N; phosphoprotein, P; matrix, M; fusion, F; hemagglutinin, H; large polymerase, L) and two non-structural proteins (C and V, encoded on the P gene). The non-coding region (NCR) between the M and F genes' transcribed regions (MF-NCR) is 1012 nt long (1018 nt in some D4 strains) and is one of the most variable regions of the MeV genome (Figure 1).^12^^,^^13^

MeV specimens can be divided into different genotypes based on sequence similarity. In 1998, the WHO recommended that designation of new measles genotypes should be based on the sequence of the complete H gene and the portion of the genome encoding the 450 amino-acids of the carboxyl terminus of the nucleoprotein (N-450).^14^ Routine genotyping of the virus relies on N-450 sequencing. WHO laboratories are advised to genotype 80% of transmission chains and sporadic cases for efficient disease surveillance and outbreak control and to submit the sequences collected to the Measles Nucleotide Surveillance (MeaNS) database (https://who-gmrln.org/means2).^10^^,^^15^^,^^16^

In 2012, the WHO member states agreed to a Global Vaccine Action Plan which aims to strengthen vaccination and decrease the burden of diseases such as polio and measles. The implementation of new immunisation programmes and improvements to existing ones led to a steep decline in measles cases worldwide. Of the 8 Genotype Clades (A-H) and 24 MeV genotypes described (A, B1-3, C1-2, D1-11, E, F, G1-3, H1-2), only B3, D4, D8, and H1 were identified worldwide in 2018, with B3 and D8 accounting for 95% of the reported sequences.^15^^,^^17^

Countries across all WHO regions are conducting efforts towards the elimination of endemic measles. Measles is said to be endemic when at least one transmission chain can be detected in a country or region for over 52 weeks. To verify they have eliminated measles, WHO member states must demonstrate the interruption of endemic transmission for at least three years in the presence of a well-performing surveillance system. Showing that no MeV strain has led to continued transmission for more than 52 weeks is part of the verification of elimination process.^18^^,^^19^

The reduction in genetic diversity in the circulating MeV has led laboratories in countries approaching elimination to investigate additional sequencing windows to support their elimination efforts.^3^^,^^4^^,^^19^ The MF-NCR (Figure 1) has been found to yield phylogenetic resolution comparable to that obtained using the whole MeV genome sequence and is increasingly being used in the study of outbreaks.^3^, ^4^, ^5^^,^^12^

In a public health setting, interpreting sequence data correctly is essential so that chains of transmission and immunisation gaps are identified early, and responses like outbreak control or immunisation campaigns can be put in place. However, integrating epidemiology with advanced bioinformatic analyses, e.g., BEAST,^9^^,^^20^ requires trained staff, good computing resources, and time to prepare the data, select models and analyse the results. These resources may not be available to public health laboratories globally or are impractical to deploy on a regular basis. Here, we propose a method by which laboratories can routinely and with minimal effort assess whether two samples, with a known date and sequence, can be related within a given time frame, such as the time to a putative ancestor of the two sequences. We validate this approach against a Bayesian analysis (BEAST) of MeV sequences as a proof of concept, but the methodology can, following appropriate validation, be applied to any pathogen for which good estimates of the substitution rate are available.

## Methods

### Measles virus specimens and isolates

The UK samples used in this study were received between 2011 and 2019 by the Immunisation and Diagnosis Unit at the United Kingdom Health Security Agency (UKHSA) in the context of its role in routine diagnosis and surveillance of measles. They include samples from the 2012–13 outbreak in England and Wales of which 47 whole genome sequences excluding termini (WGS-t) have been published previously (Supplement S1).^3^ The 33 samples from the 2016–17 measles outbreak in Romania were lung biopsies from fatal measles cases (*n* = 8) or throat swabs from severe cases (*n* = 25).^2^ They were processed and extracted at UKHSA as described before.^3^

Of the 500 samples sequenced, the majority were oral fluids (*n* = 232) or throat swabs (*n* = 160). Most samples sequenced belong to measles genotypes D8 (*n* = 254) or B3 (*n* = 236). N-450 and MF-NCR sequences for wild-type strains with a WGS and a WHO name deposited in GenBank until March 2020 (*n* = 83) were included in the analyses to widen the range of sequences and sample times (Supplement S1).

### RNA extraction, RT-PCR and sequencing

Samples for which the N-450 sequence was available and with real-time RT-PCR Ct values less than 30 were selected for MF-NCR sequencing. RNA was extracted using the QIAamp Viral RNA Mini kit (Qiagen®, 52904) as described previously, the ZR Viral RNA kit^TM^ (Zymo Research, R1035), or the NucliSENS® easyMAG® platform.^3^ The RT-PCR amplification was carried out as described previously (Supplement S2).^3^ PCR products were purified with Agencourt AMPure XP PCR purification kit (Beckman Coulter®, A63880), following the kit protocol. The PCR primers were also used for Sanger sequencing following dilution in nuclease-free water (L6F and L7R) or betaine (L6R and L7F) (Supplement S2). All UK and Romania sequences included in this study were deposited in GenBank (the accession numbers are listed in Supplement S1). The N-450 region consists of the last 450 coding nucleotides of the N gene excluding the stop codon, while the MF-NCR consists of the nucleotides between the stop codon of the M gene and the start codon of the F gene (1012 nt, or 1018 nt for some D4 strains).

### Phylogenetic analyses

Phylogenetic analyses were conducted only for genotypes B3, D8 and D4. When necessary, sequences were aligned using Mega 7’s ClustalW alignment with default settings.^21^^,^^22^ IQ-TREE v1.6.10 was used to generate maximum likelihood (ML) trees for the N-450 and MF-NCR sequences individually, and for the concatenated sequences of both regions (using IQ-TREE's partition model). IQ-TREE's model finder was used to identify the nucleotide substitution models best fitted for each dataset and region.^23^, ^24^, ^25^

### Phylodynamic analyses

Time-scaled phylogenetic trees and substitution rates were obtained using BEAST v1.10.4 and the BEAGLE v3.2.0 library was used to accelerate computation.^20^^,^^26^ Prior to a BEAST run, the ML phylogenetic trees, obtained as described above, and sample dates were analysed in TempEst v1.5.1 to verify that a temporal signal is present in the dataset and to remove outliers.^27^ XML files were prepared in BEAUti v1.10.4. Diverse combinations of substitution, clock, and population models were tested. The parameters best explaining the data while still yielding convergent phylogenies and substitution rates were selected for each dataset (Supplement S3.1).

**Figure 2:**
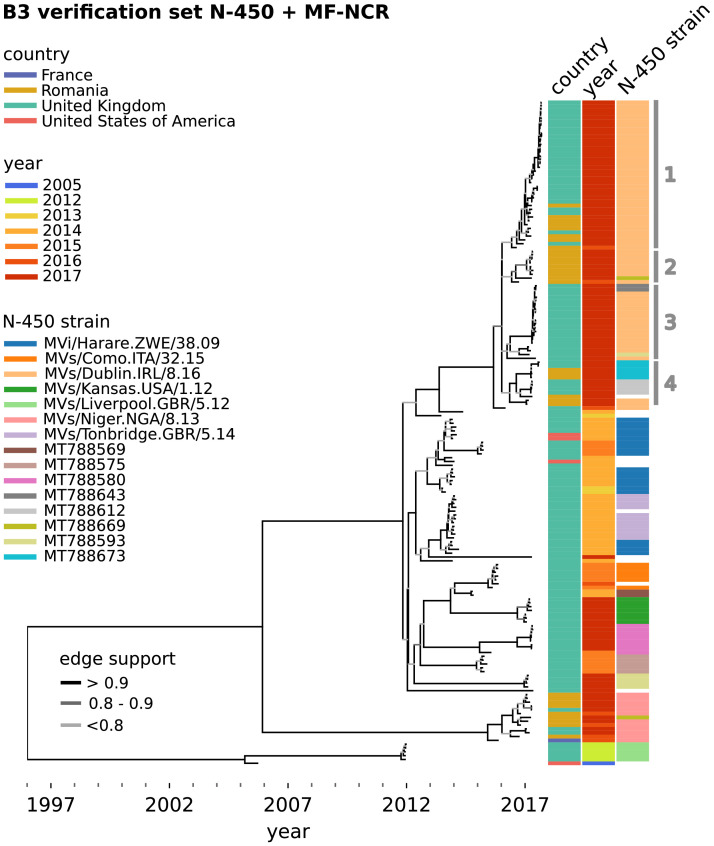
BEAST maximum credibility time-scaled phylogenetic tree for MeV genotype B3 samples in the verification set, used to obtain the substitution rates in Table 1. The N-450 and MF-NCR sequences were concatenated for the analysis. Tree tip labels are colour-coded according to country, sample year and the N-450 sequence (blank if no known matching named strain, or if no more than one sequence is found across datasets). The numbered clades are discussed in the manuscript's text. Named strains are a WHO convention to identify widely circulating MeV strains with identical N-450 sequences.^18^ Samples with non-named N-450 sequences are labelled with the GenBank accession number for one of the sequences. Edge support values are given by the BEAST tree posterior values. N-450 named strain and DSID for all samples are included in Supplement S1.

**Figure 3:**
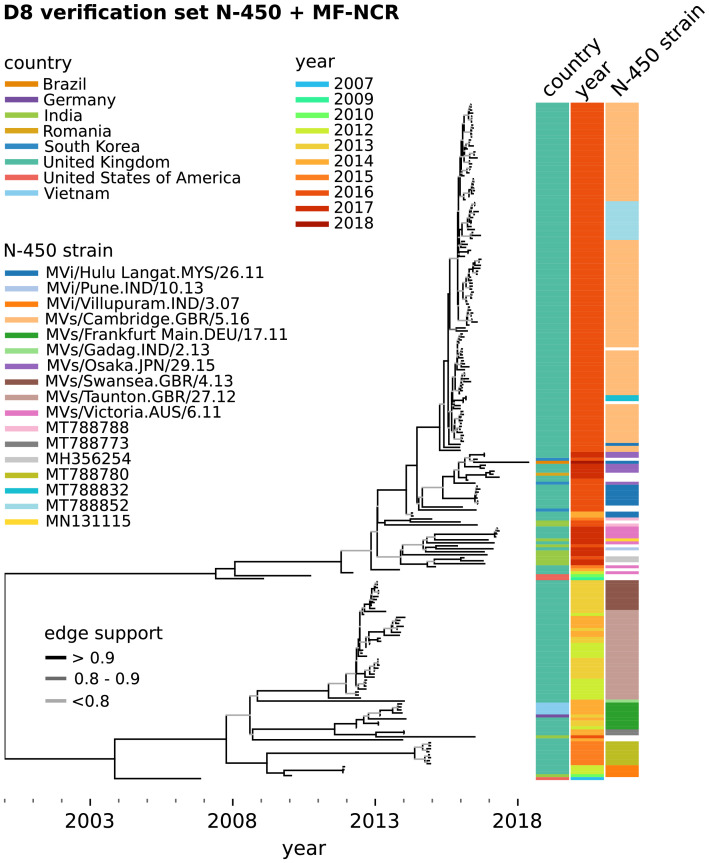
BEAST maximum credibility time-scaled phylogenetic tree for MeV genotype D8 samples in the verification set used to obtain the substitution rates in Table 1 (refer to Figure 2’s caption for details).

Time trees were obtained for the concatenated N-450 and MF-NCR sequences (Figure 2, Figure 3) using BEAST partitions. The clock and substitution models were unlinked between the partitions so that they could best reflect the genome regions’ characteristics and yield a substitution rate for each partition (Tables 1, S6.1, Figures S6.5 and S6.6). A strict clock model was employed for both regions. The same general time reversible (GTR) substitution model with ten gamma heterogeneity categories was employed in both partitions, except that the first and second codon positions were partitioned from the third ((1 + 2), 3) only for N-450, given that the MF-NCR is non-coding. A coalescent Bayesian Skygrid model was used to account for variations in population size.^28^^,^^29^ Four parallel BEAST runs were carried out for each set of conditions to minimise the likelihood of convergence on a local maximum. The results from the four runs were processed into single log and tree files using LogCombiner and a consensus maximum credibility tree was produced by TreeAnnotator using the median to determine the node heights. LogCombiner and TreeAnnotator are both part of the BEAST v1.10.4 package.^20^ The consensus BEAST-inferred phylogenetic trees produced were plotted using Python 3.6 DendroPy v4.4.0 and Matplotlib v3.1.3.^30^^,^^31^ BEAST nexus consensus trees and logs and BEAUti xml files can be found in github.com/phe-bioinformatics/mind-your-ps-2021-manuscript-code.

**Table 1:** Substitution rates in substitutions / (site.year) obtained from the BEAST analyses for the B3 and D8 verification datasets. These are the rates used in the model's verification and validation. The intervals containing the 95% highest posterior intervals for the rate estimates based on the BEAST posterior distributions and the number of sites in the multiple sequence alignments used for the analyses are also listed.

Genotype	Region	Substitution Rate	95% Highest Posterior Density		Number of Sites
			lower	upper	
		subs/(site.year)			
B3	N-450	1·15 × 10^−3^	7·62 × 10^−4^	1·60 × 10^−3^	450
	MF-NCR	1·94 × 10^−3^	1·51 × 10^−3^	2·39 × 10^−3^	1012
D8	N-450	9·23 × 10^−4^	6·53 × 10^−4^	1·20 × 10^−3^	450
	MF-NCR	2·39 × 10^−3^	1·95 × 10^−3^	2·82 × 10^−3^	1012

### Modelling expected substitutions

The number of observed substitutions between two samples was calculated from the number of characters that differ between the sequences in a multiple-sequence alignment (MSA). Ambiguous bases are not counted as differences, unless they do not include any of the bases encoded by the character in the other sequence. For example, R, which encodes A or G, is considered a match to any character that encodes one of these two bases, but not to C, T or Y (C or T). In the case of D4 genotype MF-NCR sequences of different length, each nucleotide insertion or deletion will be counted as a difference (Supplement S4). To obtain the number of substitutions per time, the substitution rates were multiplied by the length of the corresponding MSA: 450 nt for all N-450 datasets, 1012 nt for B3 and D8 MF-NCR sequences and 1019 nt for the D4 MF-NCR dataset (seven insertion and one deletion sites).

Given two samples with *∆t* time between them and a date for a putative common ancestor, pCA (*t_pCA_*), the maximum cumulative evolution time (*∆t_CE_*) that the samples had to evolve since the pCA is calculated as Δ*t_CE_* = 2 Δ*t_pCA_* – Δt, where Δ*t_pCA_* is the interval of time between the most recent sample and the pCA. The number of expected substitutions *λ* at Δ*t_CE_* for a Poisson distribution with *λ* = substitution rate. Δ*t_CE_*. sites, as well as the limits for the interval containing 95% of the same distribution, were calculated using SciPy's stats module.^30^ The explanation of the application of the method and the script used for the calculation of expected substitutions are included in Supplements S4 and S5 and in github.com/phe-bioinformatics/mind-your-ps-2021-manuscript-code, respectively.

### Model verification and validation

Verification of the model was carried out against the BEAST-inferred time-scaled phylogenies for the combined genomic regions of the UK and Romania B3, D4 and D8 datasets obtained until November 2017 (*n* = 427, 43 of which from GenBank). The Bayesian-inferred time to the most recent common ancestor (MRCA) of two samples (*t_bMRCA_*) was calculated from the consensus time tree using DendroPy.^31^ The model and BEAST predictions were made for a set of arbitrary pCAs placed 2 to 52 weeks prior to the most recent sample in each pair using the Python 3.6 SciPy v1.4.1 library.^30^ Each sample pair was then classified as true negative, true positive, false negative and false positive depending on how the model and BEAST predictions compared. The positive predictive value (PPV) was calculated at each Δ*t_CE_* and plotted with the Seaborn v0.10.0 library.^32^

To validate the model with samples independent from those for which the BEAST substitution rates were inferred, UK B3 and D8 sequences obtained between November 2017 and June 2019 (*n* = 156, 40 of which from GenBank) were analysed with BEAST and each sample pair was tested against the rates obtained for the earlier datasets. For these datasets, the substitution model yielding better tree likelihood was the GTR model with ten gamma categories of site heterogeneity. The constant coalescent model was used to estimate population size because the more complex Skygrid model did not converge after four independent chains of 10,000,000 states each, consistent with insufficient signal in the data of changes in effective population size.

### Role of the funding source

This work was funded by the UKHSA, which was responsible for all aspects of the study and the manuscript. The WHO European Regional Office funded the training visits of Aurora Fernández-García and Mihaela Lazar to the UKHSA during which some of the sequences used in the study were acquired and was not involved with any aspects of the work.

## Results

### The MF-NCR locus allows for improved distinction of non-endemic measles over the N-450

The MF-NCR sequence was obtained for samples collected in the UK between 2011 and 2019 and in Romania between 2016 and 2017.^2^^,^^3^ The MF-NCR sequence was successfully obtained for 70% of the samples for which a N-450 sequence was available. The sequencing of the MF-NCR is likely hindered by the presence of more complex secondary structures, homopolymeric regions, lower molarity (only present in the viral genomic RNA) and higher G content in this region than in the remainder of the genome (63 vs. 47%; Figure 1). A longer denaturation step and the use of betaine in the sequencing mix improve outcomes (Figure S6.1).^33^

BEAST analyses were conducted to estimate substitution rates and phylogeny for each genotype and region. MeV N-450 and MF-NCR sequences derived from available GenBank genome sequences were added to the datasets to increase the period covered and the diversity of sequences included in the analyses (Supplement S1). Tree topologies were linked for the N-450 and MF-NCR so that they better represent the real relationships between samples (Figures 2 and 3).

We find that the substitution rate for the MF-NCR genomic region is consistently higher than that for the N-450 region in the B3, D4 and D8 genotype sequences analysed (Table 1), which is in agreement with other studies.^3^, ^4^, ^5^ The mean substitution rates obtained for the D8 N-450 and MF-NCR are 50 and 40% lower than those obtained in the context of the UK measles outbreak of 2012-13.^3^ This can be explained by over-sampling during outbreaks and the wider time frame covered by the present datasets (Figures 2 and 3).^1^^,^^34^ Additionally, the inclusion of 8 samples from fatal cases and 25 from severe cases, although constituting a small fraction of the samples analysed, may affect the substitution rate estimates.

The trees obtained (Figures 2 and 3) illustrate the added resolution conferred by sequencing the MF-NCR in addition to the N-450. Examples of this are the phylogenetic cluster containing samples sharing the MVs/Dublin.GBR/8.16[B3] (Dublin 2016) N-450 sequence (Figure 2, phylogenetic clades 1–4) and that containing specimens with N-450 identical to MVs/Cambridge.GBR/5.16[D8] (Cambridge 2016) and the N-450 sequence with GenBank accession MT788852 (Figure 3).

The large cluster containing the Dublin 2016 N-450 sequences can be sub-divided into four clades (1–4) which share a common ancestor with a case in the UK on week 16 of 2016 (Figure 2). Clade 2 is composed solely of samples collected in Romania, where a large outbreak was ongoing in 2016–17.^2^ The earliest samples in clades 1 and 4 are also Romanian. Only clade 3 is composed solely of UK samples. This suggests that the large outbreak of Dublin 2016 and in the UK in 2016–17 likely resulted from multiple importations of MeV circulating in Europe at the time rather than from endemic transmission.

In contrast, without further epidemiological data, the large cluster containing the Cambridge 2016 and MT788852 N-450 sequences (Figure 3) appears to be the result of sustained transmission in the UK between weeks 10 and 46 of 2016. The MT788852 N-450 sequence differs from that of Cambridge by a single nucleotide, suggesting that a substitution event occurred early in the outbreak.

### Relatedness between sample pairs can be excluded without phylogenetic reconstruction

Genome nucleotide substitutions during evolution can be modelled as a succession of events. Given that high substitution rates are selected against due to their impact on fitness, the number of substitutions observed on a given genome each infection cycle is low relative to the number of sites. In these circumstances one substitution typically occurs independently of a previous one.

This type of process can adequately be described by a Poisson distribution where the Poisson shape (or rate) parameter *λ* can be calculated at a time *t* by multiplying the substitution rate, *µ* (in substitutions per unit of time and number of sites), by *t* and the length of the region in question.^7^^,^^35^ The *λ* then represents the expected number of substitutions after time *t* in a genomic region when compared to the initial sequence it evolved from. The interval centred on the mean and containing fraction α (e.g. 95%) of the Poisson distribution can be used to predict the upper and lower number of substitutions expected at that time.

In a public health setting, laboratories and epidemiologists do not have access to sequence data for every case and hence cannot assume that an earlier sample is a direct ancestor of one collected later. This is particularly true for measles, given that it is an acute and, for the majority of patients, mild illness, often transmitted before symptom onset, and highly contagious. In most cases, two samples will have derived from a common ancestor that may or may not be known, and hence will have diverged over the time that separates both samples from that ancestor.

**Figure 4:**
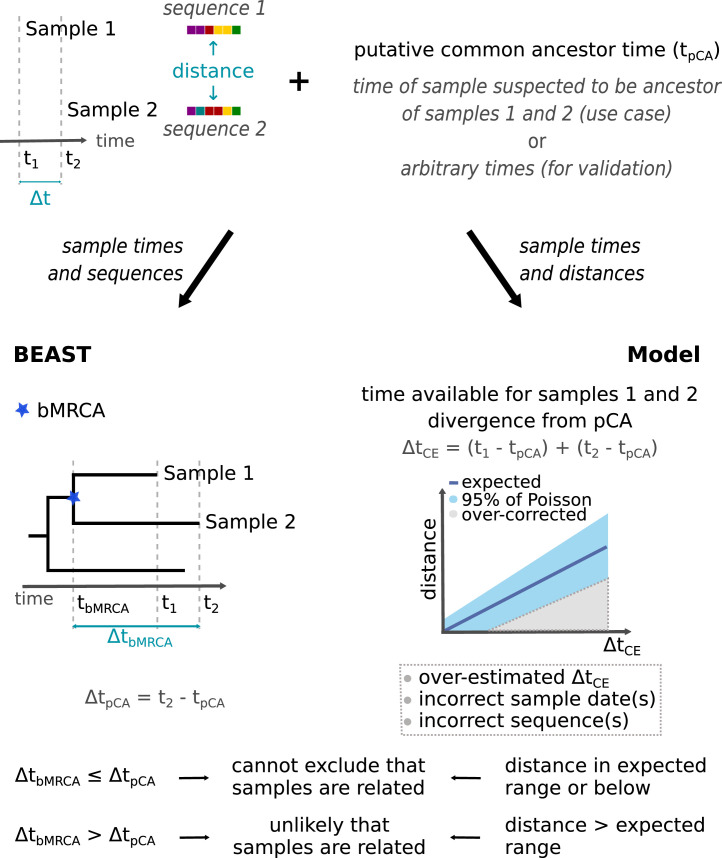
Evaluating the null hypothesis that two samples have derived from a putative common ancestor (pCA) in a given time frame using BEAST or the proposed, non-phylogenetic, probabilistic approach. The sequences and sampling times of all sample pairs are used to estimate a time-scaled phylogeny using BEAST. The distance between the sequences in each pair and the time the sequence pair had to diverge from a putative common ancestor are used to make relatedness predictions based on a Poisson distribution. Details of the model and its validation can be found in Supplement S3. A protocol and examples of application can be found in Supplements S4 and S5.

**Figure 5:**
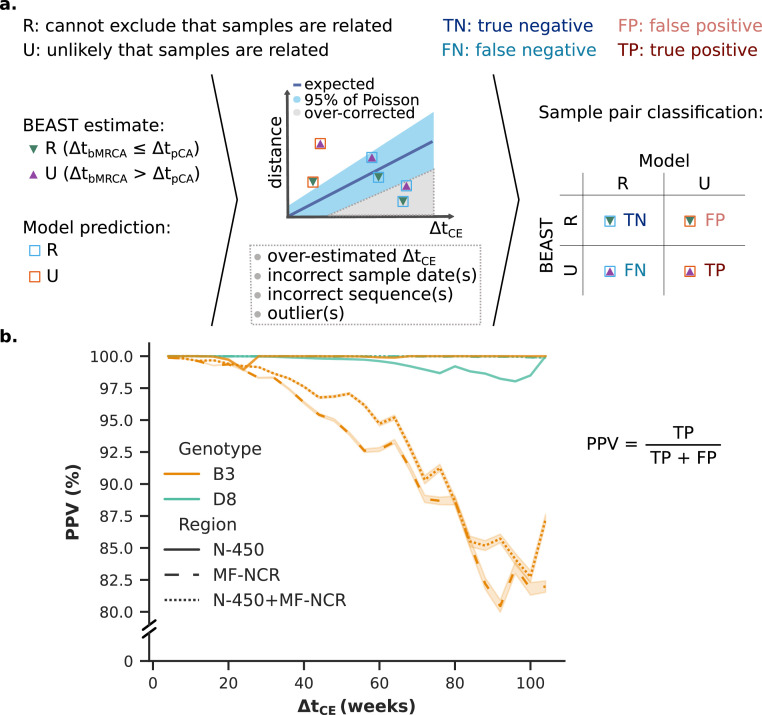
(a) Classification of sample pairs according to the model predictions as compared to the BEAST estimates. (b) Positive predictive value (PPV) for the validation set containing UK and GenBank N-450 and MF-NCR sequences independent of those used to calculate the substitution rates employed in the model prediction. The shaded areas around each line in the plot represent the 95% CI based on the results for each of the 3604 trees in the BEAST posterior for each dataset. Please refer to Supplement S3 for additional detail.

One of the most commonly asked questions in public health virology is “Could case B be associated with the same cluster of epidemiologically-linked cases that led to an earlier case A?”. To answer this question, a method to resolve membership of a cluster for a group of potentially related cases is needed. The duration of the cluster, and the timing of the pair of cases in question, determines the minimum and maximum number of substitutions that are likely to have accumulated between the sequence pair. By combining a previously inferred substitution rate, the onset date of the two samples and of the earliest known case in a cluster, we can derive a Poisson distribution shape parameter. We can then assess whether the two samples can be epidemiologically related within the apparent time frame of that outbreak comparing the number of differences between their respective sequences with the range predicted by the Poisson distribution with the derived shape parameter (Figures 4, 5 and Supplement S3).

Often, it is when cases are related that stronger public health responses (e.g. outbreak control) must be triggered. To minimise the chance that two related samples are incorrectly classified as unrelated, and hence the risk that public health measures would not be implemented when required, the conservative (null) hypothesis is that two samples are related, and thus part of the same epidemiological cluster. Only if there is sufficient evidence, i.e., an excess of substitutions, do we exclude that assumption. We can plot a sample pair against an expected substitution range by determining the number of differences (distance) between the sequences and calculating the cumulative time that they had to evolve from a case presumed to be a potential ancestor of both, putative common ancestor (cumulative evolution time). The location of the point in the plot allows us to assess whether the pair's distance is within the expected range of substitutions for the cluster's time frame and a chosen probability interval (Supplement S3). Laboratories and epidemiologists can easily apply this method without the need for phylogenetic analyses (Supplements S4 and S5).

Application of this model requires two inputs. First, the collection or onset dates and sequences for the two samples for which the epidemiogical relatedness is being tested. Second, the duration of the cluster of epidemiologically linked cases associated with one of the samples, indicated by the time of the earliest known case. The precise method of cluster delineation, typically integrating classical epidemiological investigations with molecular evidence may vary. A cluster may be an outbreak with strong evidence of fully sampled chains of transmission, or an entire period of endemicity dated from the earliest known case following an importation. In the latter scenario, ruling out relatedness would be indicative of importations independent to that under consideration. However, any cluster definition considered epidemiologically sound by the investigator, would be equally valid.

### High PPV in the prediction of unrelated sample pairs

BEAST is held as the gold standard for sequence/time data analysis. To verify our model, we calculate the distance and cumulative evolution time for every pair in each of the datasets as described above and obtain the model's prediction (unable to exclude relatedness or unlikely related) for a range of putative common ancestor times (Supplement S3.3). We compare these predictions to the BEAST t_bMRCA_ estimates (Figure 5). This is done in an epidemiology-agnostic manner, with all pairwise distances among samples being considered with different putative common ancestor times.

The results for each pair can then be classified as true positive (unrelated samples classified as unrelated), true negative (related samples classified as related), false positive (related samples classified as unrelated), and false negative (unrelated samples classified as related) (Figure 5a). We demonstrate that the proposed method predicts that two sequences are unlikely to be related within a given time frame with high positive predictive value (PPV) (Figure 5b). For the validation datasets comprising D4 and D8 N-450 and MF-NCR sequences, and B3 N-450 sequences likely unrelated sample pairs are predicted with over 97.5% PPV. For the B3 MF-NCR validation dataset, the PPV falls below 90% after approximately 70 weeks of cumulative evolution time. This can be explained by a deviation of the substitution rate obtained for the B3 MF-NCR verification dataset (1·94 × 10^−3^, 1·51 × 10^−3^-2·39 × 10^−3^ 95% HPD substitutions/(site x year)) from that of the validation dataset (4·44 × 10^−3^, 2·44 × 10^−3^-6·40 × 10^−3^ 95% HPD substitutions/(site x year)) (Table S6.1, Figures S6.5 and S6.6).

## Discussion

BEAST is typically used in the context of molecular epidemiology to determine the time of divergence between samples. Complemented with epidemiological data, it is a powerful tool in outbreak characterisation, identification of sources of clusters of illness, and supporting the interpretation of contact information. However, the use of BEAST and other Bayesian phylogenetics tools requires the collection of representative sequences, good understanding of the tools and of result interpretation, computational capacity, and time. This type of analyses is impractical on a routine basis. Standard phylogenetic analysis is more feasible, and software such as IQ-TREE facilitates the choice of adequate substitution models to the dataset, limiting issues with selection of inappropriate models or model over-fitting associated with phylogenetic analyses. However, the interpretation of sample clustering and divergence in phylogenetic trees is more challenging.

Here, we suggest a method which can be used by public health laboratories and epidemiologists to support their interpretation of incomplete sequence datasets on an everyday basis, without the need for phylogenetic analyses. In the context of countries close to measles elimination, the recommended approach is to consider transmission as endemic if no evidence of importations of the virus can be found, which is reflected in our null hypothesis. In the estimation of the cumulative time two samples may have had to evolve since a recorded ancestor, we seek to minimise the false positive rate, i.e., the proportion of potentially related sample pairs for which relatedness is rejected. We find that we can exclude that a sample pair is related in a given time frame with over 90% PPV for cumulative evolution times up to 70 weeks (Figure 5b).

Bayesian analyses should be used when retrospectively analysing data and seeking more definite conclusions because the model suggested here is a simplification and relies on a previous reliable estimate for the substitution rate. The Hamming distance matrix employed to score sequence distances is a simple count of differing bases and does not take into account the likelihood of different mutation events or other substitution model parameters. This simplification facilitates rapid assessment without the need for further analyses or tools. Despite it, the model performed well. Over long periods of time, the Hamming distance stops being a good description of the evolutionary process, given that reversion of sites to prior bases or more than one substitution at a site would lead to the under-detection of substitution events. However, this would increase the rate of false negatives rather than that of false positives, simply reducing the method's usefulness in excluding sample relatedness, and thus still fulfil the overall goal of the approach.

For verification of endemic measles elimination, the WHO encourages countries to rely on epidemiology data. These data should be complemented with N-4-50 sequence data to identify chains of transmission and epidemiological clusters of cases. The use case for the method validated in this study is in support of the analyses of combined epidemiological and sequence data, not in place of. When insufficient epidemiological data are available to distinguish clusters of cases or interpret phylogenetic analyses with confidence, the application of the suggested approach can provide insight into the data. This model was validated in an epidemiology- and phylogenetic cluster-agnostic manner. Doing so minimises the impact of incomplete and potentially incorrect epidemiological data and mimics the application scenario proposed for the method more closely. Occasionally, public health authorities are able to integrate full phylodynamic analyses with detailed epidemiological investigations of consecutive outbreak clusters. The data from those instances would be ideal to further validate this model.

The approach suggested here is straight forward and flexible. The substitution rate against which likelihood of relatedness is estimated can be selected according to the size and type of the dataset and updated when more sequences are available. This model can be applied to individual pairs of samples or to phylogenetic tree tips and clusters to facilitate the interpretation of the results, to either visualise where sample pairs or clusters fall in the range of expected substitutions or to calculate a likelihood that their distance would be observed in a time frame if the samples were related. This can be done without additional bioinformatics analyses, following a simple procedure that requires no more than pre-obtained expected substitutions plots (Supplement S4) or a spreadsheet (Supplement S5).

## Contributors

ARP: experiment design, data acquisition, analyses and interpretation, figures and manuscript preparation; AFG: experiment design, data acquisition and critical review of the manuscript; ML: data acquisition and critical review of the manuscript; KL: data acquisition and critical review of the manuscript; DW: data analyses and interpretation and critical review of the manuscript; KEB: experiment design, data interpretation and critical review of manuscript.

### Data sharing statement

Available from the corresponding author upon reasonable request.

## Declaration of interests

No conflict of interests declared.

## Appendix Supplementary materials

Image, application 1

Image, application 2

Image, application 3

Image, application 4

Image, application 5

Image, application 6

Image, application 7
