# Supplementary material for: Mind your Ps: A probabilistic model to aid the interpretation of molecular epidemiology data
Source: eBioMedicine. 2022 Apr 7;79:103989. doi: 10.1016/j.ebiom.2022.103989 (PMC9006250; doi:10.1016/j.ebiom.2022.103989)
Supplement: Supplementary file 2 [file mmc2.docx]

S2: RT-PCR primers, mixes and conditions

# Primers

|  | **Forward Primer** | | **Reverse Primer** | |
| --- | --- | --- | --- | --- |
| **Amplicon** | **Name** | **Sequence (5' > 3')** | **Name** | **Sequence (5' > 3')** |
| L6 | MeV-L6-2F | CGGGAACTTCAGGAGAAAG | MeV-L6-3R^3^ | CCCCCCGTCTTGGAYTGTCG |
| L7^1^ | MeV-L7nF | AGACCACCAACCGCATCCC | MeV-L7nR | GGCTCGCTCTCAGATTGTCG |
| L7n^2^ | MeV-L7-1F^3^ | CGCACAAGCGACCGAGGTG | MeV-L7-3R | CTAGGGCCGCACCTGCCAG |

^1^ RT-PCR round

^2^ second PCR round

^3^ Primers L6-2F, L6-3R, L7-1F and L7-3R are used for Sanger sequencing. Primers anchoring inside the MF-NCR (L6-3R and L7-1F) are diluted in betaine for sequencing (Fig S1).

# 1^st^ round (RT-PCR) – L6 & L7

Kit: OneStep RT-PCR (Qiagen®, 210212)

## Mix

| **Reagent** | **Volume / rxn (µl)** |
| --- | --- |
| Nuclease-free water | 20 |
| 5x buffer | 10 |
| 5x Q | 10 |
| dNTPs | 2 |
| 30 µM primer mix | 1 |
| Enzyme mix | 2 |
| RNA | 5 |
| *Total volume* | 50 |

## PCR conditions

| **Stage** | **T (ºC)** | **time** |
| --- | --- | --- |
| RT | 50 | 30 minutes |
| Initial denaturation | 95 | 15 minutes |
| 40 cycles of: |  |  |
| - Denaturation | 94 | 80 seconds |
| - Annealing | 55 | 90 seconds |
| - Extension | 72 | 2 minutes |
| Final extension | 72 | 10 minutes |

# 2^nd^ round (PCR) – L7n

Kit: Taq master mix (Qiagen®, 201443)

## Mix

| **Reagent** | **Volume / rxn (µl)** |
| --- | --- |
| Nuclease-free water | 19 |
| 30 µM primer mix | 1 |
| 2x master mix | 25 |
| cDNA | 5 |
| Total volume (µl) | 50 |

## PCR conditions

| **Stage** | **T (ºC)** | **time** |
| --- | --- | --- |
| Initial denaturation | 94 | 3 minutes |
| 40 cycles of: |  |  |
| - Denaturation | 94 | 80 seconds |
| - Annealing | 55 | 90 seconds |
| - Extension | 72 | 2 minutes |
| Final extension | 72 | 10 minutes |
