# Supplementary material for: Mind your Ps: A probabilistic model to aid the interpretation of molecular epidemiology data
Source: eBioMedicine. 2022 Apr 7;79:103989. doi: 10.1016/j.ebiom.2022.103989 (PMC9006250; doi:10.1016/j.ebiom.2022.103989)
Supplement: Supplementary file 3 [file mmc3.docx]

Supplement S3: Bioinformatics methods

[S3.1. BEAST estimates 1](#_Toc88938310)

[S3.2. Model concepts 3](#_Toc88938311)

[S3.3. Validation 4](#_Toc88938312)

# S3.1. BEAST estimates

The approach employed to conduct the BEAST analyses for this study is shown in Fig S3.1.


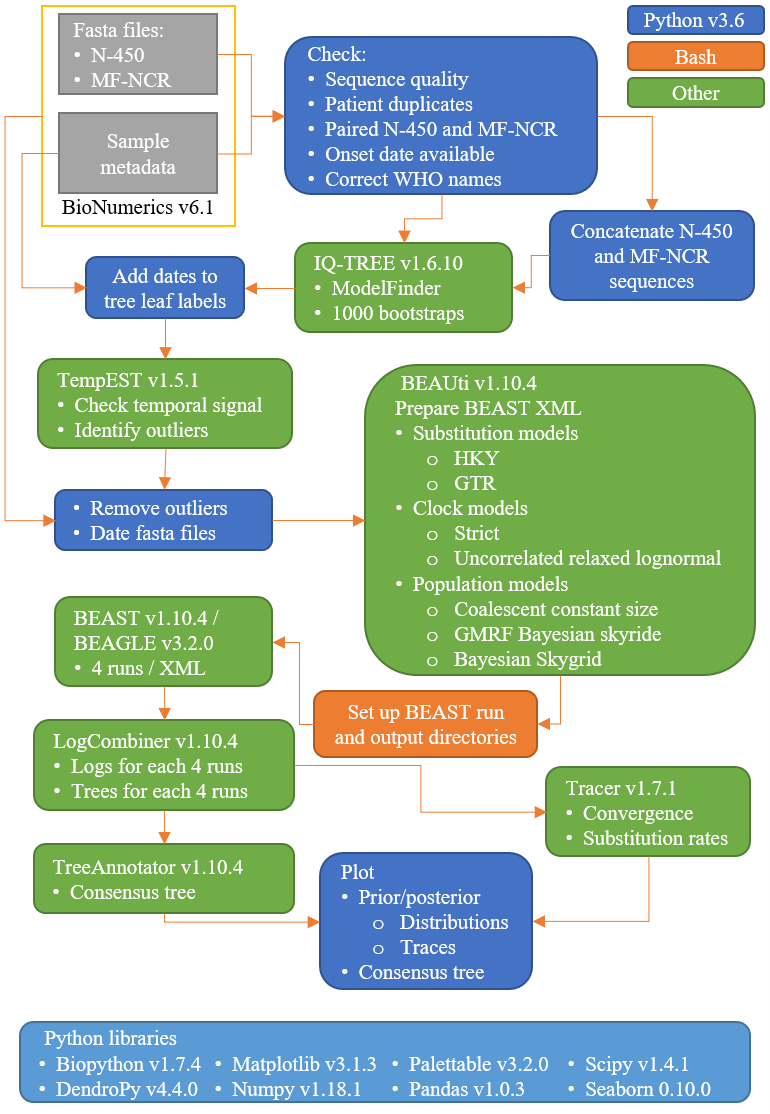


**Fig S3.1:** Procedure for sequence data preparation and analysis using BEAST v1.10.4.

The parameters tested for BEAST analysis are summarised in Table S3.1. All parameter combinations were tested for all datasets.

**Table S3.1:** Combinations of parameters tested in BEAST. Only adjusted parameters are listed. Remaining choices available in BEAUti v1.10.4 were left as default. Model complexity increases from A to I.

|  | ***Run label*** | | | | | | | | |
| --- | --- | --- | --- | --- | --- | --- | --- | --- | --- |
| ***Parameters*** | ***A*** | ***B*** | ***C*** | ***D*** | ***E*** | ***F*** | ***G*** | ***H*** | ***I*** |
| *partitions* | 1 | | | 1 or 2^a^ | | | | 1 | 2^a^ |
| *substitution model* | HKY | | GTR | | | | | | |
| *base frequency* | estimated | | | | | | | | |
| *site heterogeneity* | none | | | γ = 4 | γ = 10 | γ = 4 | γ = 10 | | |
| *codon partitions* | none | | | (1+2),3 for N-450 | | | | | |
| *clock* | strict | uncorrelated relaxed clock/lognormal | strict | | | | | | |
| *prior* | coalescent constant size | | | | | | | Skyride | Skygrid^d^ |
| *model* | random starting tree | | | | | | | | |
| *reconstruct states* | no | | | | | | | | |
| *chain length* | 10,000,000 | | | | | | | | |
| *log every* | 10,000 | | | | | | | | |
| *weights* | default | | | | | See b | default | | |
| *tuning* | default | | | | | | See c | default | |

1. for concatenated N-450 and MF-NCR
2. GTR.rates and frequencies weight reduced from 1 to 0.5; constant population size increased from 3 to 10; local rearrangements of tree increased from 30 to 40; global rearrangements of tree increased from 3 to 5
3. sub-tree slide rearrangement of tree tuning changed from 1 to 0.8 (warmer); constant population size decreased from 0.75 to 0.5 (warmer)
4. 50 parameters; time at last transition point: 10.0

Model complexity increases from A to I. Higher complexity models are more likely to yield results that are more representative of the population. However, complexity must be balanced with the amount of information present in the dataset analysed and smaller/less diverse datasets may lead to non-converging MCMC chains. Convergence was assessed using Tracer v1.7.1.

BEAST analyses were carried out for two independent subsets of the data. For the data collected between 2011 and November 2017 (verification set), a coalescent Bayesian Skygrid model^[[1]](#footnote-1)^ was used to account for variations in population size (Table S3.1, parameter set I). For the data obtained between November 2017 and 2019, the constant coalescent model^[[2]](#footnote-2)^ was selected (parameter set E) given that the Skygrid model run (I) did not converge. Burnin was set at 1 million chains in LogCombiner. The XML used for the chosen parameters are included in the [manuscript’s GitHub repository](https://github.com/phe-bioinformatics/mind-your-ps-2021-manuscript-code).

#
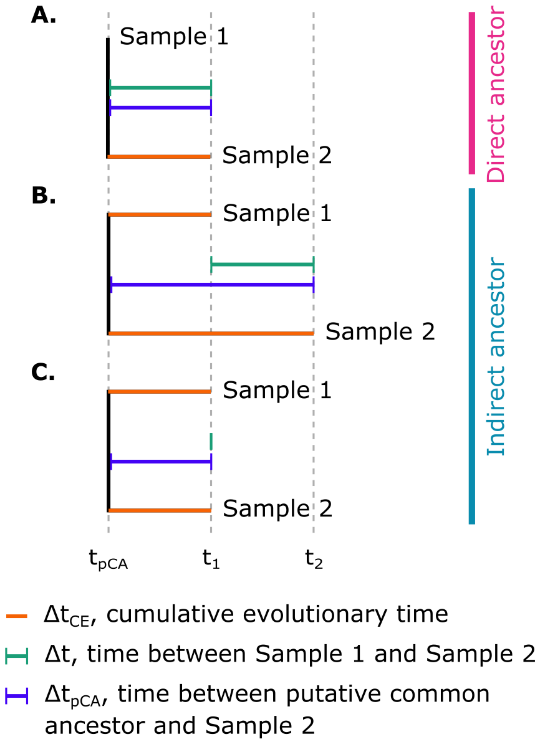
S3.2. Model concepts

**Fig S3.2:** Depiction of the definitions for the time concepts employed in the model formulation and validation.

The time parameters employed in the definition and validation of the proposed model are illustrated in Fig S3.2.

In most measles outbreaks, the direct ancestors of a sample are not known. Hence, sample 1 should only be counted as a direct ancestor of sample 2 if there is strong epidemiological evidence this is the case or if the time between samples 1 and 2 (*Δt*) is sufficiently long that incorrect prediction of the time between sample 2 and the correct direct ancestor would have limited impact in the calculation of the maximum time available for sample 2 to diverge.

To take into account that in most cases it is unknown whether sample 1 is a direct ancestor of sample 2, it is important to consider the maximum time both samples could have had to evolve (*∆t_CE_*; Fig S3.2) from a measles case that could have been an ancestor of both samples 1 and 2 (putative common ancestor, pCA) based on the timeline of cases and epidemiology data. The pCA is likely different from a most recent common ancestor (MRCA), as the latter is often unknown in epidemiology. Underestimating *∆t_CE_* can lead to erroneous exclusion of relatedness (Fig S3.3). In Fig S3.3, the dark blue line represents the expected number of substitutions expected at each time point and the sahded lighter blue area is the range of substitutions expected to include 95% of the distribution.
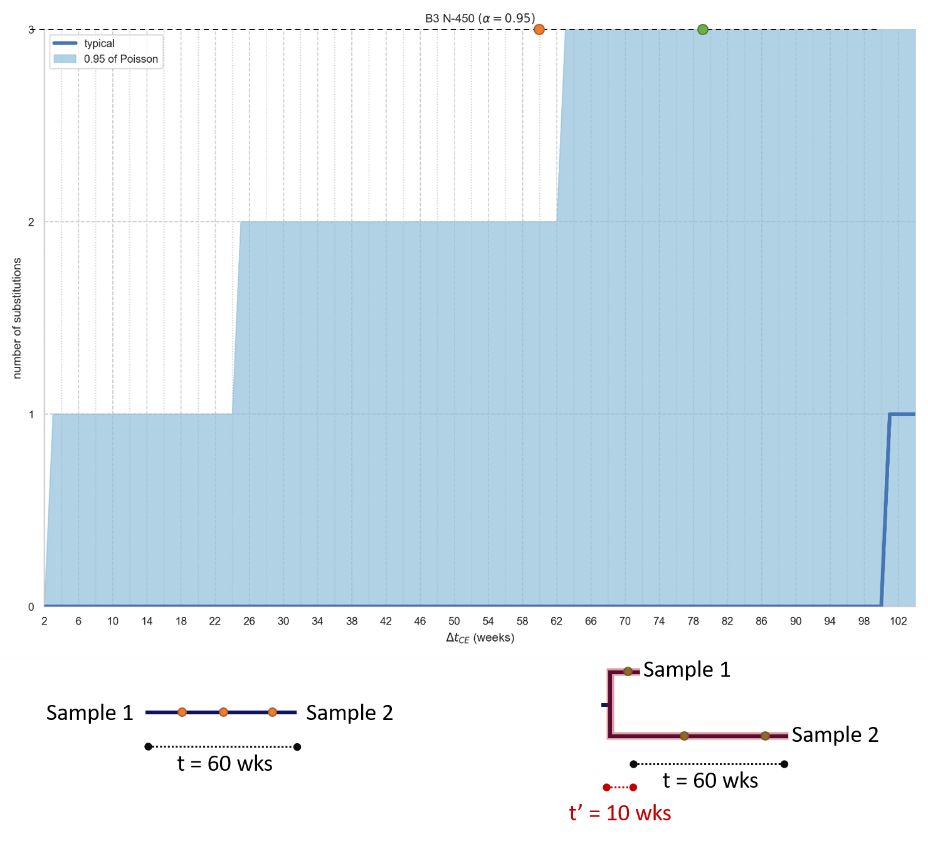


**Fig S3. 3:** Accounting for the cumulative evolution time available for samples to diverge from a common ancestor affects model predictions.

The application of a Poisson distribution to estimate expected substitutions and predict relatedness between samples is described in the manuscript section “Modelling expected substitutions” of the Methods and section “Relatedness between sample pairs can be excluded without phylogenetic reconstruction” of the Results.

Expected substitutions are calculated from a Poisson distribution with rate parameter given by Equation 1, where µ is the substitution rate in substitutions / (site x year), s is the number of sites in the multiple sequence alignment for the genomic region the substitution rate applies to and *∆t_CE_* is the cumulative evolution time (in years) for which the expected substitution range is being calculated.

$\lambda=\mu\cdot s\cdot{\Delta t}_{CE}$ Equation 1

The 95% probability interval for the obtained Poisson gives the lower and upper limits of the number of substitutions expected in a given time frame that would encompass that proportion of the distribution.

The time available for divergence is the time sample 1 had to evolve since the pCA added to the time sample 2 had to evolve since the pCA. This equates to $2{\Delta t}_{pCA}-\Delta t$ as demonstrated in Equation 2. When *t_1_* can be approximated to *t_pCA_*, ${\Delta t}_{CE}=\Delta t$.

$\begin{aligned} {\Delta t}_{\mathrm{CE}}=\left( t_{1}-t_{\mathrm{pCA}} \right)+\left( t_{2}-t_{\mathrm{pCA}} \right) \\ =t_{1}+t_{2}-2{\Delta t}_{\mathrm{pCA}} \\ =t_{1}+t_{2}-2\left( t_{2}-t_{\mathrm{pCA}} \right) \\ =\left( t_{1}-t_{2} \right)+2{\Delta t}_{\mathrm{pCA}} \\ =2{\Delta t}_{\mathrm{pCA}}-\left( t_{2}-t_{1} \right) \\ =2{\Delta t}_{\mathrm{pCA}}-\Delta t \end{aligned}$ Equation 2

# S3.3. Validation

The model predictions are evaluated against the BEAST estimates, given that the latter is seen as the gold standard in measles molecular epidemiology. The method was assessed in two stages: verification and validation. To verify that the concept is applicable, the same sequences were used to obtain a BEAST time-scaled phylogeny and the substitution rate employed in the model predictions. This dataset was composed of B3, D4 and D8 samples sequences collected between 2011 and November 2017 (for more sample details, see Supplement S1). The validation stage used an independent set of sequences obtained from samples collected between December 2017 and November 2019. For this dataset, a BEAST-estimated time-scaled phylogeny was used to validate the model predictions made with the substitution rate obtained for the verification dataset.

The verification and validation datasets were split into genotypes:

- Verification (2011-2017 samples)
  - B3, 174 sequences which can be combined into 15051 sequence pairs
  - D4, 24 sequences, 276 pairs
  - D8, 227 sequences, 25651 pairs (2 outliers excluded from analysis)
- Validation (2017-2019 samples)
  - B3, 90 sequences, 4005 pairs (1 outlier excluded from analysis)
  - D8, 65 sequences, 2080 pairs

Because the use case of this model is for situations where epidemiological data is limited and cannot provide sufficient information for epidemiological cluster distinction, the analysis was conducted without reference to epidemiological clusters for the sequences being analysed. To apply the approach used in this work, an epidemiologist or laboratory worker would collect sequence and sample date data for the samples of interest and assess whether samples 1 and 2 could have derived from a putative common ancestor (pCA) in the time frame (Supplement S4 for protocol and examples). The pCA would be a sample that is hypothesised to be an ancestor of both sample 1 and sample 2. For example, if there was a measles outbreak in city A for a 6-month period and 4 months into that outbreak, a similar sequence is found in a second outbreak in city B, the time of the pCA should be the date of the first case of measles detected in city A. To assess the model predictions independently of epidemiology data which can be incorrect or incomplete, we simulate pCAs occurring every 2 weeks between one measles incubation period (*∆t_pCA_*=2 weeks) and one year (*∆t_pCA_*=52 weeks) before the most recent sample in each pair.

The same steps were repeated for all pairs of samples. The analysis steps are as follows:

1. Calculate expected substitutions for range of cumulative evolution times *∆t_CE_*, i.e. the time available for a pair of samples to diverge from a pCA.
   1. Minimum of the range is 4 weeks given that the pCA must have occurred at least one incubation period before both samples in the pair.
   2. Maximum of the range is 104 weeks (when samples 1 and 2 were collected in the same week and the pCA occurred 52 weeks before, 2 x 52 weeks).
   3. Maximum number of expected substitutions (*d_max_*) is calculated for *∆t_CE_ = 4, …, 104*
2. Model predictions for each sequence pair (the protocol is included in Supplement S4; the Python code used in the analysis is available from the [manuscript’s GitHub repository](https://github.com/phe-bioinformatics/mind-your-ps-2021-manuscript-code)):
   1. Calculate Hamming distance (*d*) between the sequences in the pair using the distance matrix given in Supplement S4.1.
   2. Calculate time between the samples (*∆t*).
   3. For each *∆t_pCA_*:
      1. If the time between the most recent sample and the pCA is smaller than *∆t*, model predictions are not made for the pair (if the earliest sample happened before the pCA, then it cannot be a common ancestor.
      2. If *∆t_pCA_ ≥ ∆t*, calculate the *∆t_CE_* for the sample pair with that pCA. This is given by the formula ${\Delta t}_{CE}={2\Delta t}_{pCA}-\Delta t$.
         1. Compare d with the maximum expected substitutions for the *∆t_CE_*.
            1. If $d>d_{max}$ then the sample pair is predicted as positive (unlikely related in the time frame).
            2. If $d\leq d_{max}$ the pair is predicted as negative (unable to exclude relatedness in the time frame).
3. Obtain the BEAST estimate for each of the 3604 trees in the BEAST posterior.
   1. Get the time of the BEAST-estimated MRCA of samples 1 and 2 (*t_bMRCA_*).
   2. Compare *t_bMRCA_* to *t_pCA_*.
      1. If BEAST estimates a MRCA longer ago than the pCA, $t_{bMRCA}<t_{pCA},$ the pair is evaluated as positive (unlikely related in the time frame) – insufficient time between pCA and the samples to accumulate the substitutions observed.
      2. If BEAST estimates a MRCA more recent than the pCA, $t_{bMRCA}\geq t_{pCA}$, then the sample pair is evaluated as negative (unable to exclude relatedness in the time frame) – there would have been sufficient time for divergence between pCA and the samples.
4. Classify pairs as true negative, true positive, false negative or false positive, depending on the BEAST and model predictions (Fig 5a).
5. Calculate PPV (and other statistics) for each dataset and *∆t_CE_*. The lines in the plots in Figs 5 and S6.8-11 correspond to the mean value of the rate, the shaded areas around the lines indicate the 95% confidence interval based on the 3604 trees x number of pairs for the dataset classified at that time point (Fig S6.7).


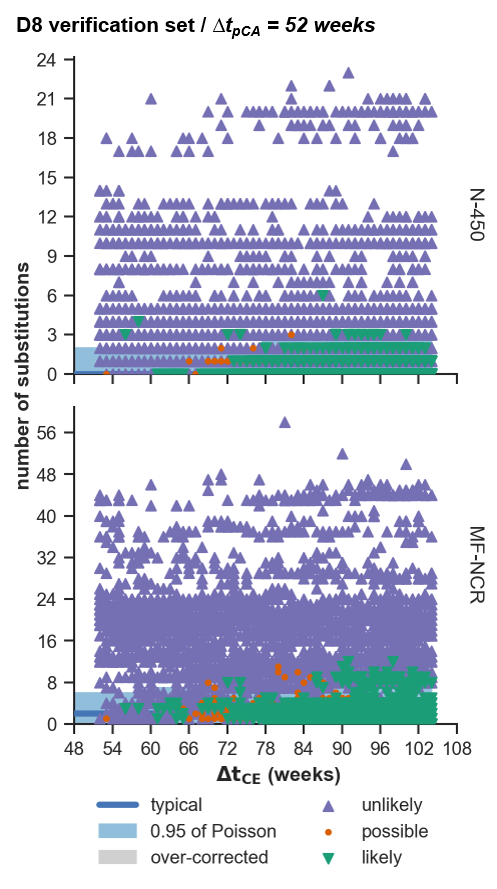


**Fig S3.4**: Illustrating sample pair classification based on BEAST estimates (marker colour and shape) and model prediction (based on location in expected substitution plot). Sample pairs where the BEAST-estimated time of the MRCA is up to 10% higher than the time of the pCA are coloured orange.

1. Gill, M. S., Lemey, P., Faria, N. R., Rambaut, A., Shapiro, B. & Suchard, M. A. 2013. Improving Bayesian population dynamics inference: a coalescent-based model for multiple loci. Mol Biol Evol, 30, 713-24. [↑](#footnote-ref-1)
2. Kingman, J. F. 2000. Origins of the coalescent. 1974-1982. Genetics, 156, 1461-3. [↑](#footnote-ref-2)
