# Supplementary material for: Mind your Ps: A probabilistic model to aid the interpretation of molecular epidemiology data
Source: eBioMedicine. 2022 Apr 7;79:103989. doi: 10.1016/j.ebiom.2022.103989 (PMC9006250; doi:10.1016/j.ebiom.2022.103989)

**Supplement S4: Method protocol and example application**

[S4.1. Protocol 2](#_Toc88668895)

[1. Collect data 2](#_Toc88668896)

[2. Calculate 2](#_Toc88668897)

[3. Assess relatedness 2](#_Toc88668898)

[S4.2. Example application 3](#_Toc88668899)

[Assessing endemicity 3](#_Toc88668900)

[Assessing sample relatedness – case 1 4](#_Toc88668901)

[Assessing sample relatedness – case 2 5](#_Toc88668902)

[S4.3. Expected substitutions plots 6](#_Toc88668903)

[99% probability interval 6](#_Toc88668904)

[97% probability interval 9](#_Toc88668905)

[95% probability interval 12](#_Toc88668906)

[90% probability interval 15](#_Toc88668907)

[85% probability interval 18](#_Toc88668908)

[80% probability interval 21](#_Toc88668909)

# S4.1. Protocol

## Collect data

- 1. Samples 1 and 2 dates of symptom onset, sample collection or sample reception; sample 2 is presumed to be the most recent sample – t_1_ and t_2_
  2. Aligned sequences of the region of interest from samples 1 and 2 – s_1_ and s_2_
  3. Date of earliest sample associated with outbreak of interest – t_pCA_
  4. Substitution rate (substitutions/site.year) for the genomic region of the sequences (either published or obtained previously for a comparable set of data) – $\mu$

## Calculate

- 1. Estimated maximum time of divergence for samples 1 and 2 since the putative/presumed common ancestor or time of cumulative evolution, ∆t_CE_: ${\Delta t}_{CE}=\left( t_{1}-t_{pCA} \right)+\left( t_{2}-t_{pCA} \right)$
  2. Number of differences between s_1_ and s_2_, d (distance); differences can be counted according to this matrix:

|  | A | T | G | C | R | Y | W | M | S | K | B | V | H | D | N | - |
| --- | --- | --- | --- | --- | --- | --- | --- | --- | --- | --- | --- | --- | --- | --- | --- | --- |
| A | 0 | 1 | 1 | 1 | 0 | 1 | 0 | 0 | 1 | 1 | 1 | 0 | 0 | 0 | 0 | 1 |
| T | 1 | 0 | 1 | 1 | 1 | 0 | 0 | 1 | 1 | 0 | 0 | 1 | 0 | 0 | 0 | 1 |
| G | 1 | 1 | 0 | 1 | 0 | 1 | 1 | 1 | 0 | 0 | 0 | 0 | 1 | 0 | 0 | 1 |
| C | 1 | 1 | 1 | 0 | 1 | 0 | 1 | 0 | 0 | 1 | 0 | 0 | 0 | 1 | 0 | 1 |
| R | 0 | 1 | 0 | 1 | 0 | 1 | 0 | 0 | 0 | 0 | 0 | 0 | 0 | 0 | 0 | 1 |
| Y | 1 | 0 | 1 | 0 | 1 | 0 | 0 | 0 | 0 | 0 | 0 | 0 | 0 | 0 | 0 | 1 |
| W | 0 | 0 | 1 | 1 | 0 | 0 | 0 | 0 | 1 | 0 | 0 | 0 | 0 | 0 | 0 | 1 |
| M | 0 | 1 | 1 | 0 | 0 | 0 | 0 | 0 | 0 | 1 | 0 | 0 | 0 | 0 | 0 | 1 |
| S | 1 | 1 | 0 | 0 | 0 | 0 | 1 | 0 | 0 | 0 | 0 | 0 | 0 | 0 | 0 | 1 |
| K | 1 | 0 | 0 | 1 | 0 | 0 | 0 | 1 | 0 | 0 | 0 | 0 | 0 | 0 | 0 | 1 |
| B | 1 | 0 | 0 | 0 | 0 | 0 | 0 | 0 | 0 | 0 | 0 | 0 | 0 | 0 | 0 | 1 |
| V | 0 | 1 | 0 | 0 | 0 | 0 | 0 | 0 | 0 | 0 | 0 | 0 | 0 | 0 | 0 | 1 |
| H | 0 | 0 | 1 | 0 | 0 | 0 | 0 | 0 | 0 | 0 | 0 | 0 | 0 | 0 | 0 | 1 |
| D | 0 | 0 | 0 | 1 | 0 | 0 | 0 | 0 | 0 | 0 | 0 | 0 | 0 | 0 | 0 | 1 |
| N | 0 | 0 | 0 | 0 | 0 | 0 | 0 | 0 | 0 | 0 | 0 | 0 | 0 | 0 | 0 | 1 |
| - | 1 | 1 | 1 | 1 | 1 | 1 | 1 | 1 | 1 | 1 | 1 | 1 | 1 | 1 | 1 | 0 |

## Assess relatedness

- 1. Input substitution rate and number of sites in the alignment in the highlighted green cells in the accompanying spreadsheet (Supplement S2)
  2. Select the Poisson probability interval for which to calculate the maximum number of expected substitutions (e.g., 95%)
  3. Find the row corresponding to the ∆t_CE_ in the Poisson CDF table
  4. Obtain the maximum number of the substitutions expected for the time frame: the number of substitutions corresponds to the first dark grey cell in the row found in 3.3

Or

- 1. Find the point (∆t_CE_, d) in the expected substitution plots provided (plots for measles genotypes B3, D4 and D8 N-450 and MF-NCR genomic regions at different Poisson distribution probability intervals are included below)
  2. Determine if the pair is plotted
     1. below the maximum number of expected substitutions – samples likely to have a common ancestor in the time frame considered)
     2. above the expected substitution range – samples are unlikely to have diverged from a common ancestor within the given time frame

# S4.2. Example application

## Assessing endemicity

A measles outbreak of MeV MVs/Dublin.IRL/8.16[B3] strain occurred in the UK in 2016-17. To address endemicity in 2017, the first (sample 1) and last samples (sample 2) sharing the same N-450 sequence in the cluster of cases spanning the time period can be analysed. Because the earliest and latest samples are over one year apart, the putative common ancestor can be approximated to sample 1 given that missing of few cases would have a small impact over the time sample 2 had to diverge. Following the protocol described in the S4.2 section:

- Sample 1:
  - N-450 sequence identical to that of MVs/Dublin.IRL/8.16
  - Week 16 of 2016
- Sample 2:
  - N-450 sequence identical to that of MVs/Dublin.IRL/8.16
  - Week 21 of 2017

1. As the N-450 sequences are identical, assessment can be based solely on the aligned MF-NCR sequences.
2. Distance between samples 1 and 2’s MF-NCR sequences: 9 substitutions.
3. Time between samples 1 and 2: 57 weeks.
4. Choose probability interval, α (higher values lead to higher confidence in the prediction): 95% or 0.95.


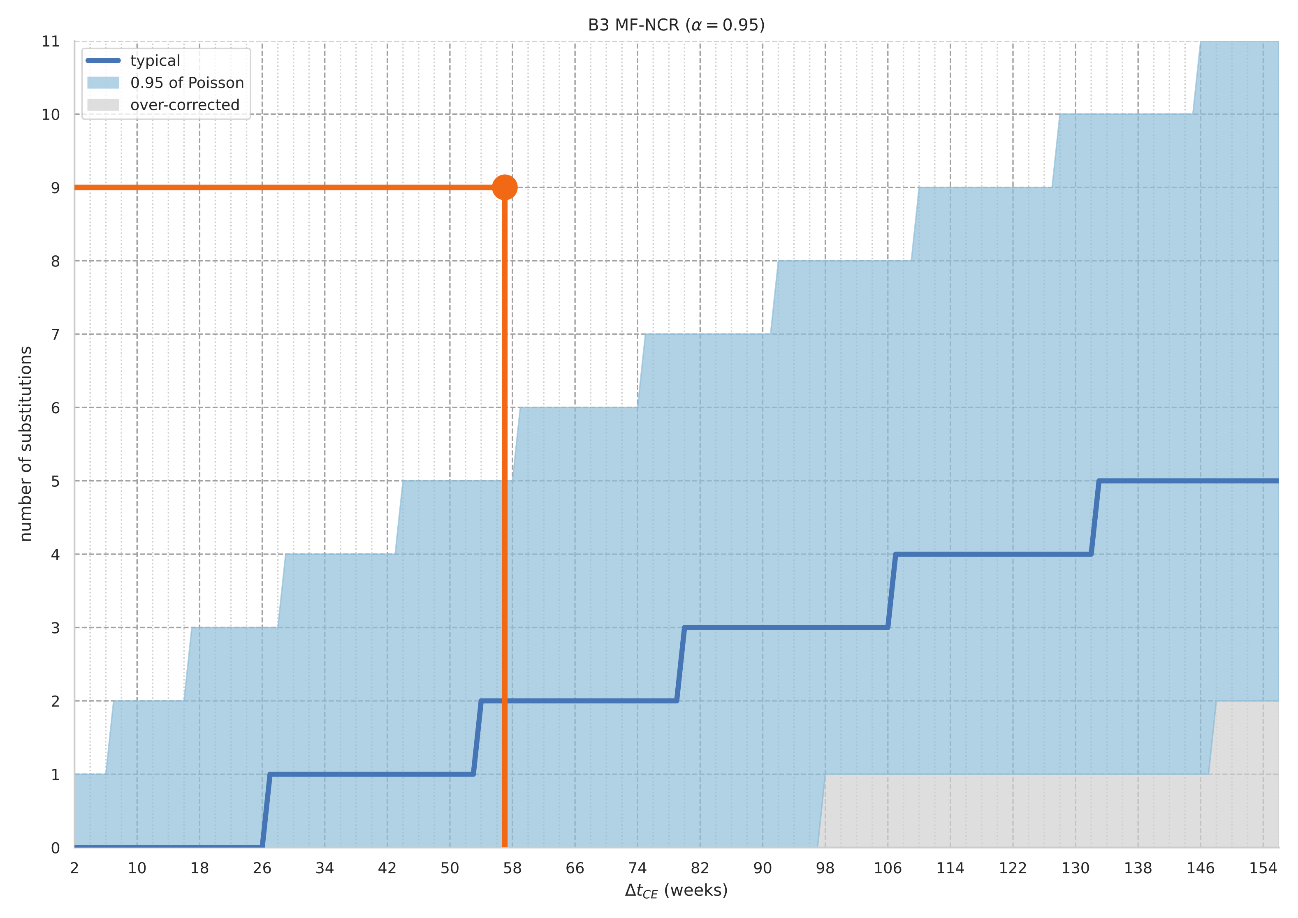


1. Upper limit of expected substitutions at a cumulative evolution time of 57 weeks: 5 substitutions
2. Distance of 9 substitutions is greater than 5 – it is unlikely that sample 2 is a direct descendant of sample 1 within the time frame
3. Unlikely endemic transmission

The BEAST-estimated time-scaled phylogeny including these samples is shown in Figure 2. Sample 1 is in the single leaf node sharing a BEAST-estimated MRCA with phylogenetic clusters A-D. Sample 2 is the latest sample in cluster C. The phylodynamic tree supports the conclusion that sample 2 is unlikely to have descended from sample 1 in the time between the samples.

## Assessing sample relatedness – case 1

A second use case is in supporting cluster identification when characterising outbreaks. Continuing with the analysis of the MeV Dublin 2016 strain outbreak in the UK during 2016-17, if we were particularly interested in samples 1 and 2 in that outbreak, for which there is no epidemiology data, and wanted to assess whether the samples could be descendent of a common ancestor within the known period of transmission in the UK, the approach presented in this study could be employed.

- Sample 1:
  - N-450 sequence identical to that of MVs/Dublin.IRL/8.16
  - Week 1 of 2017
- Sample 2:
  - N-450 sequence identical to that of MVs/Dublin.IRL/8.16
  - Week 21 of 2017
- Putative ancestor
  - first detected sequence with MVs/Dublin.IRL/8.16[B3]’s N-450 sequence (the first measles case in the region in the time period could be selected if no sequence data available)
  - Week 16 of 2016, earliest sample that may be ancestor of samples 1 and 2

1. Again, the N-450 sequences were identical, so the analysis can be based on the aligned MF-NCR sequences.
2. Distance between samples 1 and 2’s MF-NCR sequences: 9 substitutions.
3. Time between sample 1 and putative ancestor + time between sample 2 and putative ancestor: 94 weeks.
4. Choose probability interval, α (higher values should lead to higher confidence in the prediction): 95% or 0.95.


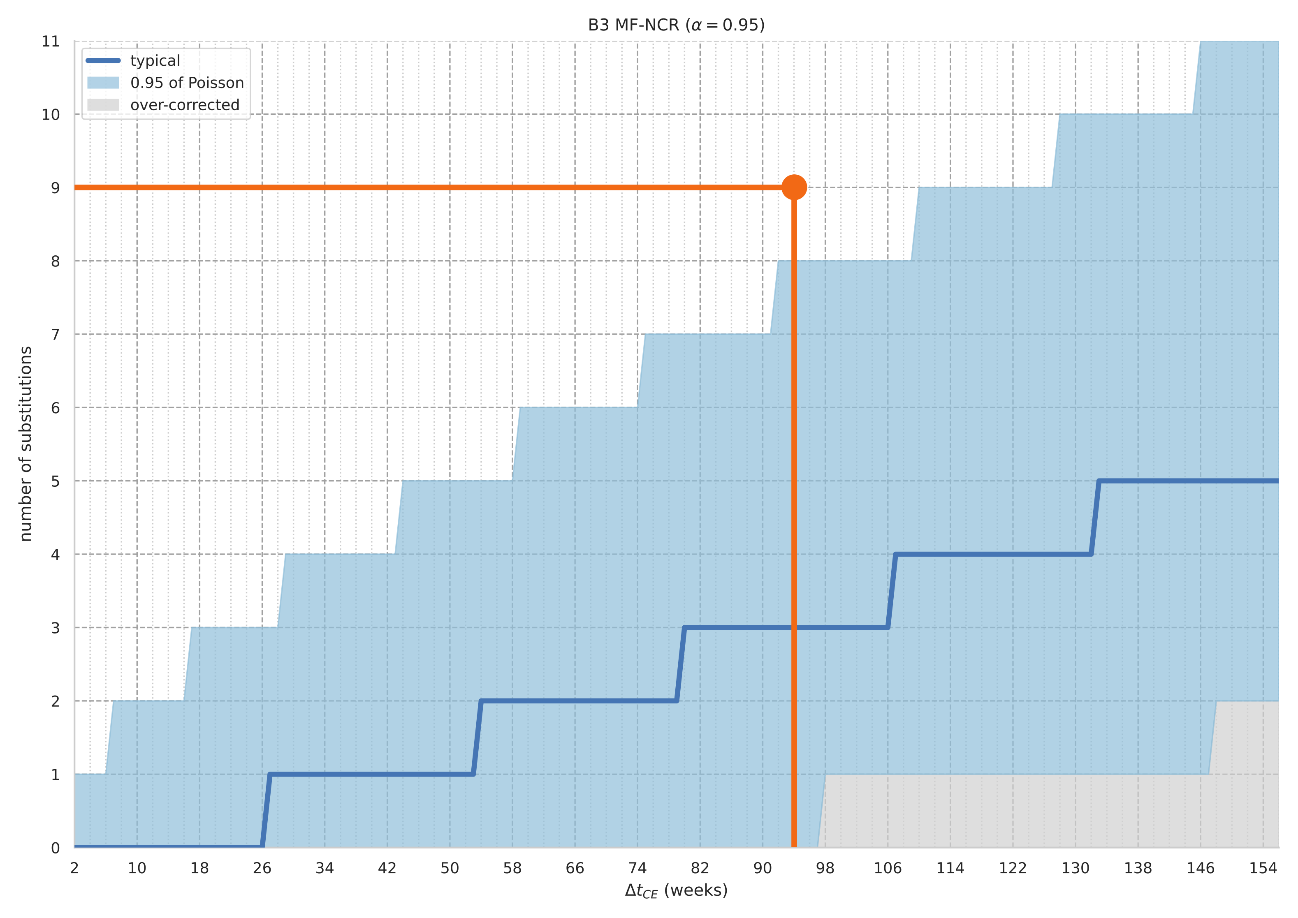


1. Upper limit of expected substitutions at a cumulative evolution time of 94 weeks: 8 substitutions.
2. Distance of 9 substitutions is greater than 8 – it is unlikely that samples 1 and 2 have descended from the putative ancestor sample.
3. Unlikely that samples 1 and 2 are related in the time frame considered.

The putative common ancestor is the same sample as sample 1 in the previous example. Sample 1 is earliest UK sample in phylogenetic cluster A marked in Figure 2. Sample 2 is the latest UK sample in cluster C. The BEAST estimate of the phylogeny places samples 1 and 2 in separate phylogenetic clusters, conforming with the model prediction.

## Assessing sample relatedness – case 2

Selecting a different pair of samples in the same outbreak.

- Sample 1:
  - N-450 sequence identical to that of MVs/Dublin.IRL/8.16
  - Week 1 of 2017
- Sample 2:
  - N-450 sequence identical to that of MVs/Dublin.IRL/8.16
  - Week 40 of 2017
- Putative ancestor
  - first detected sequence with MVs/Dublin.IRL/8.16[B3]’s N-450 sequence (the first measles case in the region in the time period could be selected if no sequence data available)
  - Week 16 of 2016, earliest sample that may be ancestor of samples 1 and 2

1. Obtain aligned MF-NCR sequences.
2. Distance between samples 1 and 2’s MF-NCR sequences: 6 substitutions.
3. Time between sample 1 and putative ancestor + time between sample 2 and putative ancestor: 113 weeks.
4. Choose probability interval (higher values should lead to higher confidence in the prediction): 95% or 0.95.


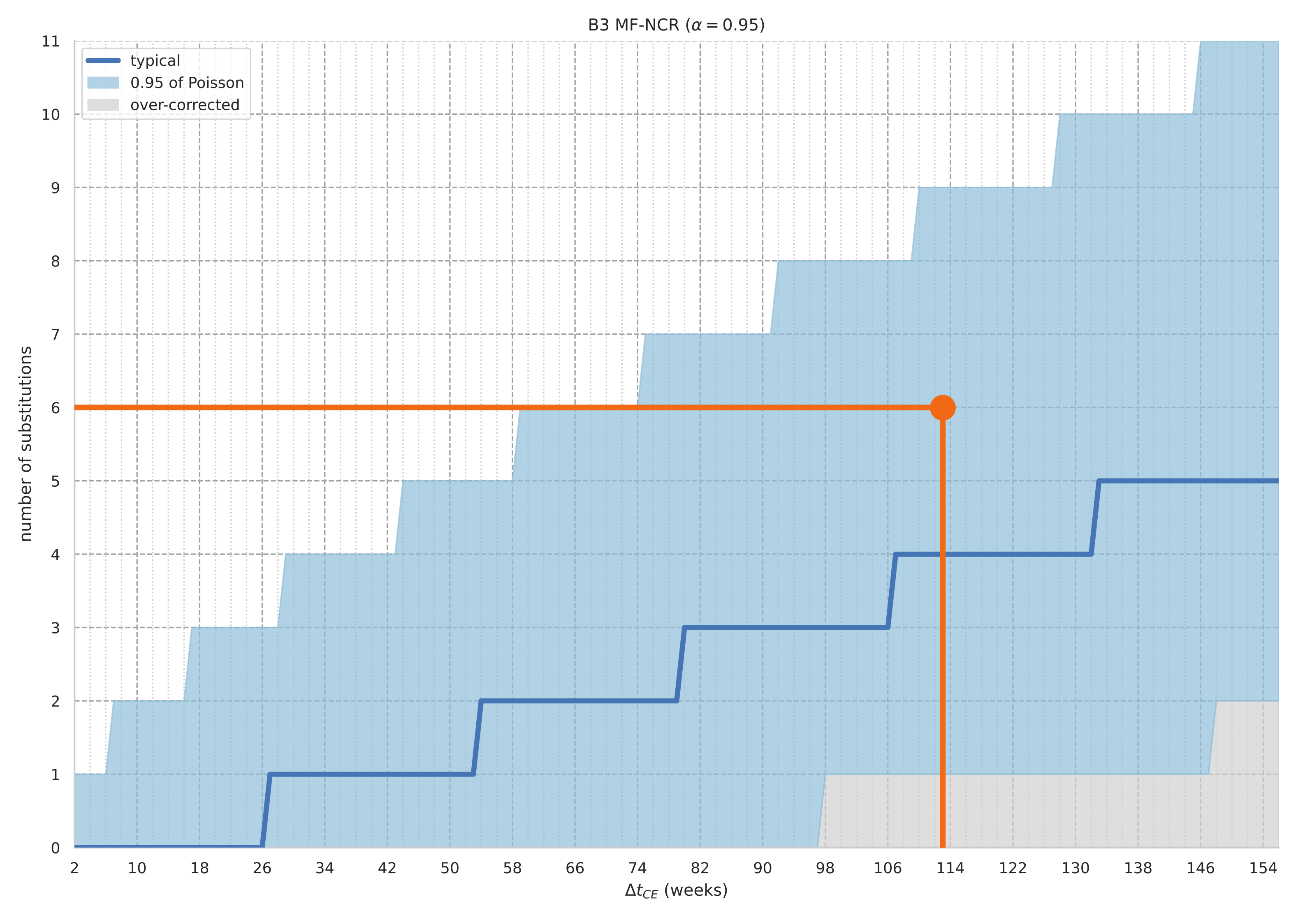


1. Upper limit of expected substitutions at a cumulative evolution time of 113 weeks: 9 substitutions.
2. Distance of 6 substitutions is lesser than 9 – we cannot exclude that samples 1 and 2 have descended from the putative ancestor sample.
3. Cannot exclude that samples 1 and 2 are related in the time frame considered.

The putative common ancestor is the same as in the examples above. Samples 1 and 2 are the earliest and latest samples in cluster A of Figure 2.

# S4.3. Expected substitutions plots

## 99% probability interval


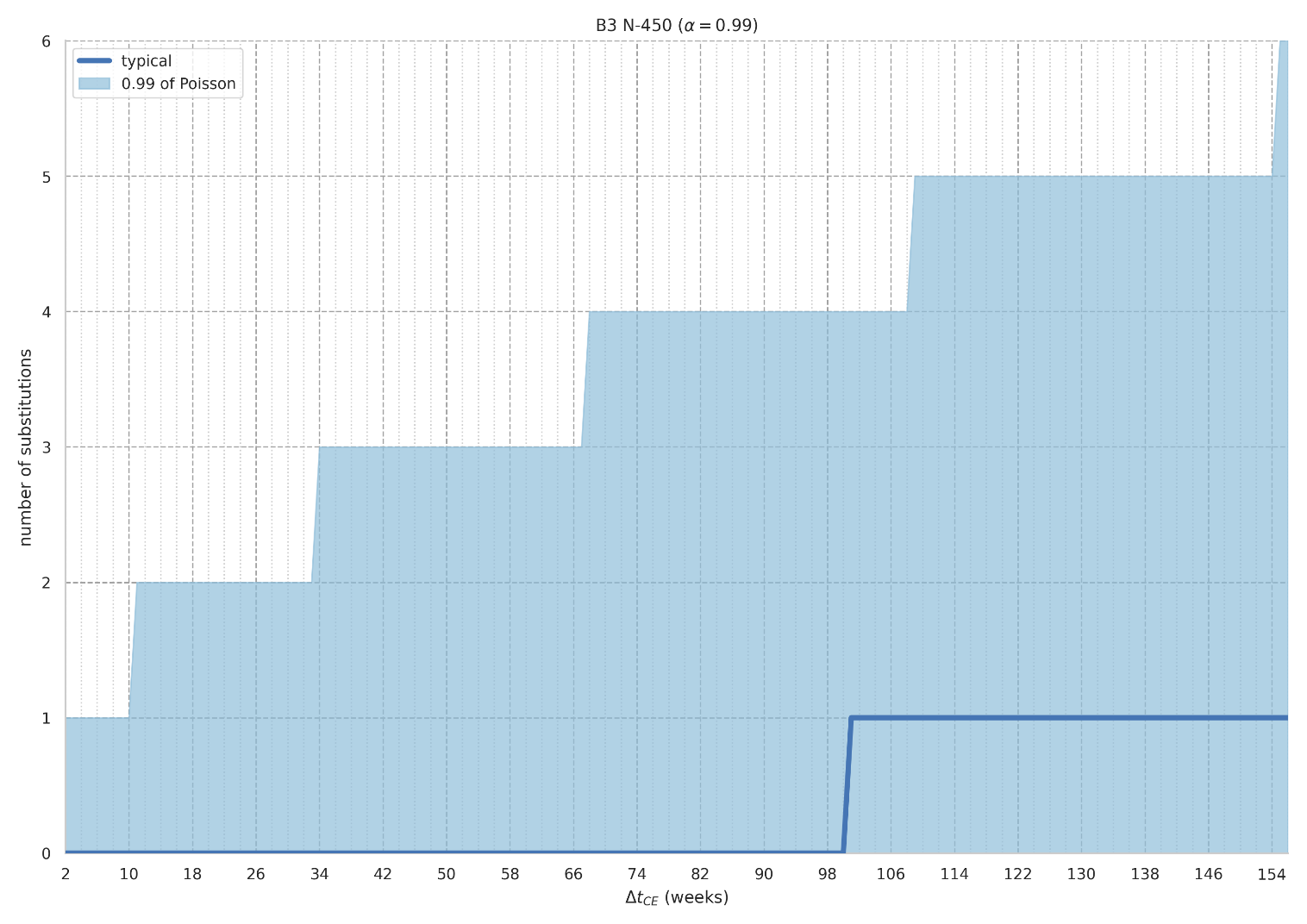

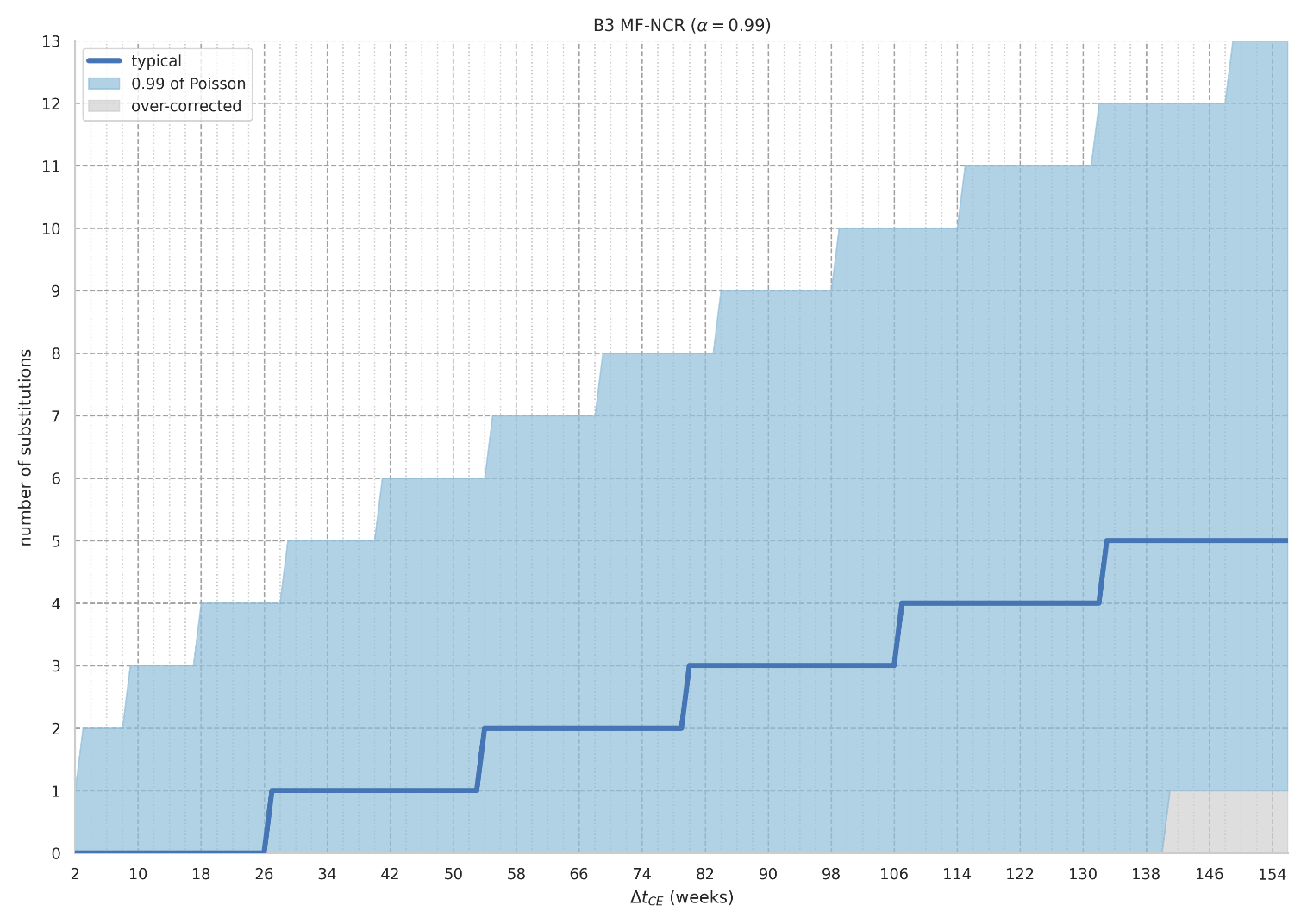


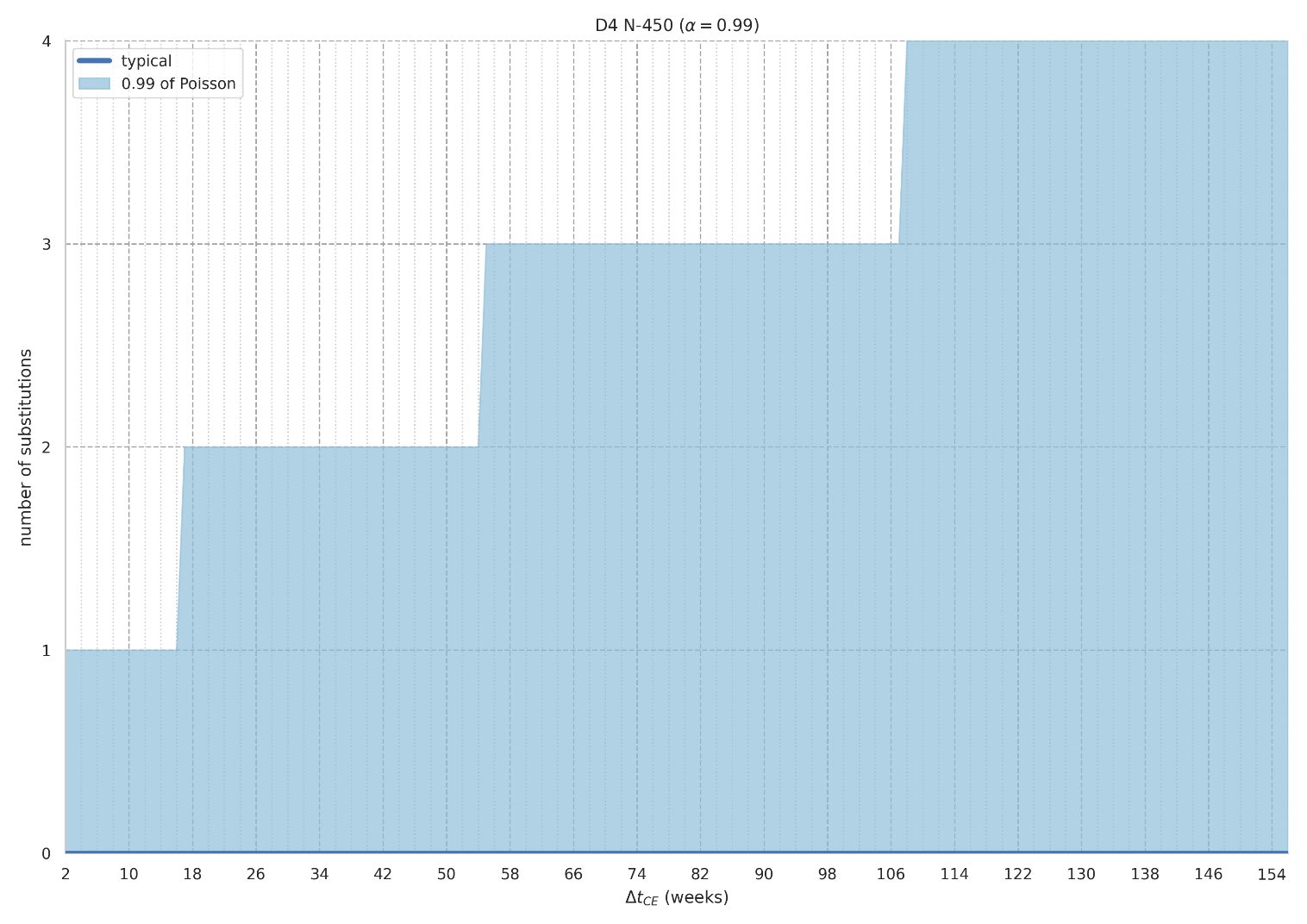

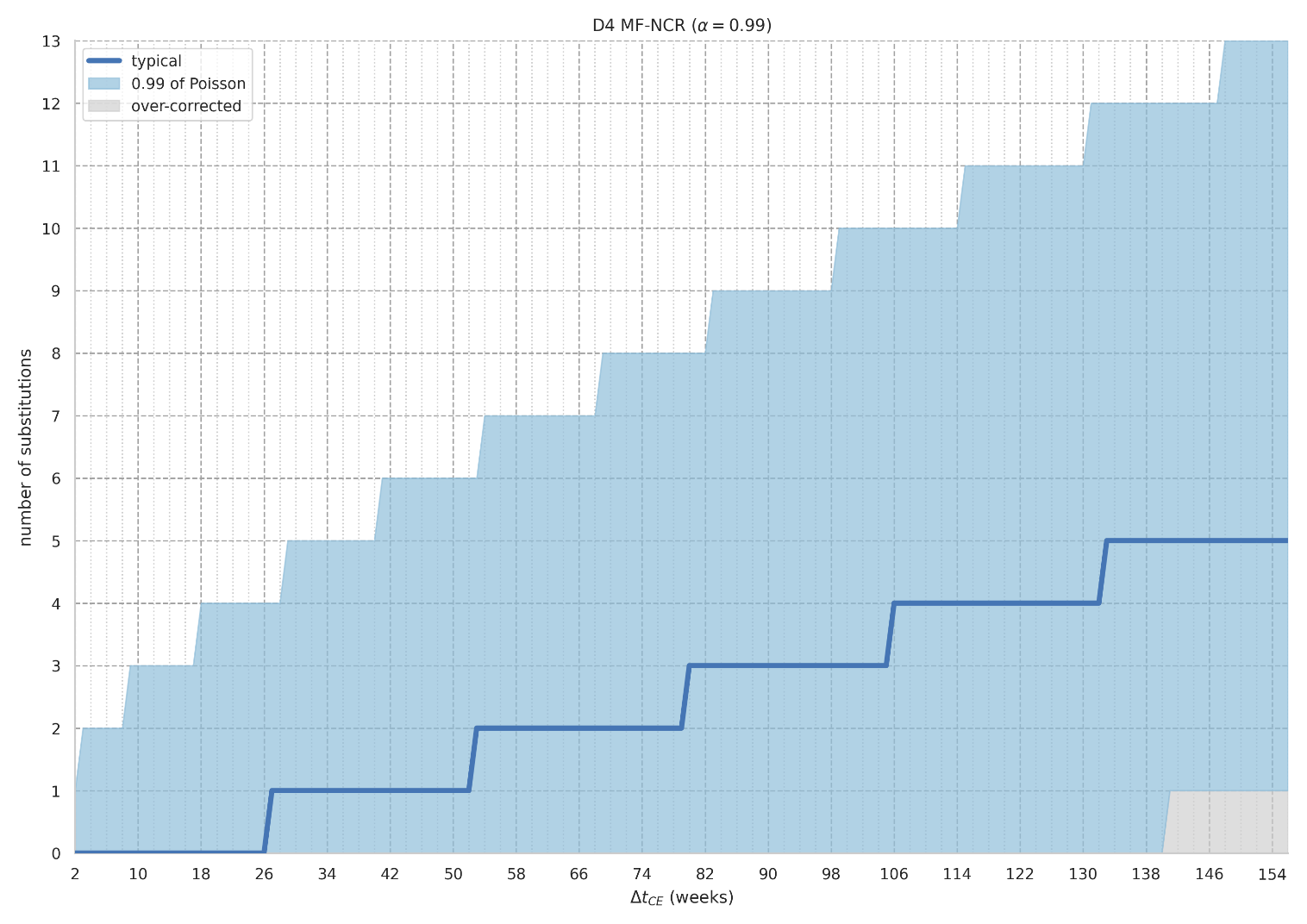


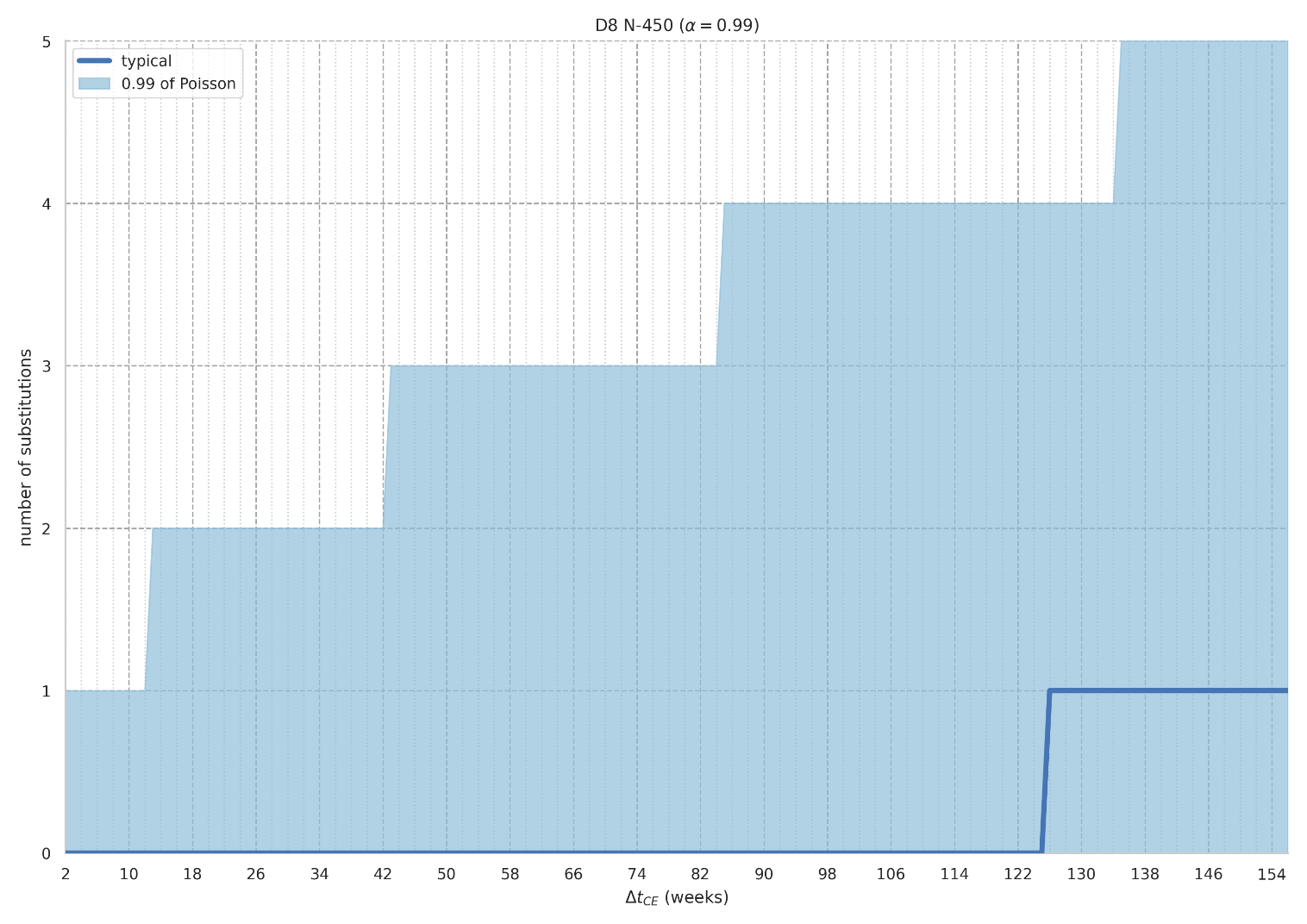

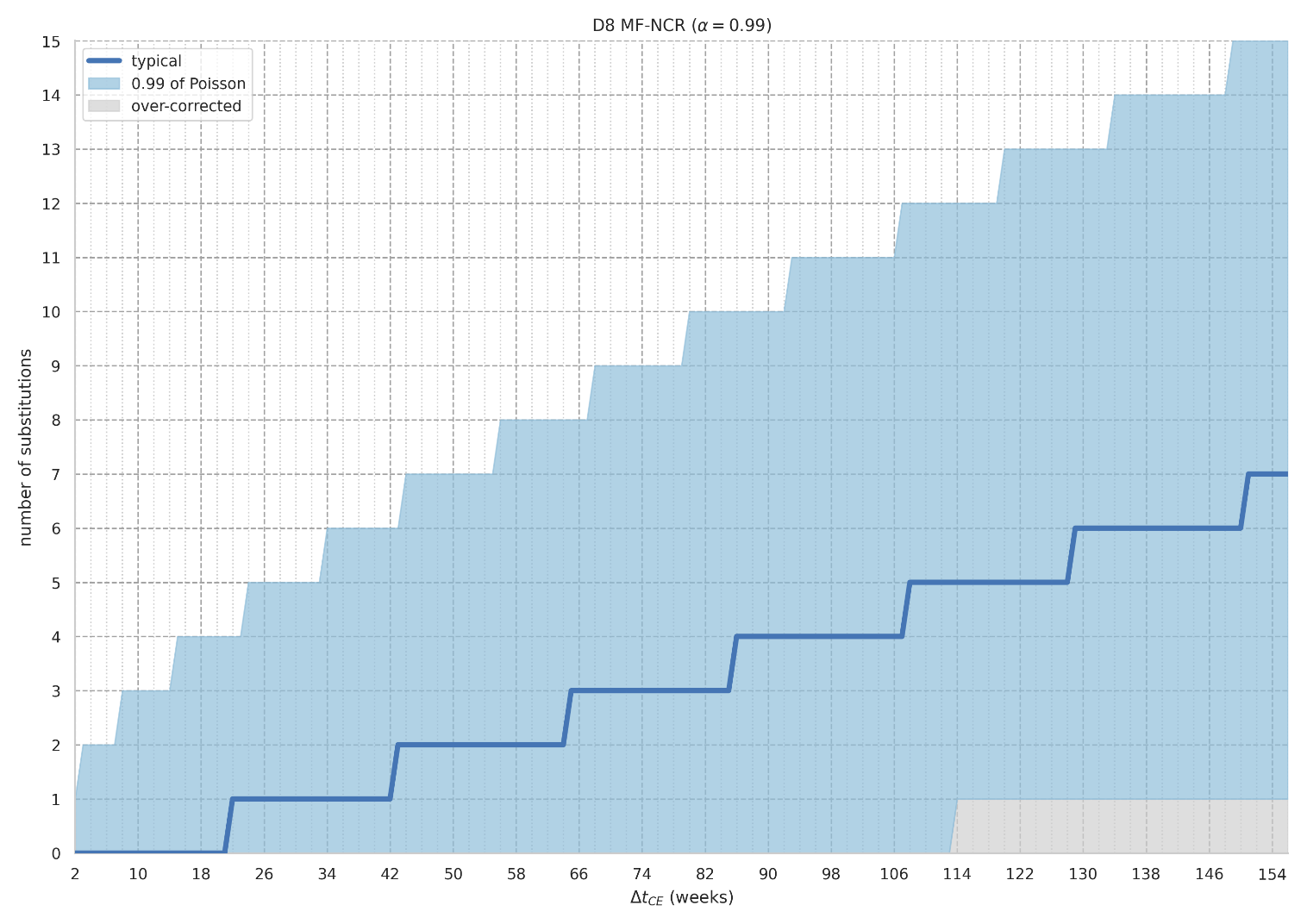


## 97% probability interval


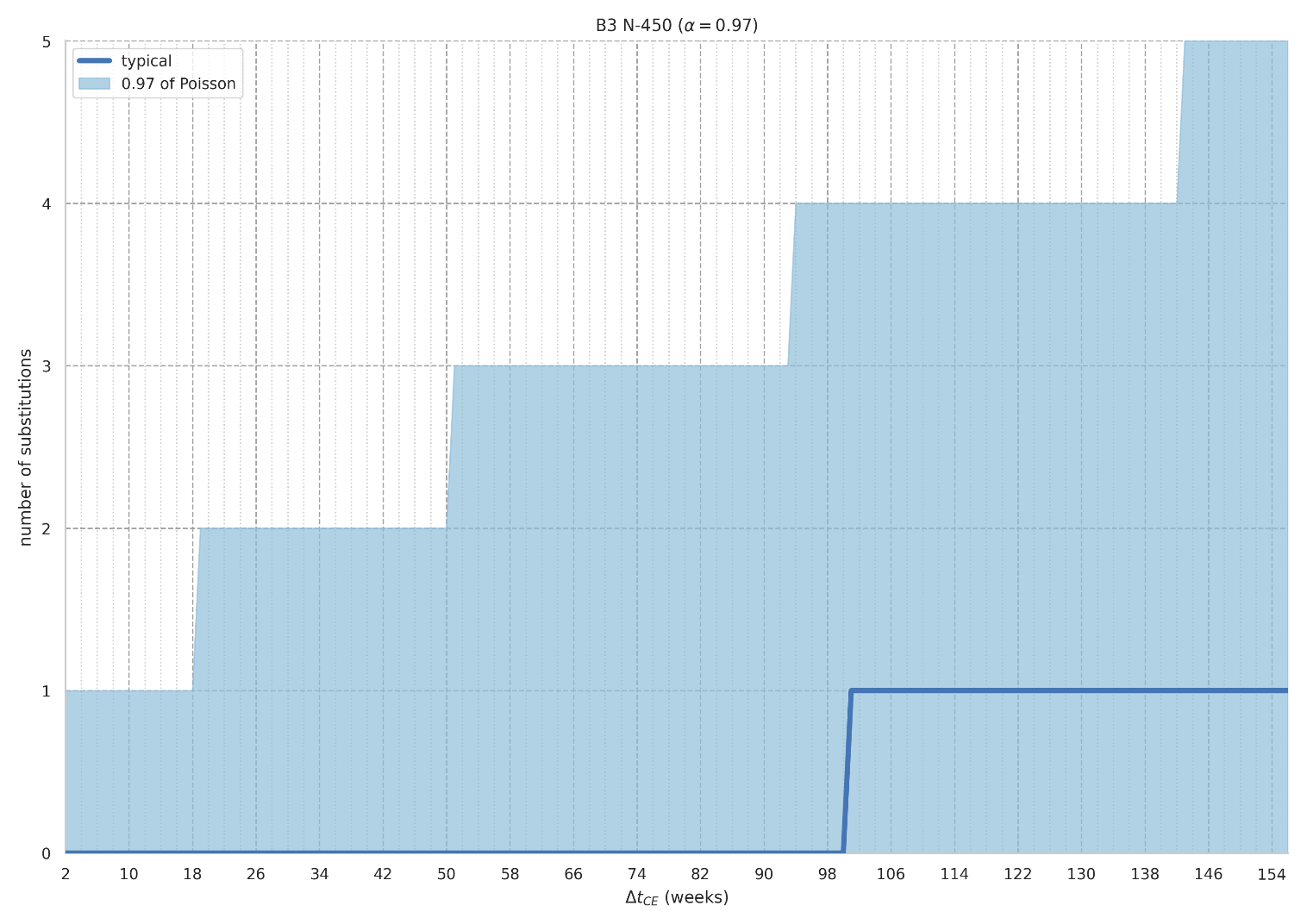

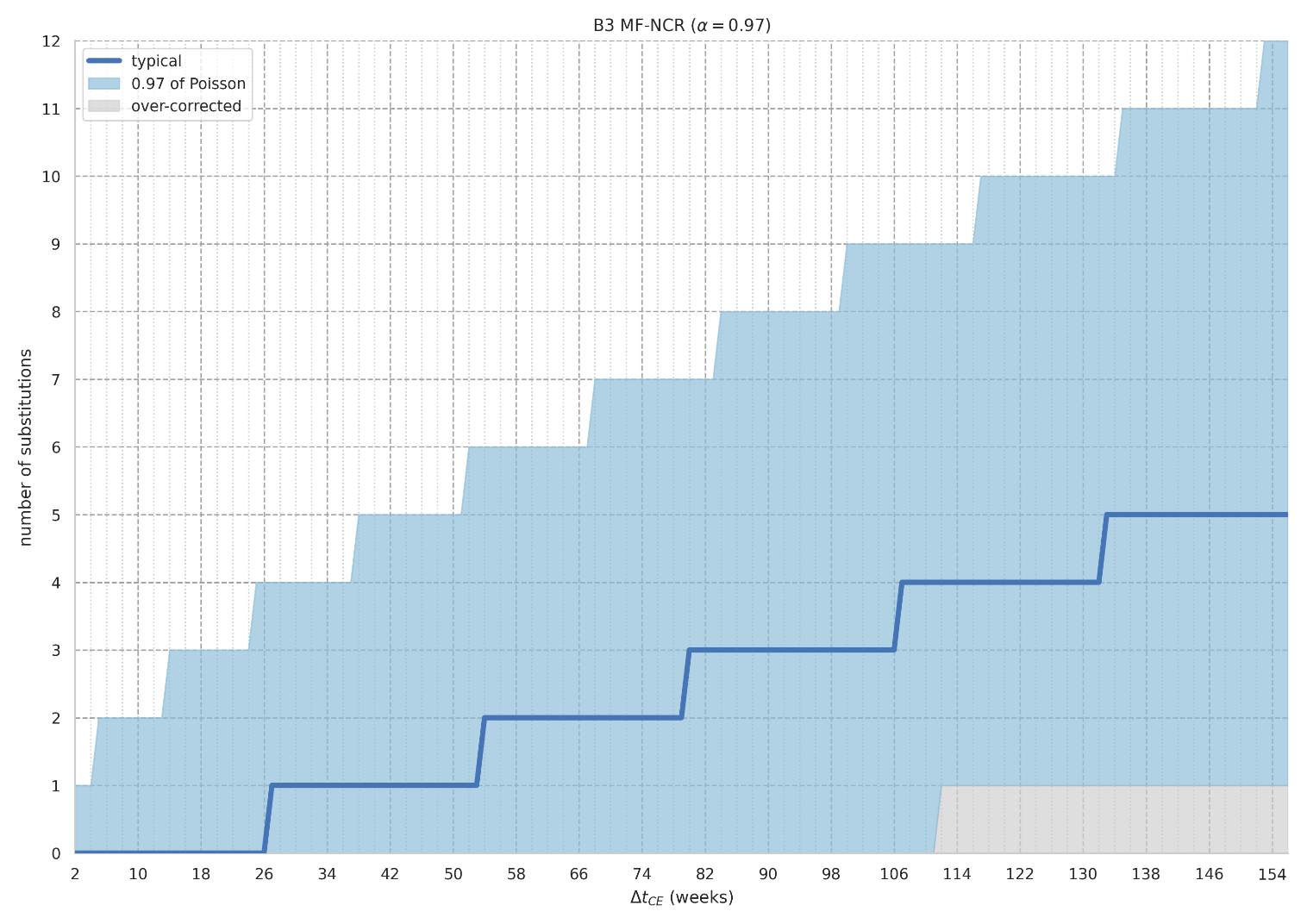


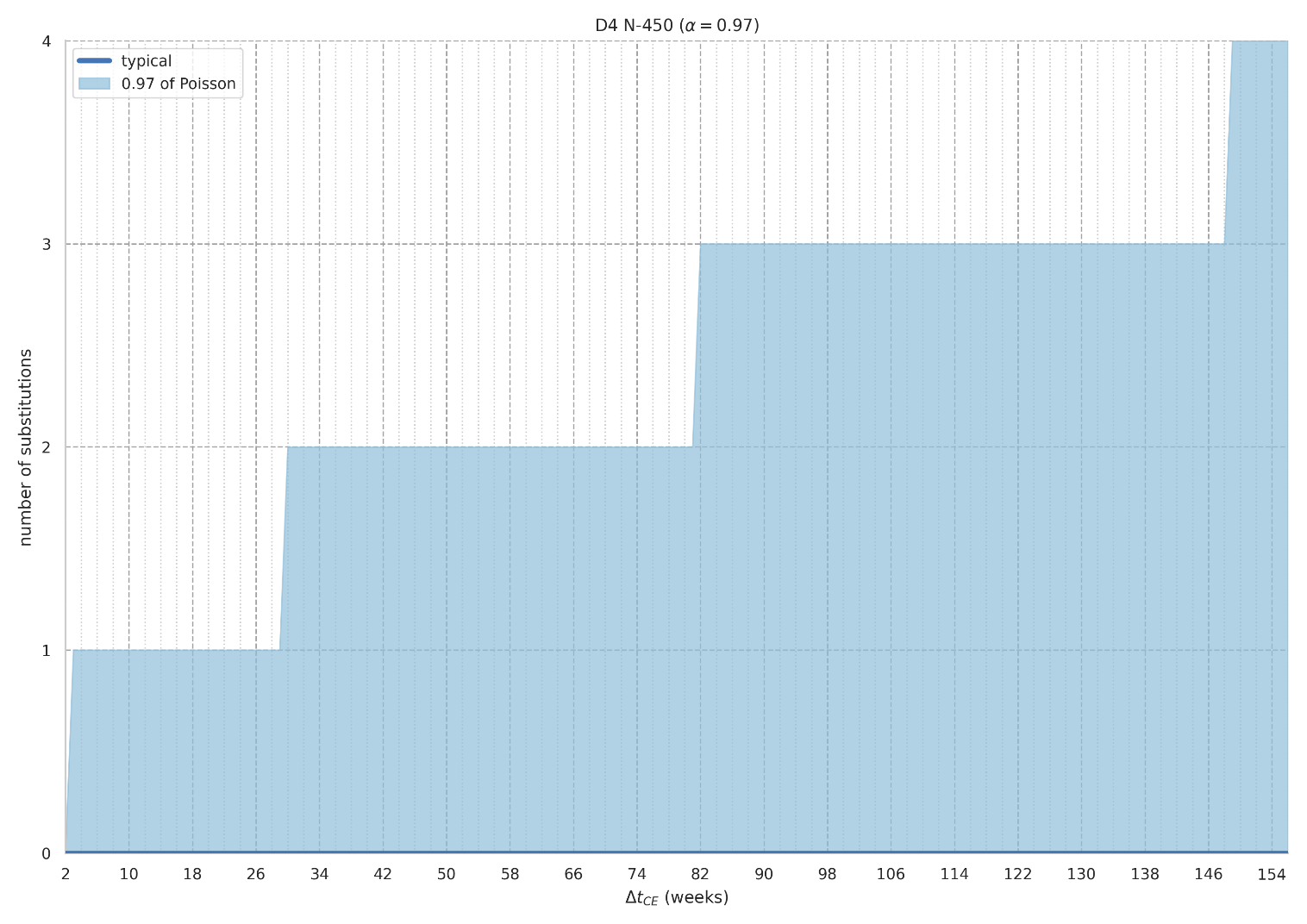

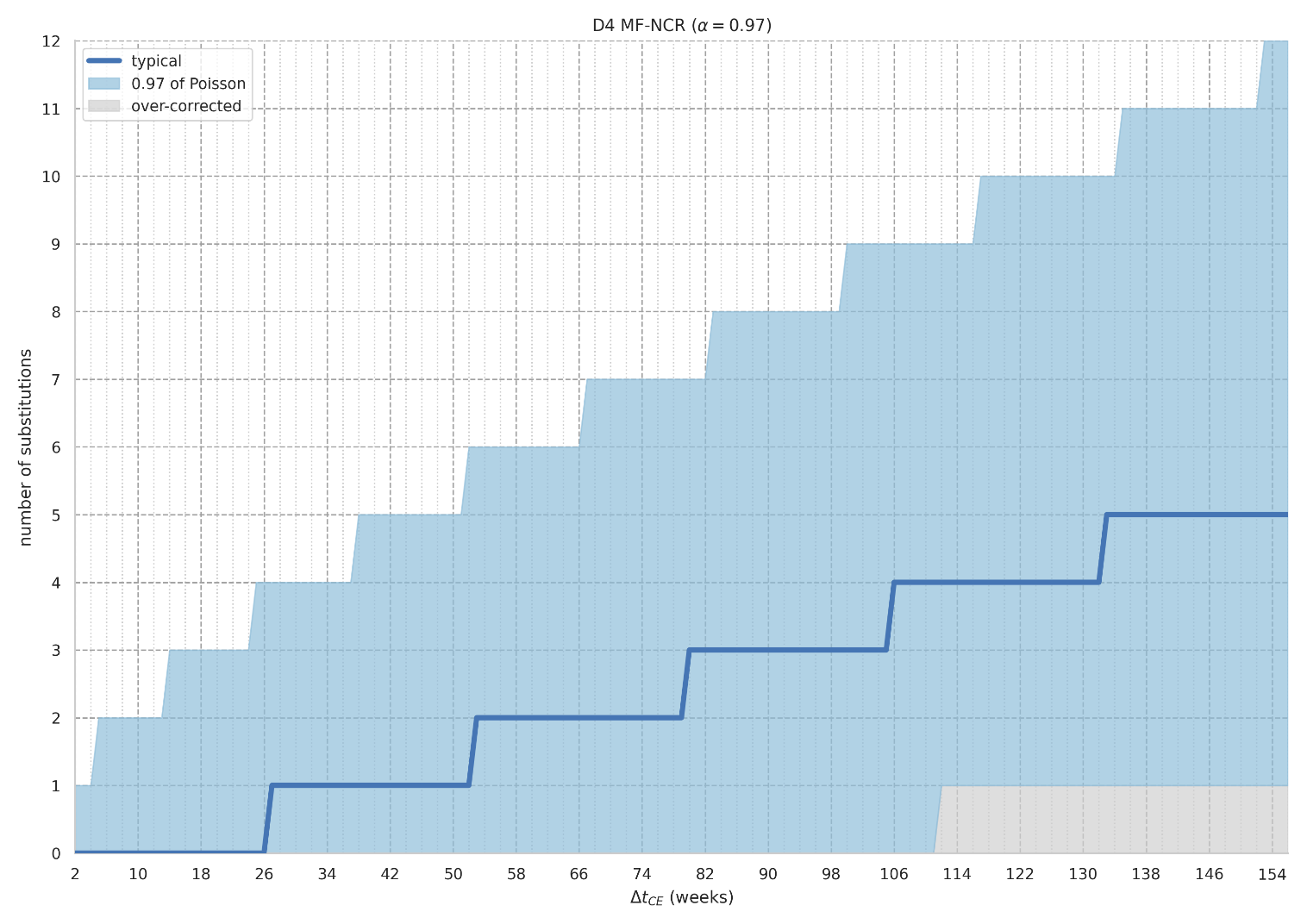

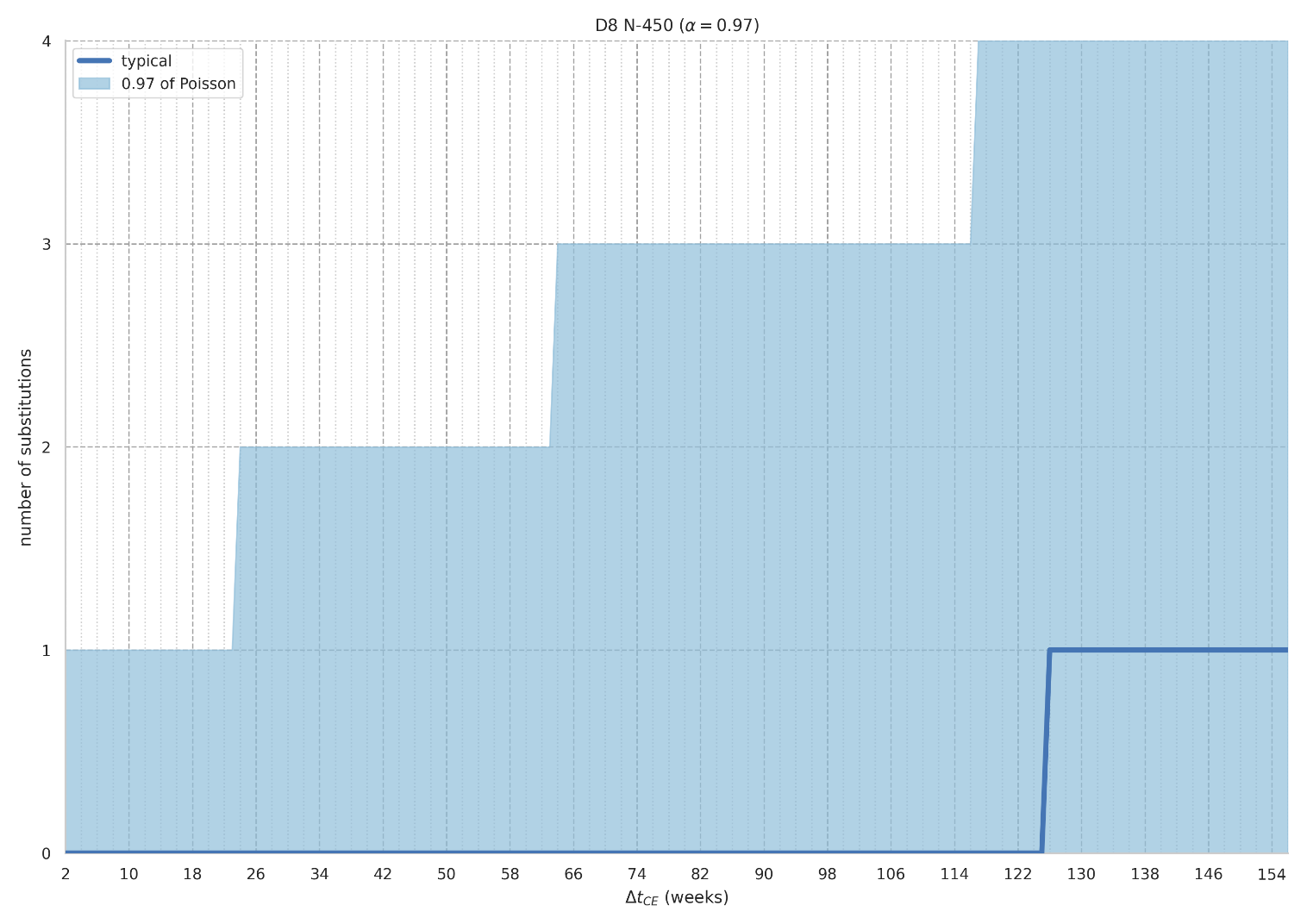

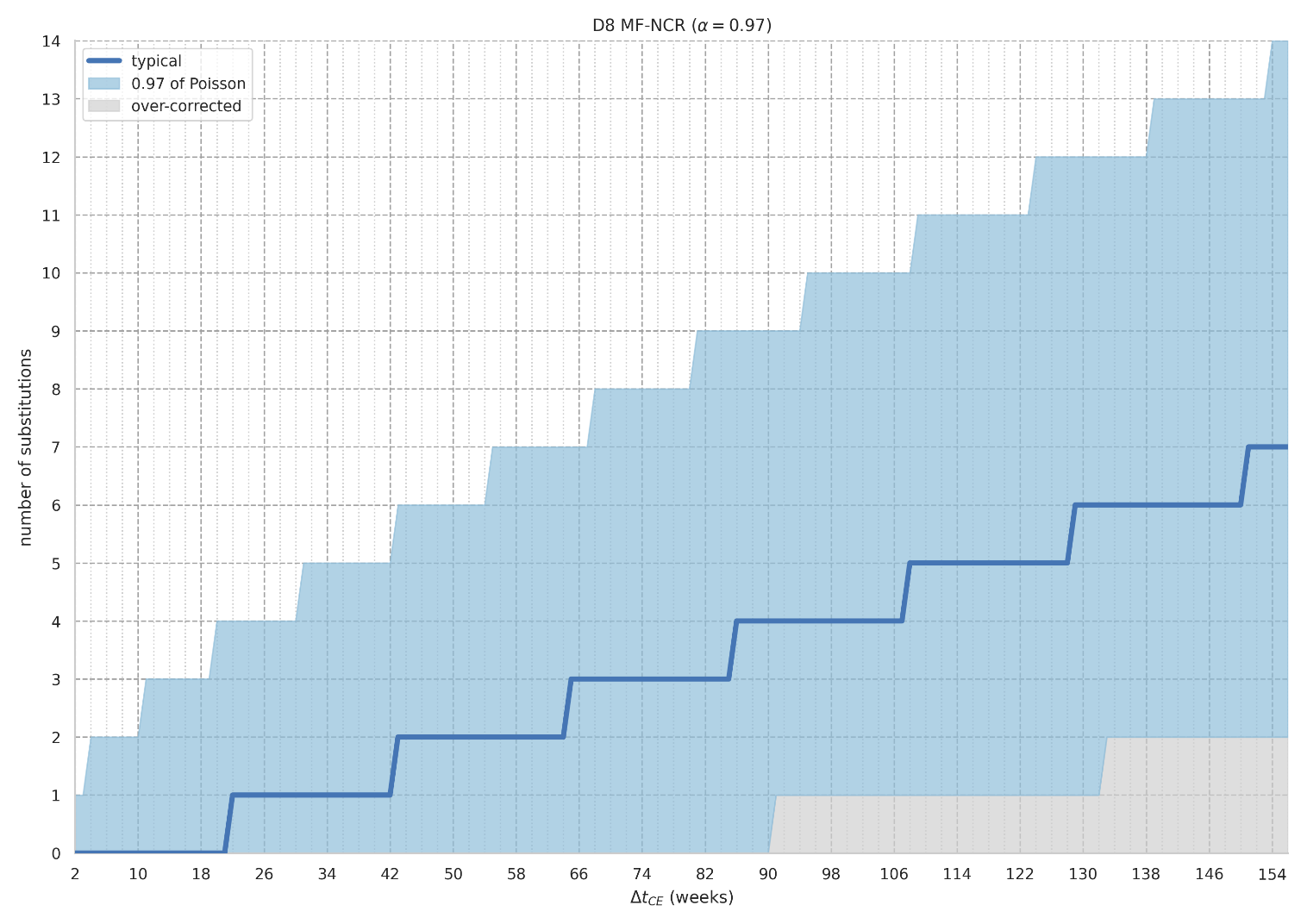


## 95% probability interval


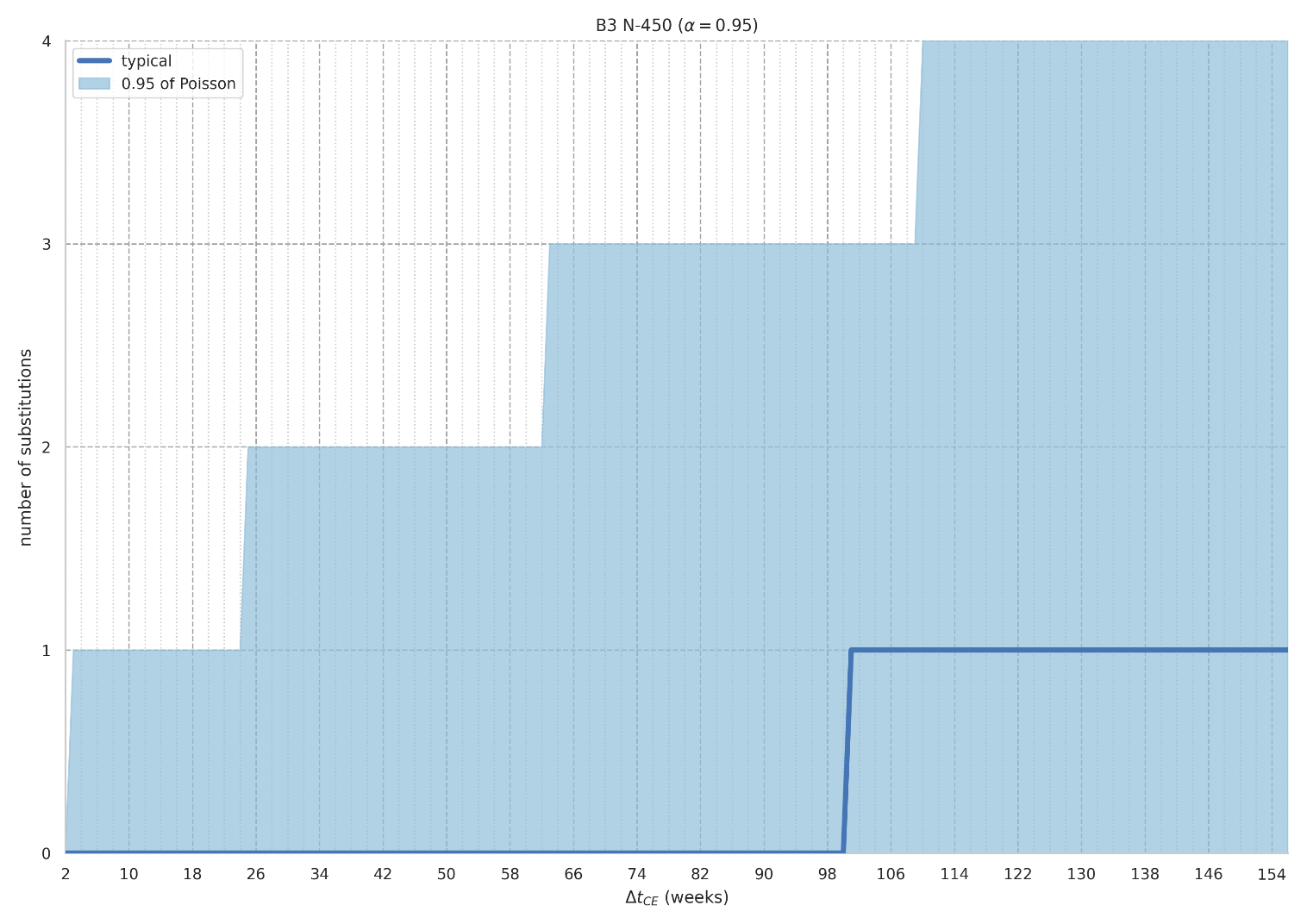

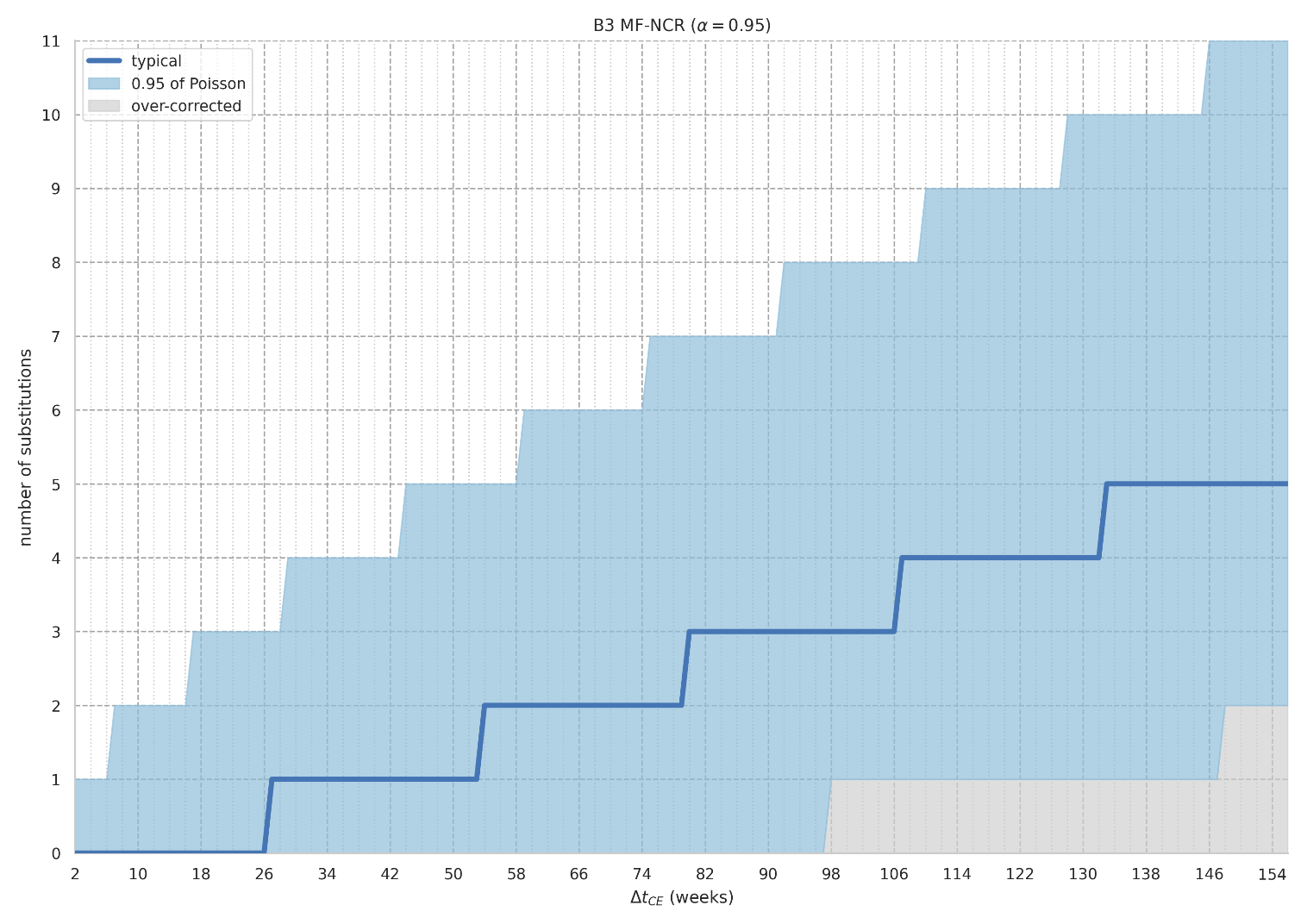

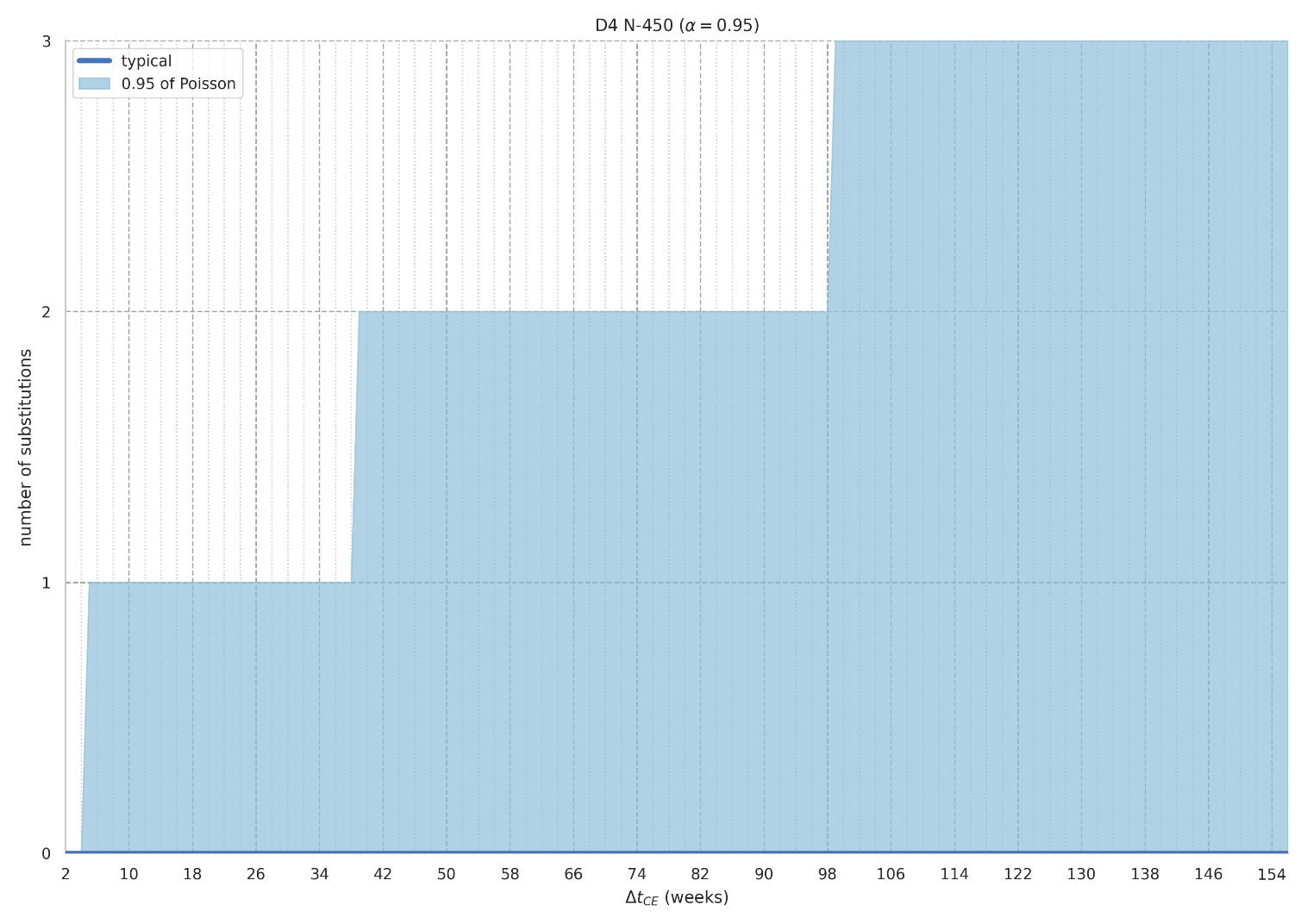

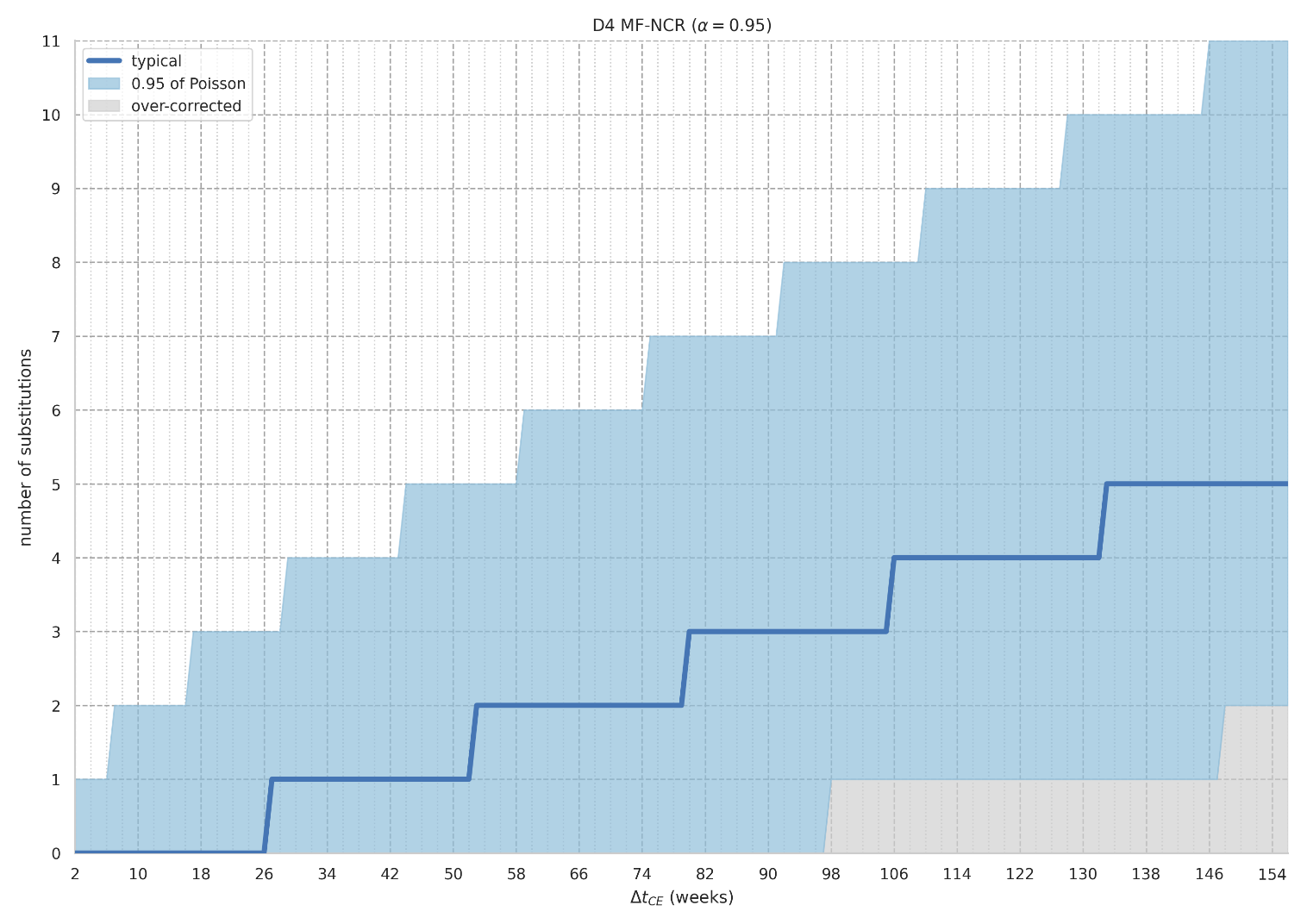

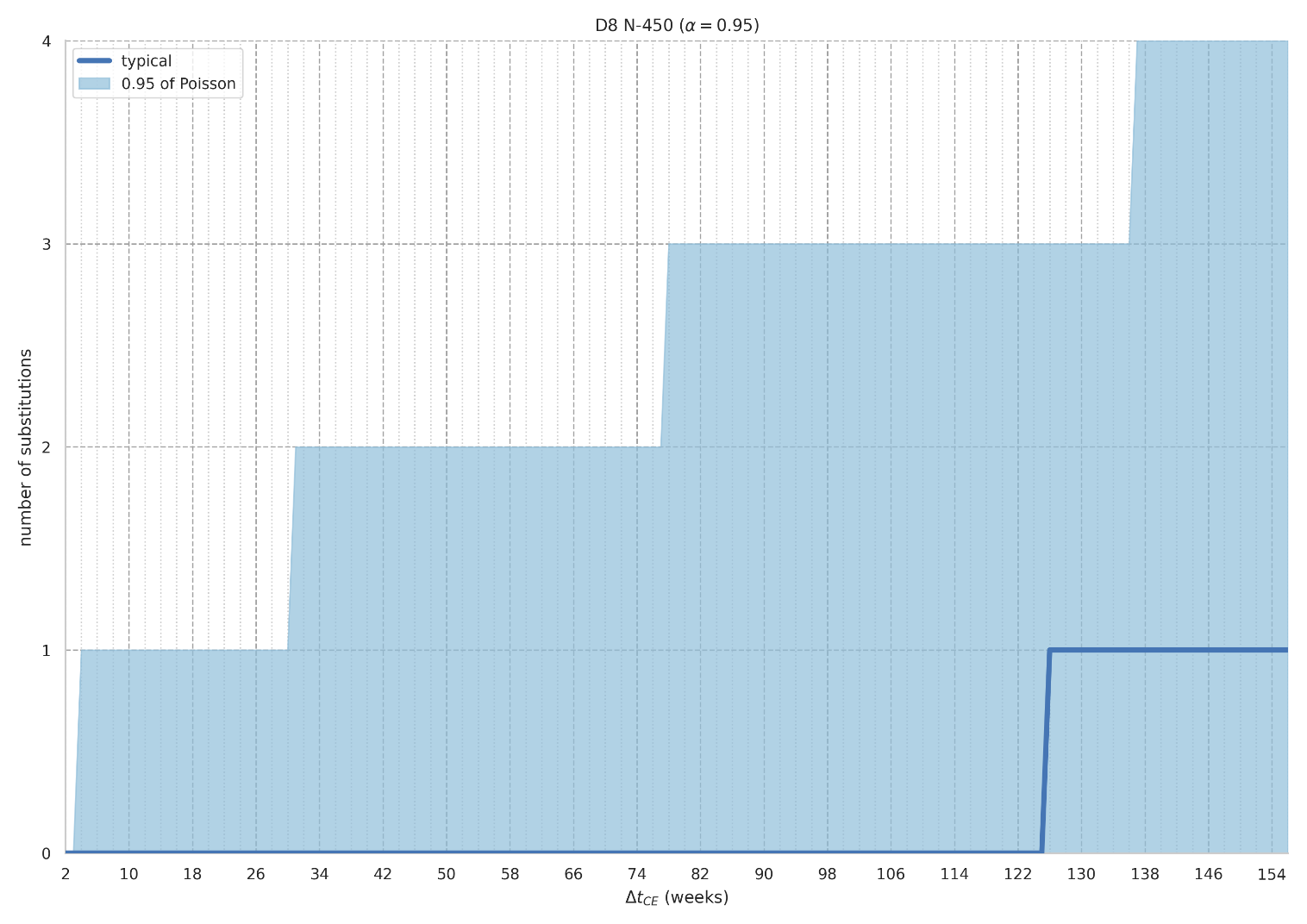

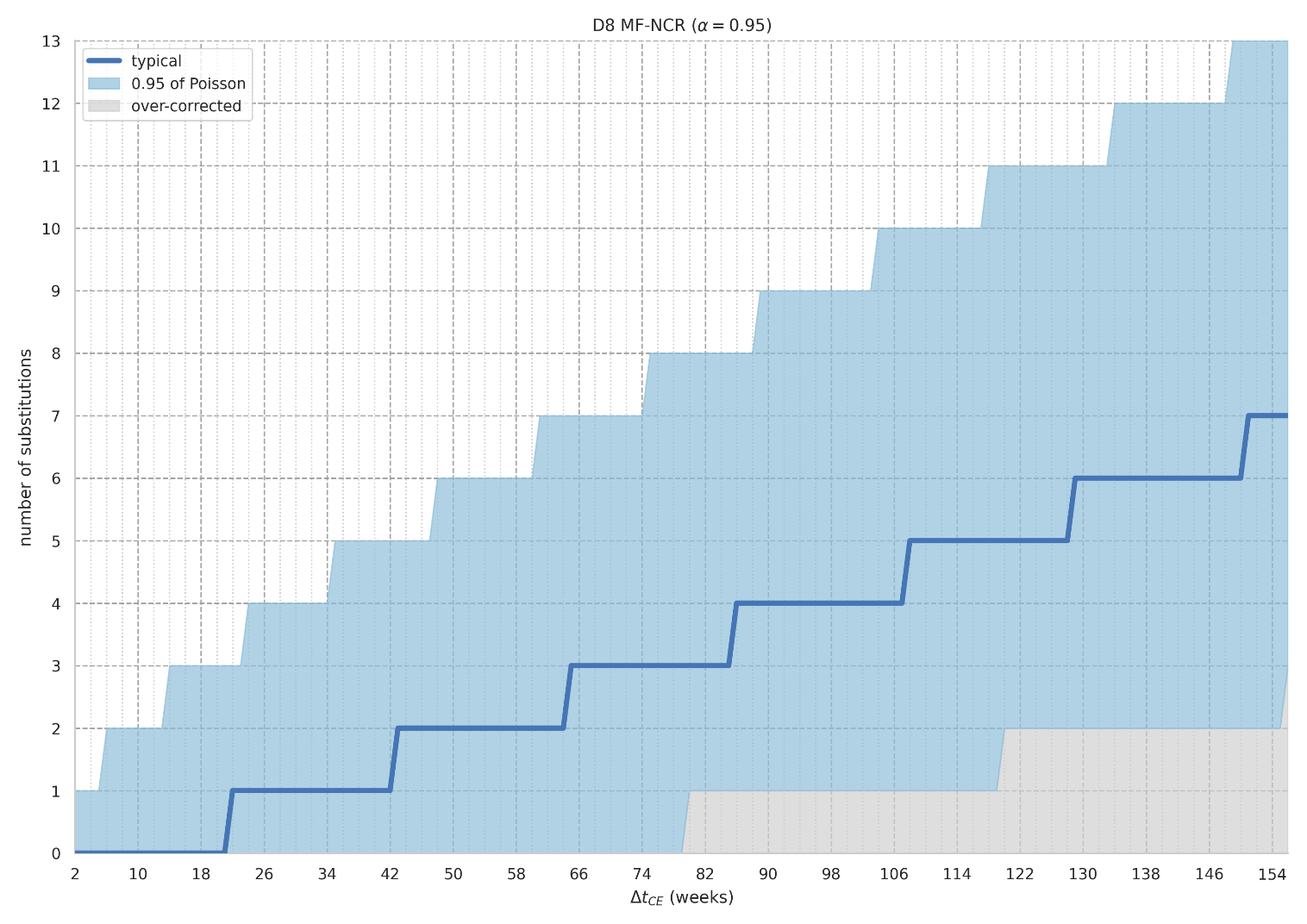


## 90% probability interval


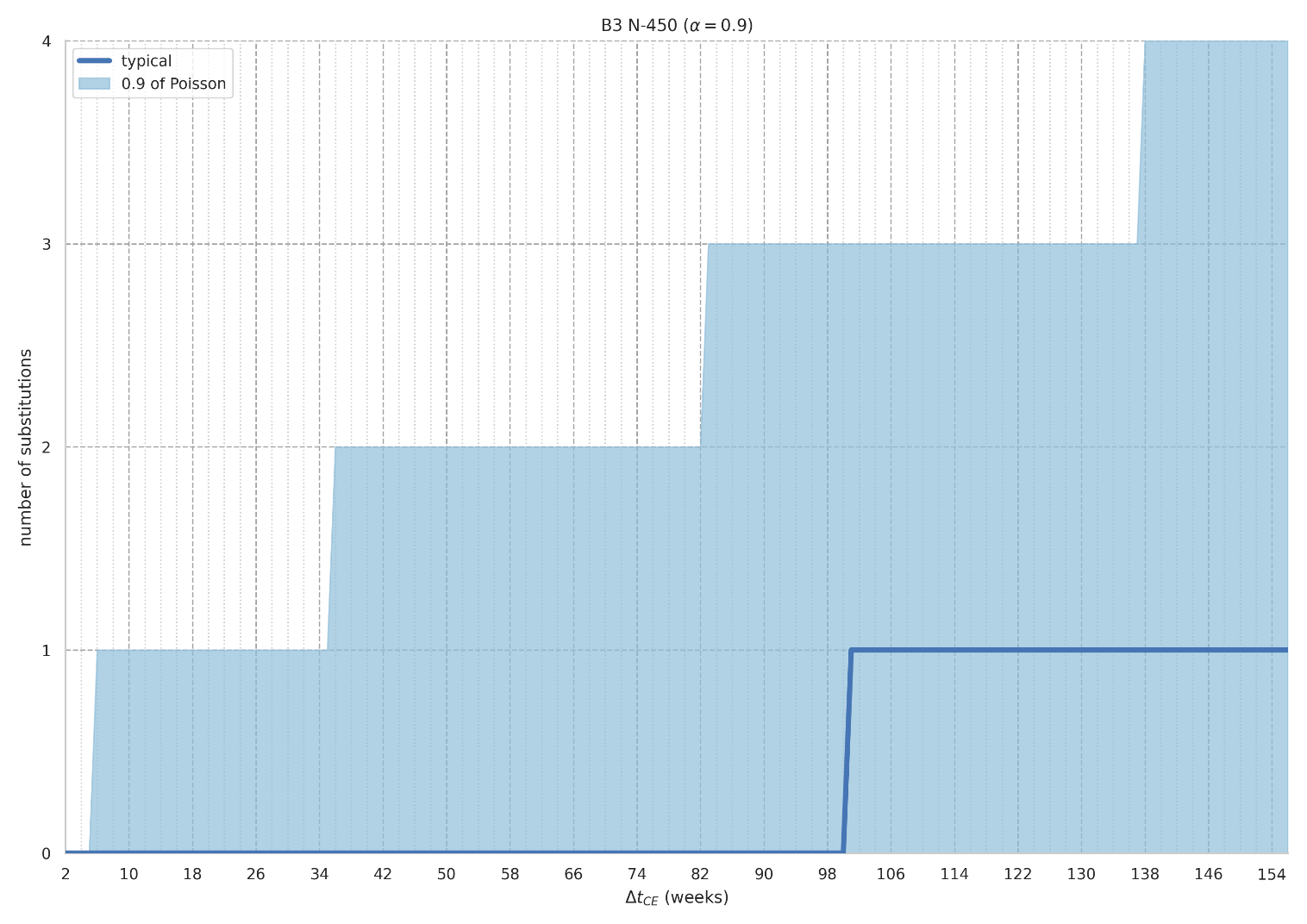

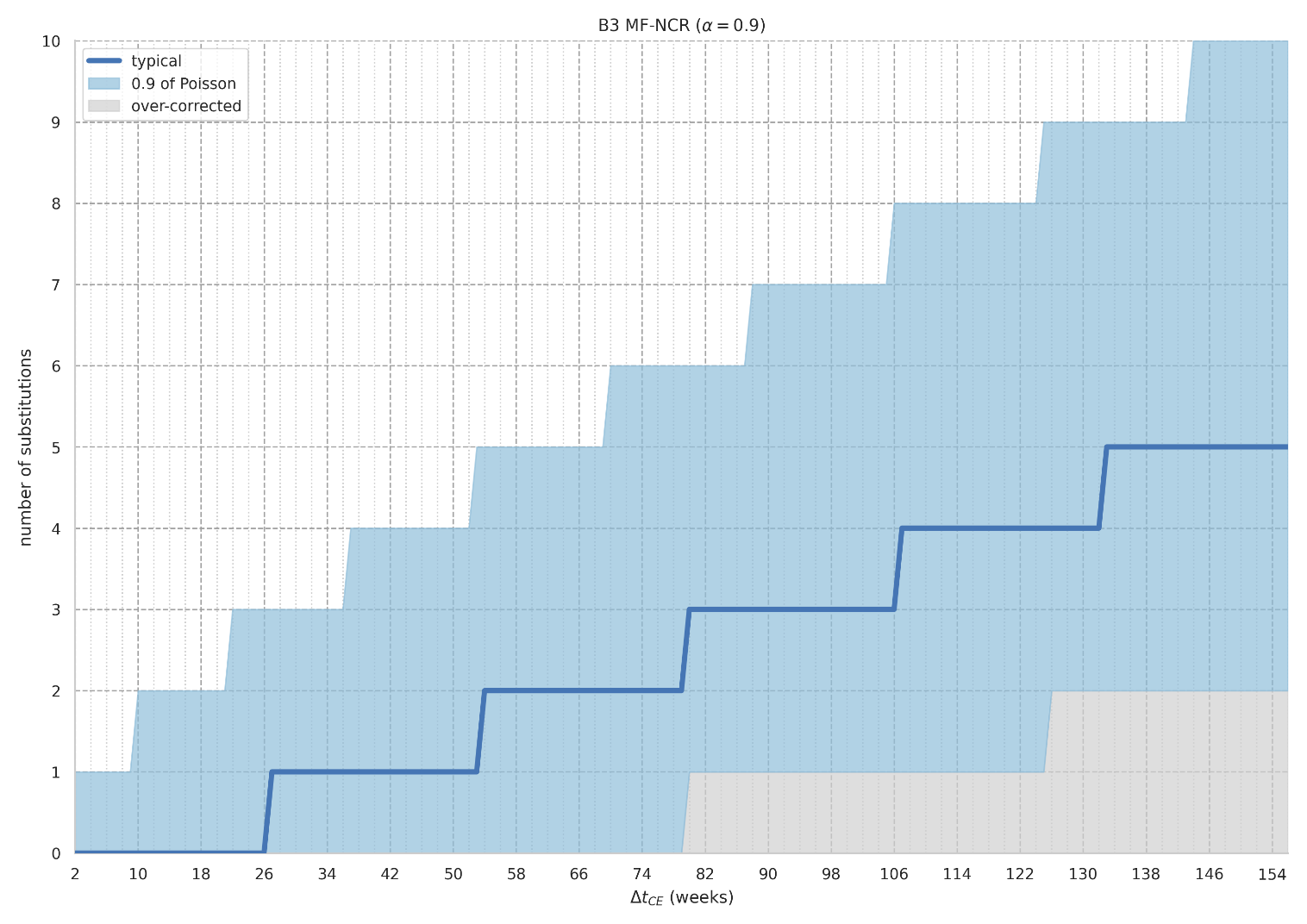

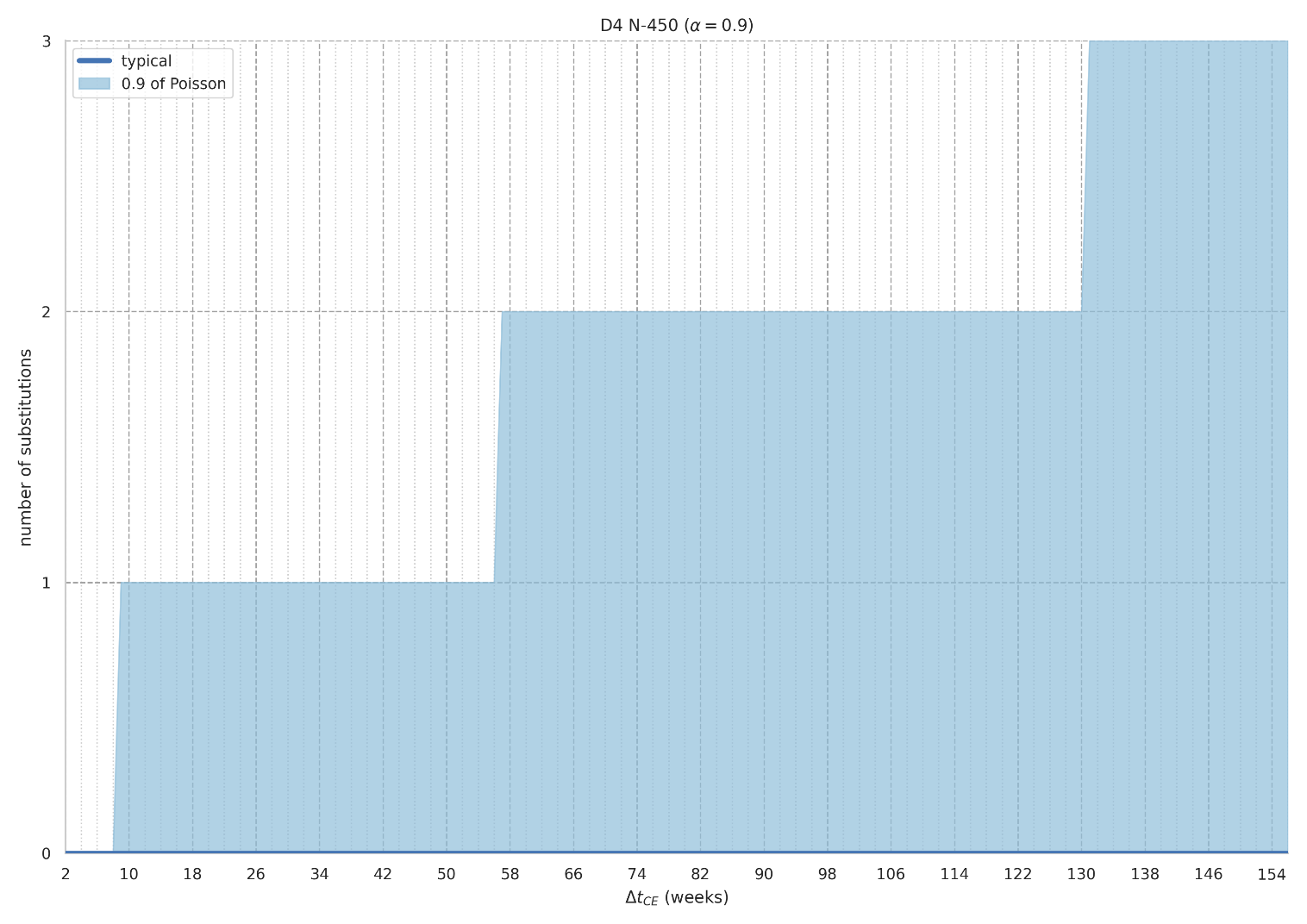

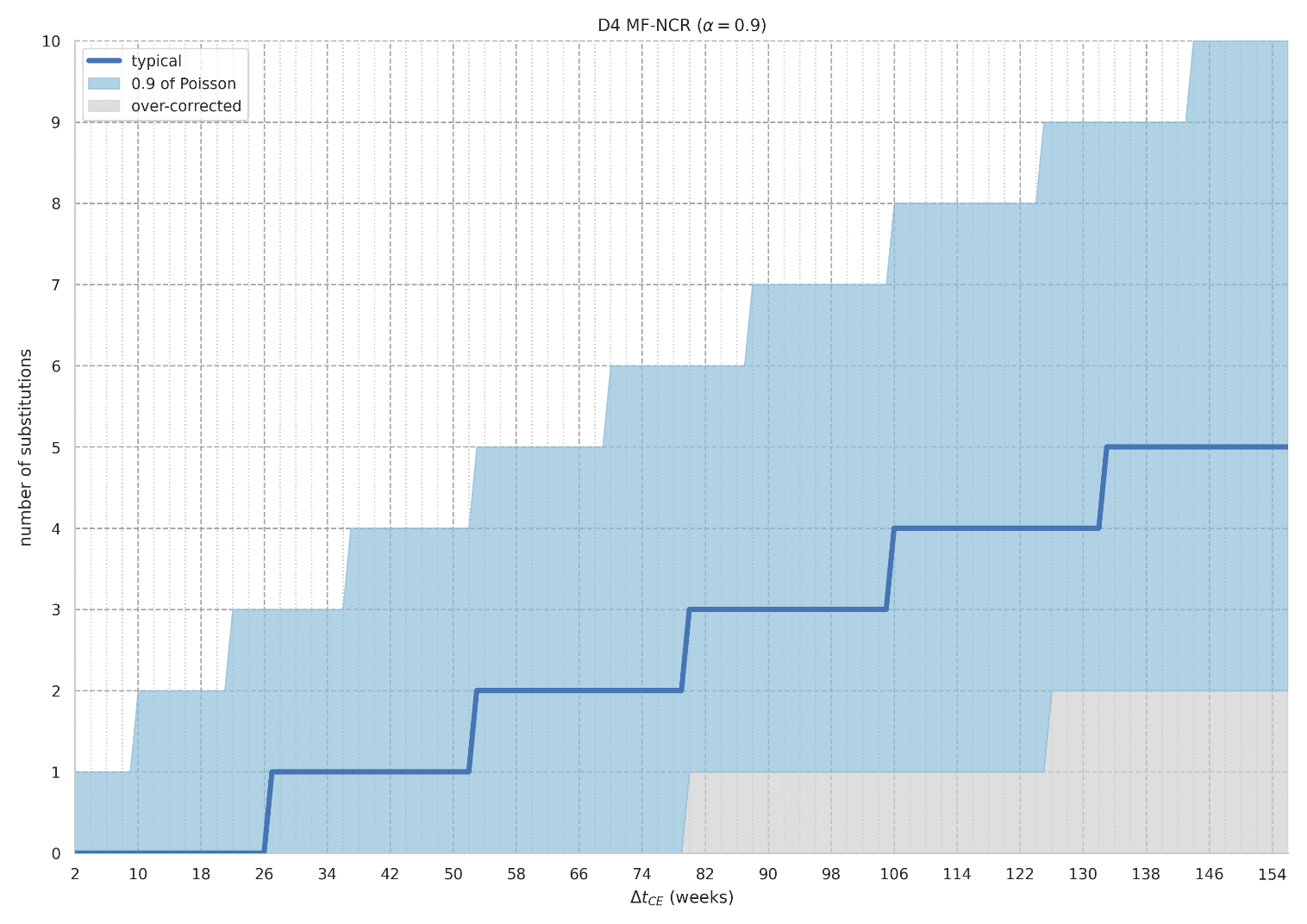

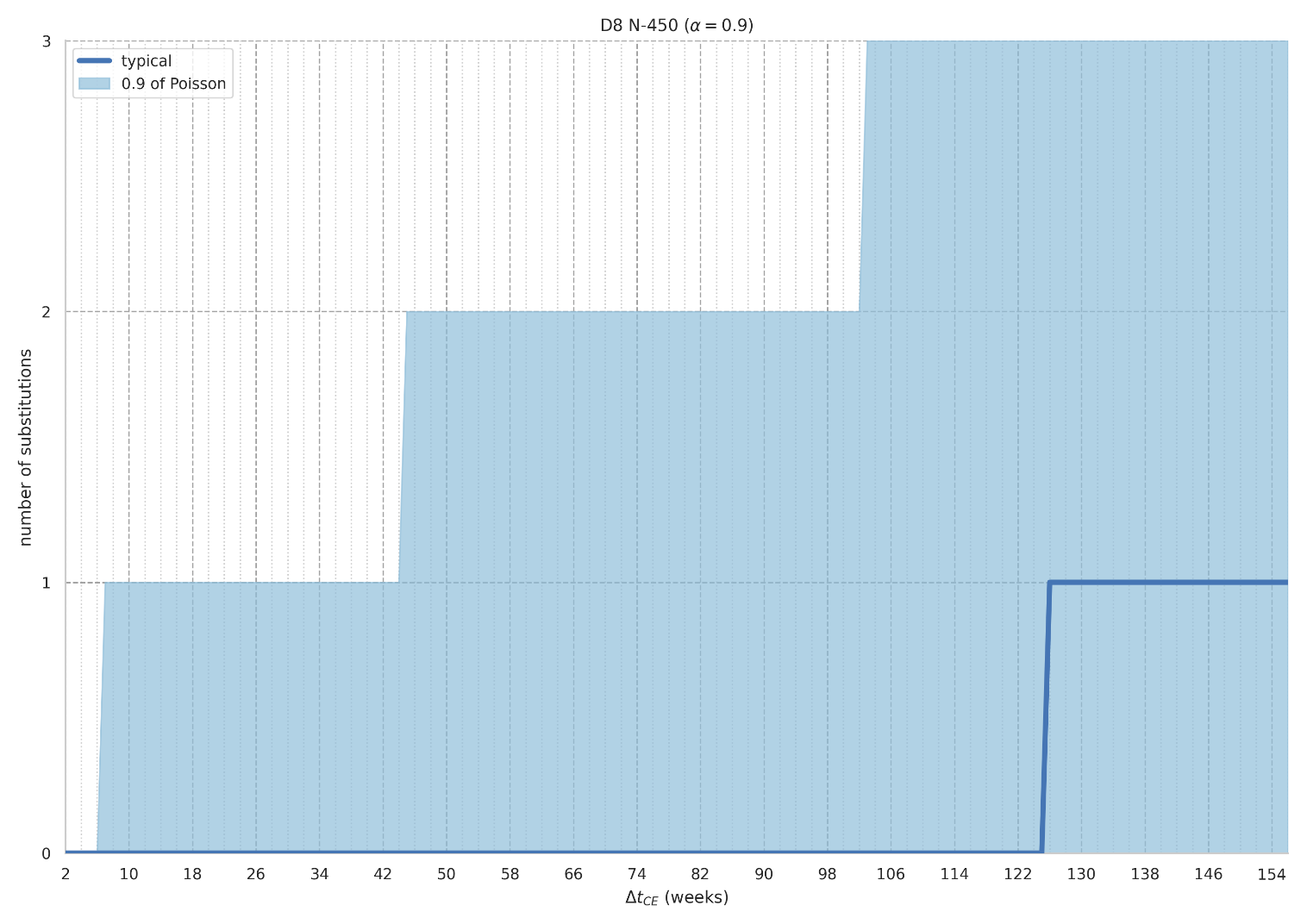

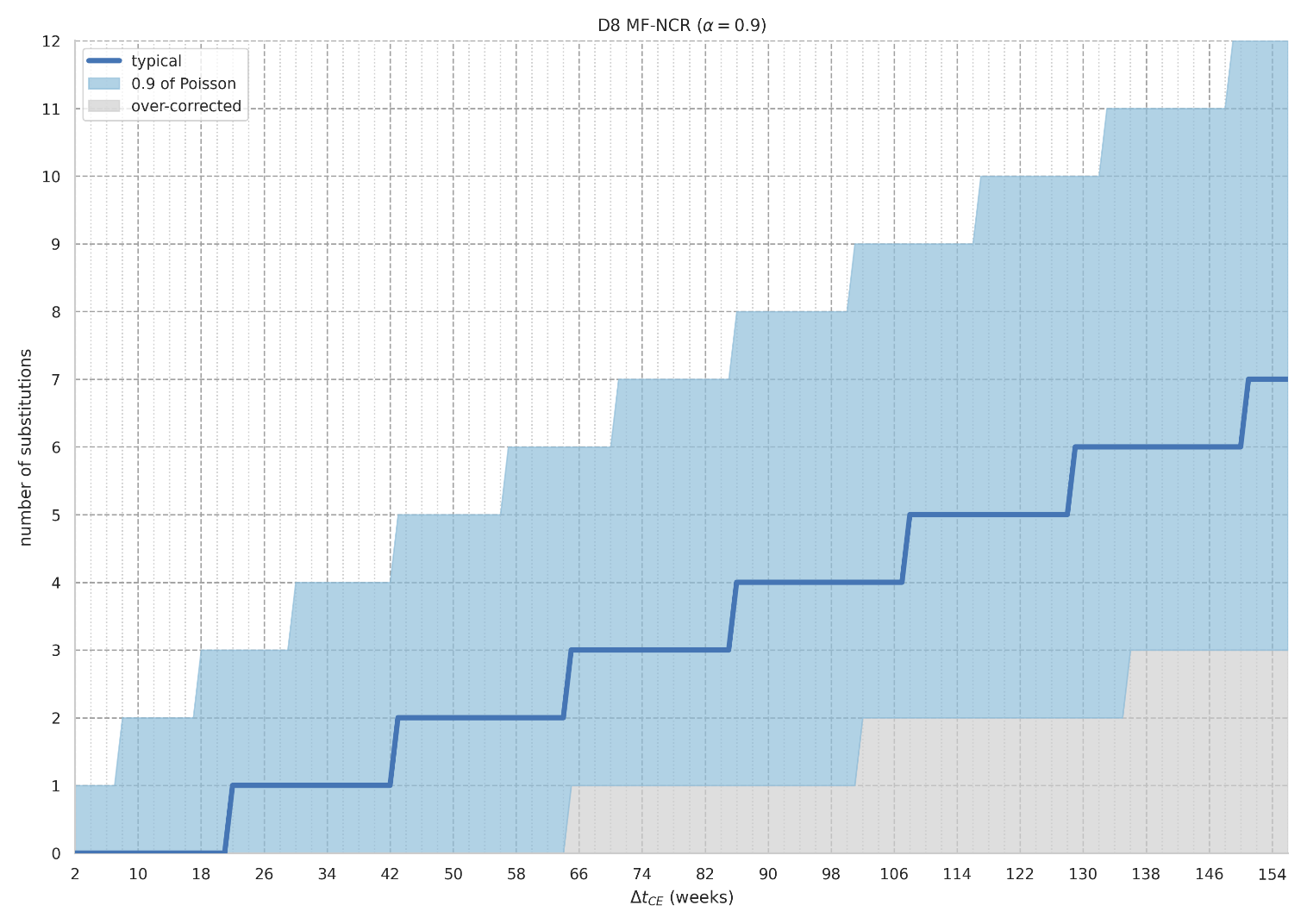


## 85% probability interval


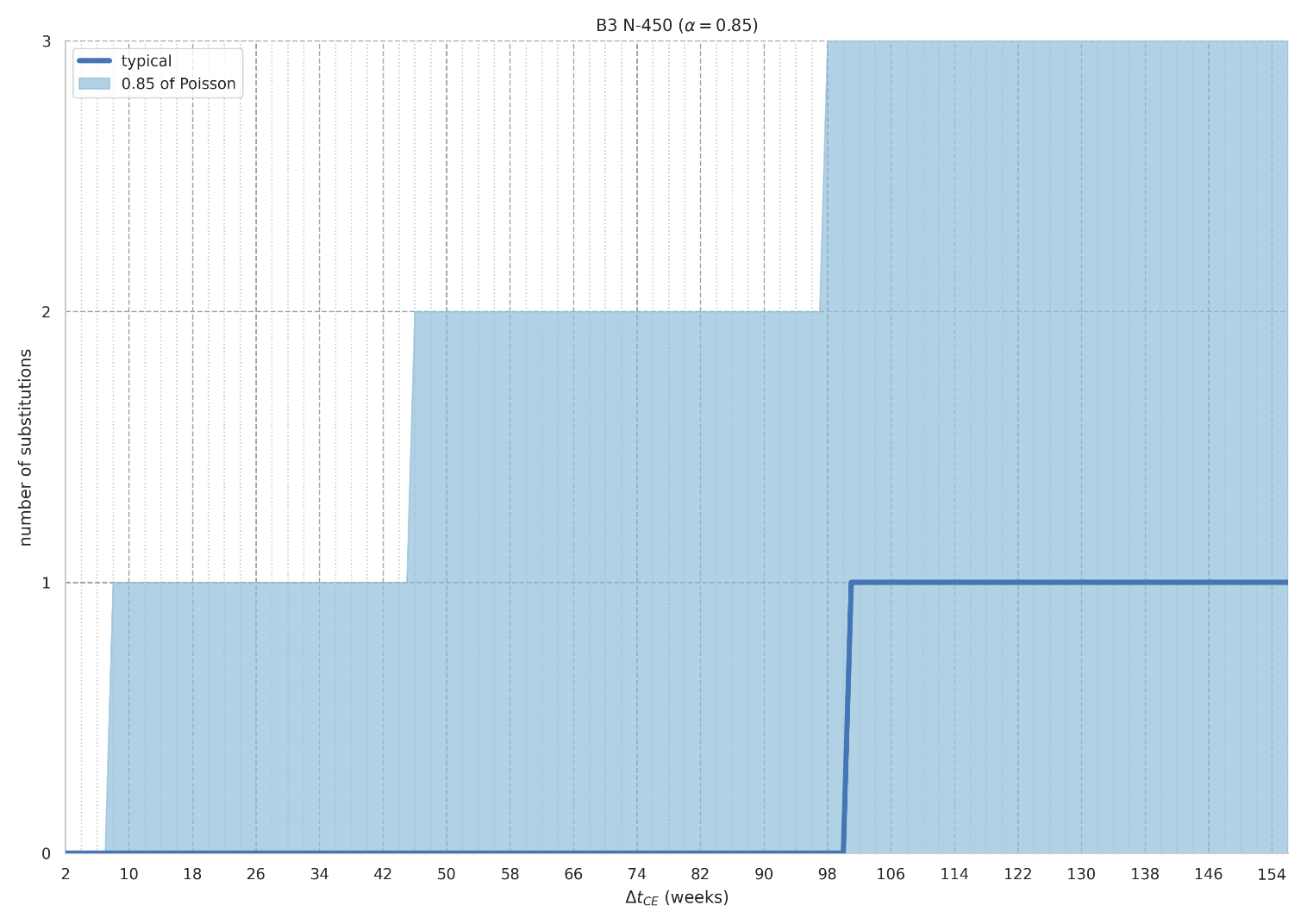

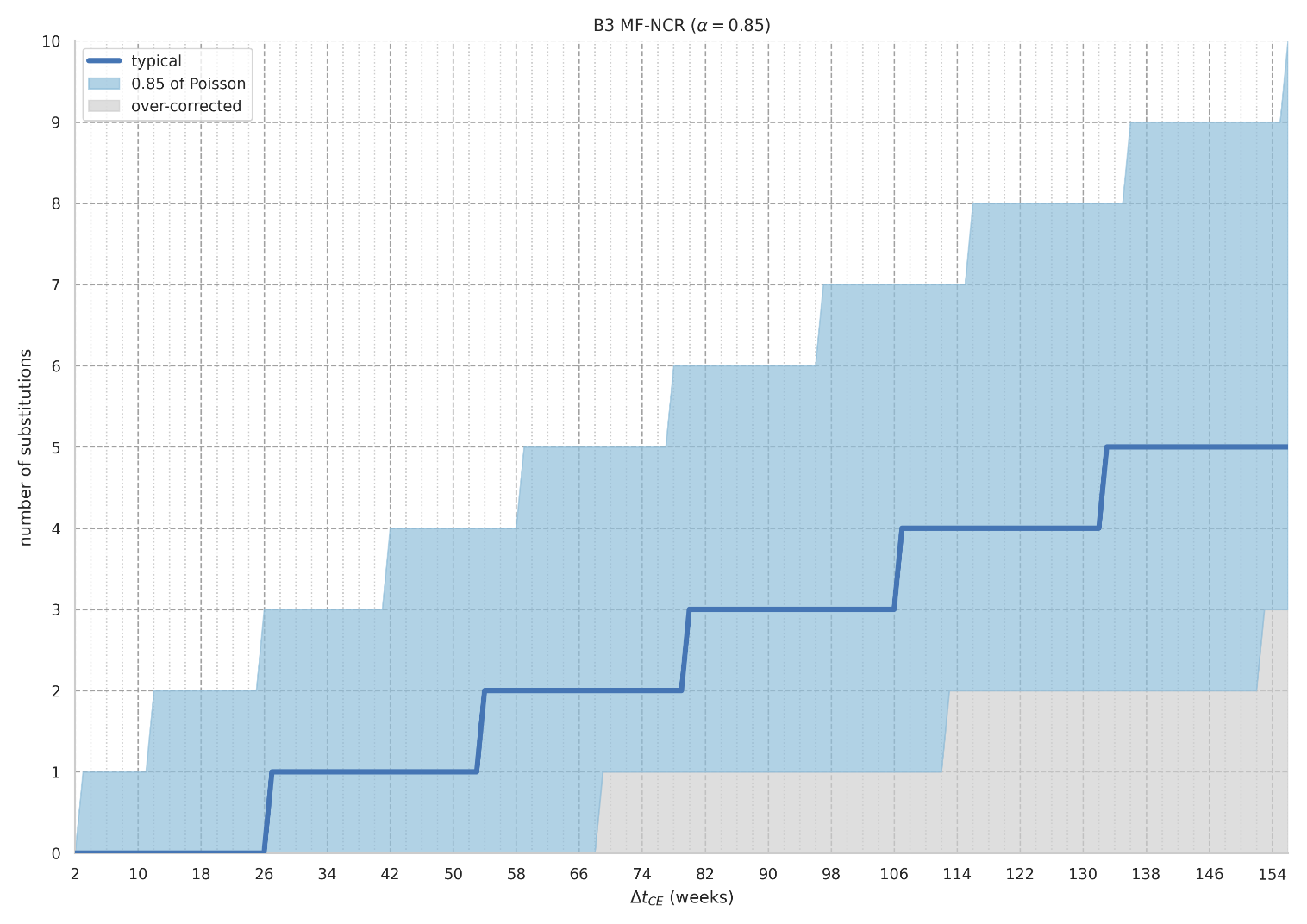

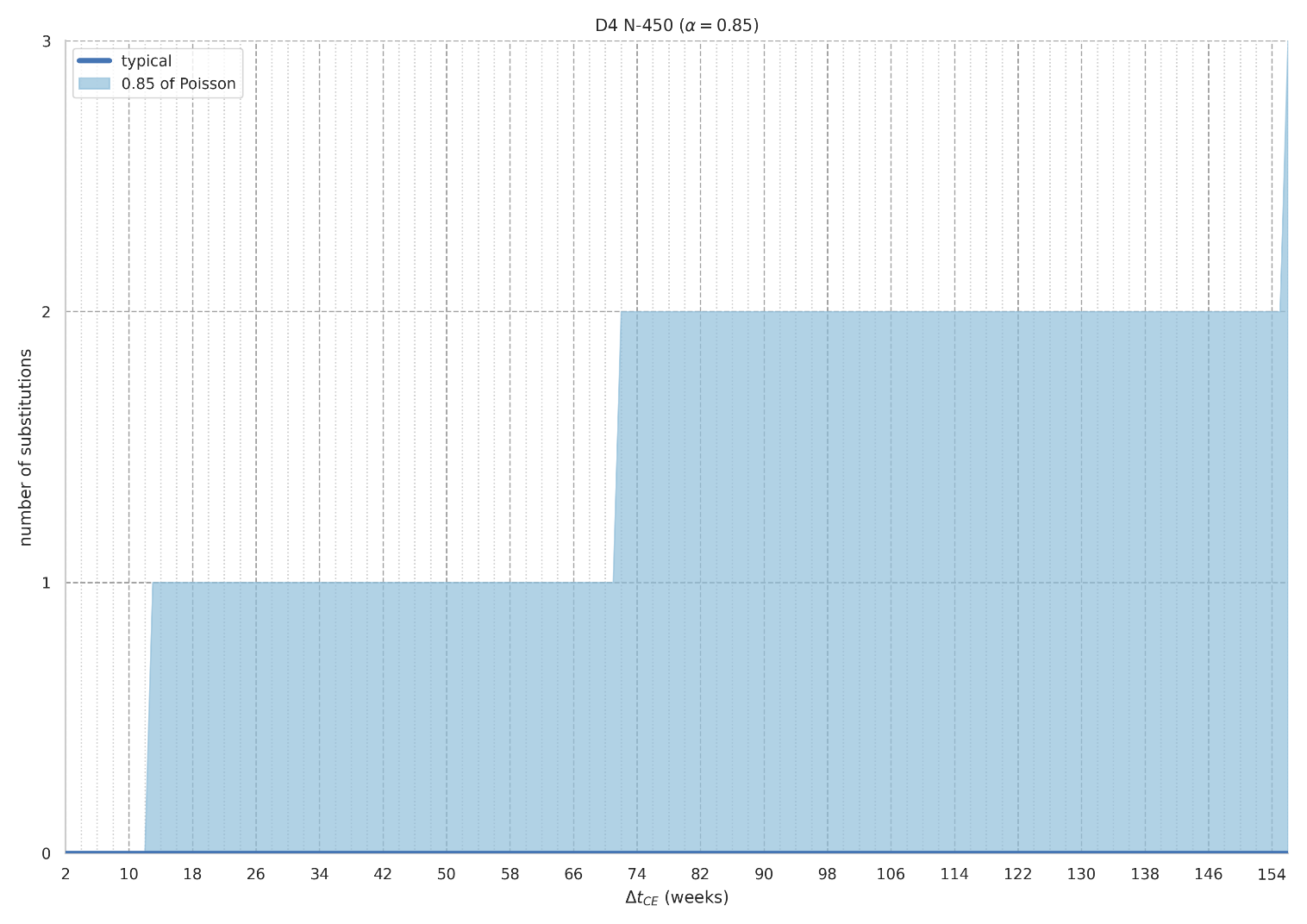

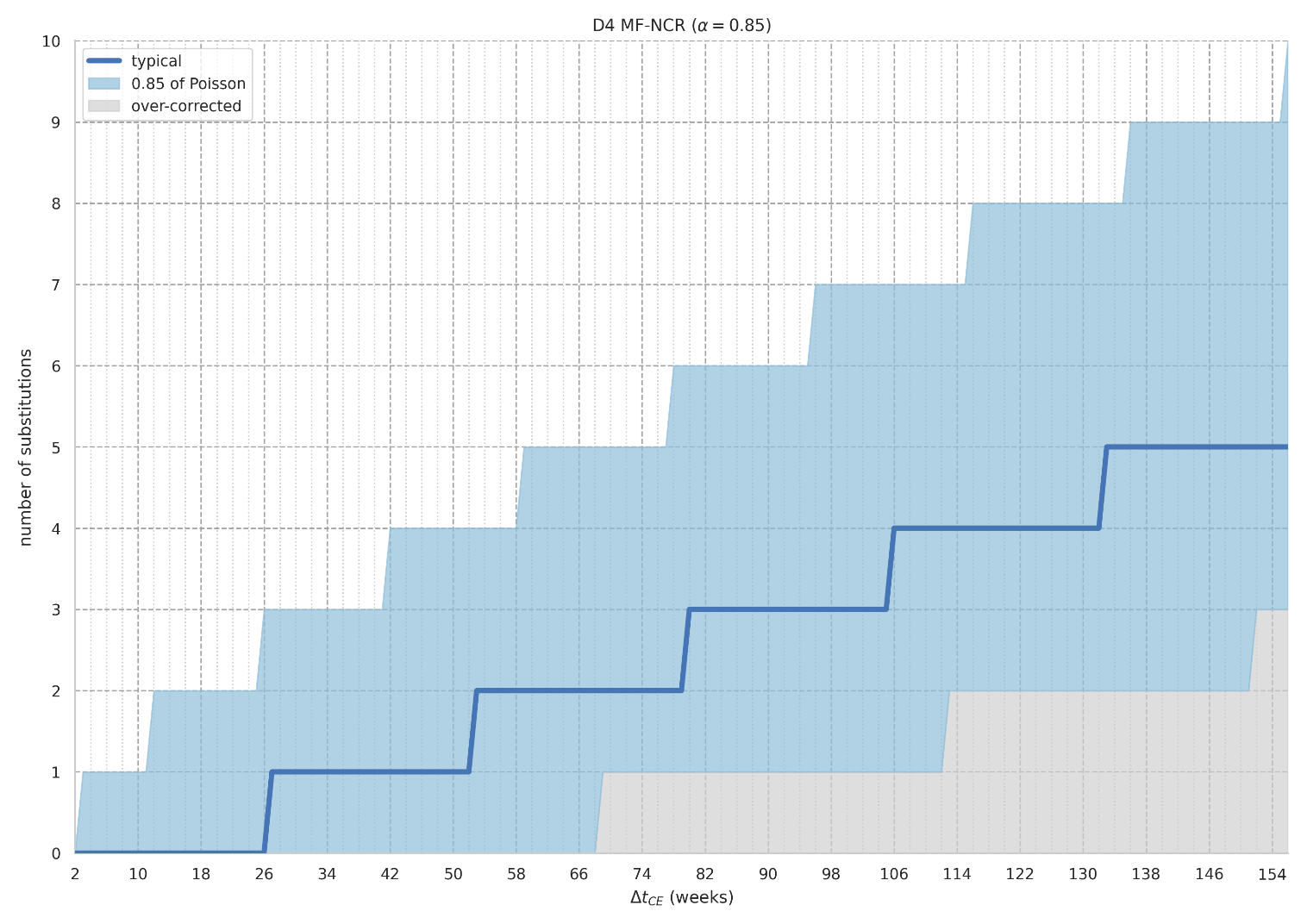

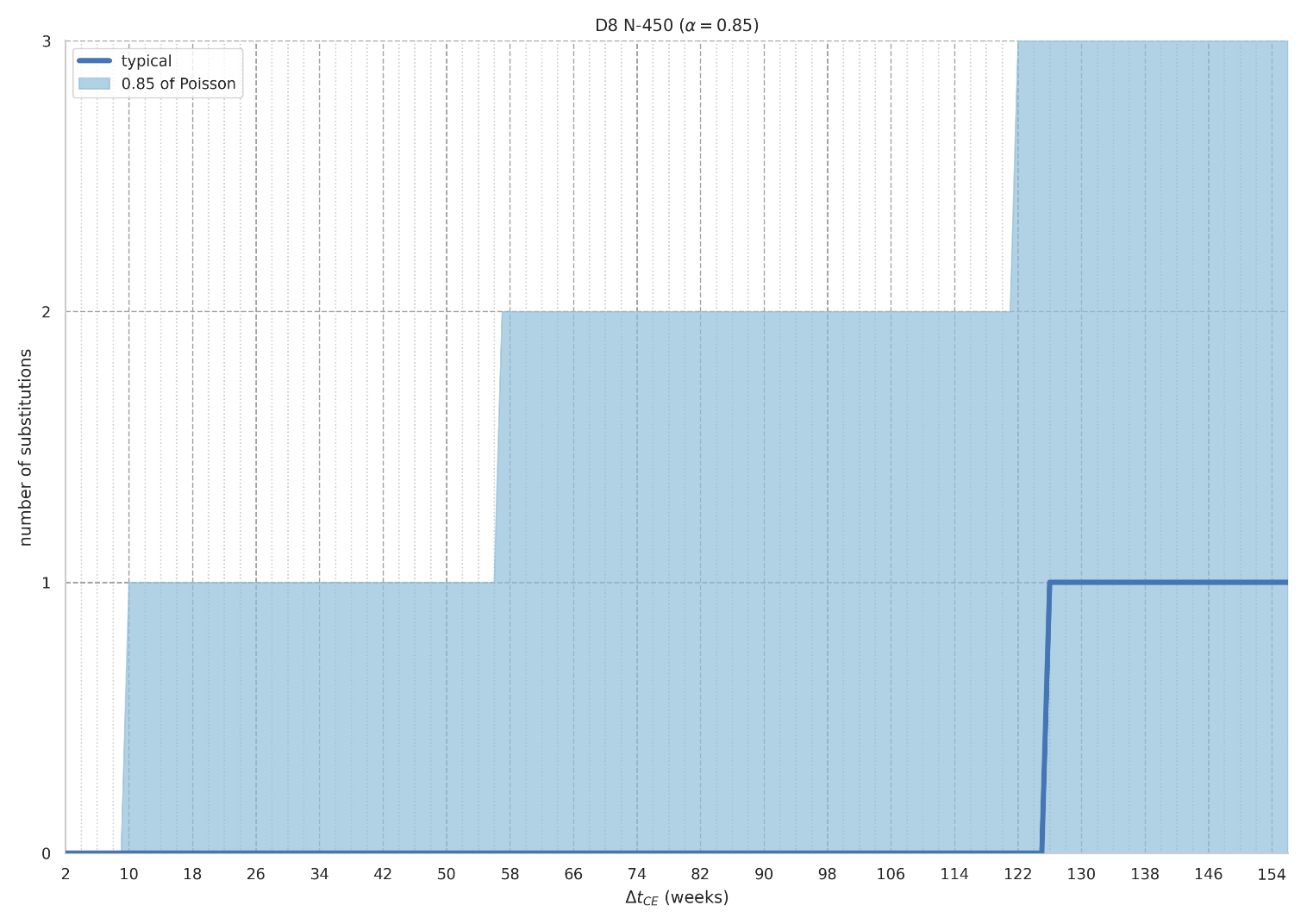

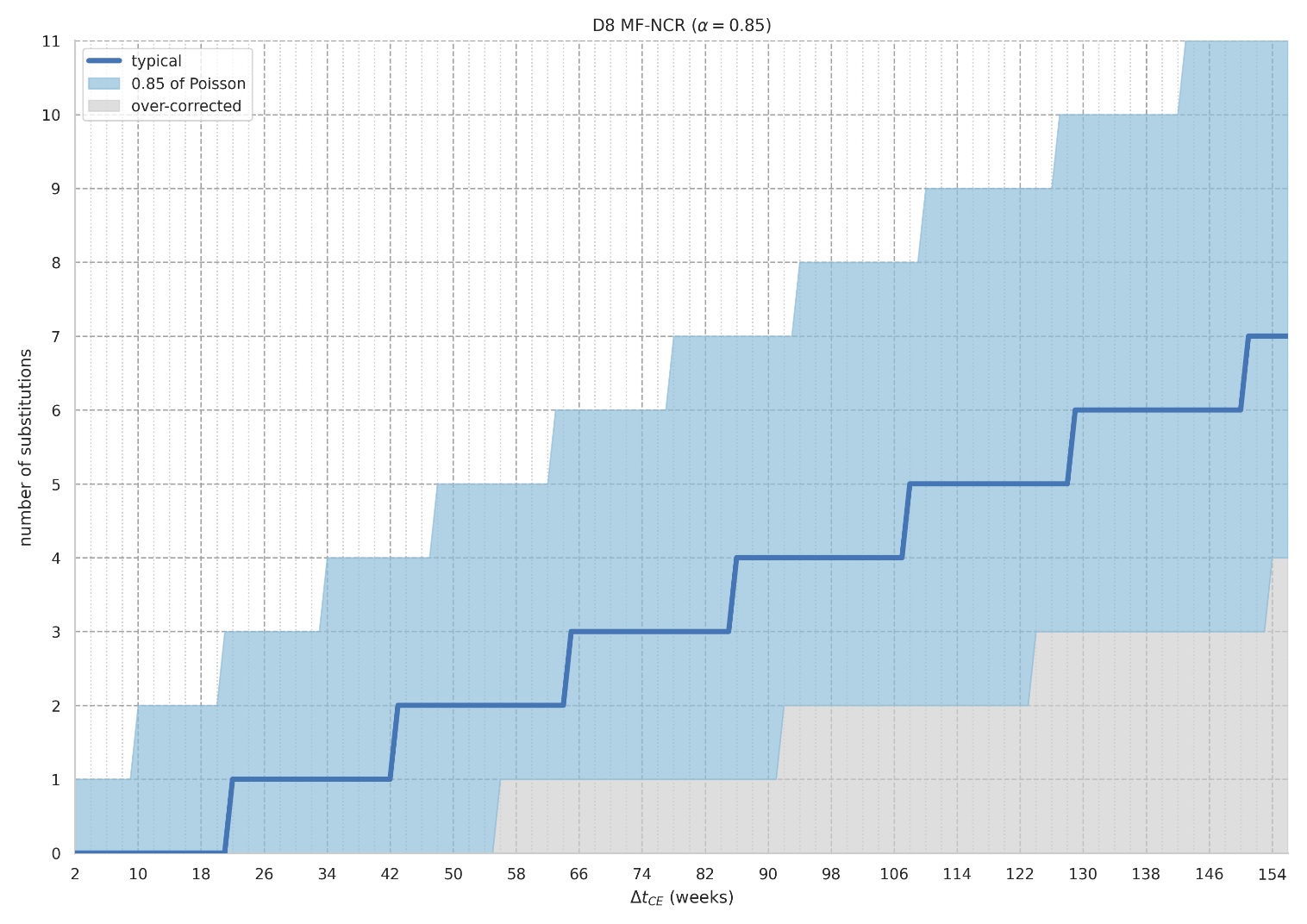


## 80% probability interval


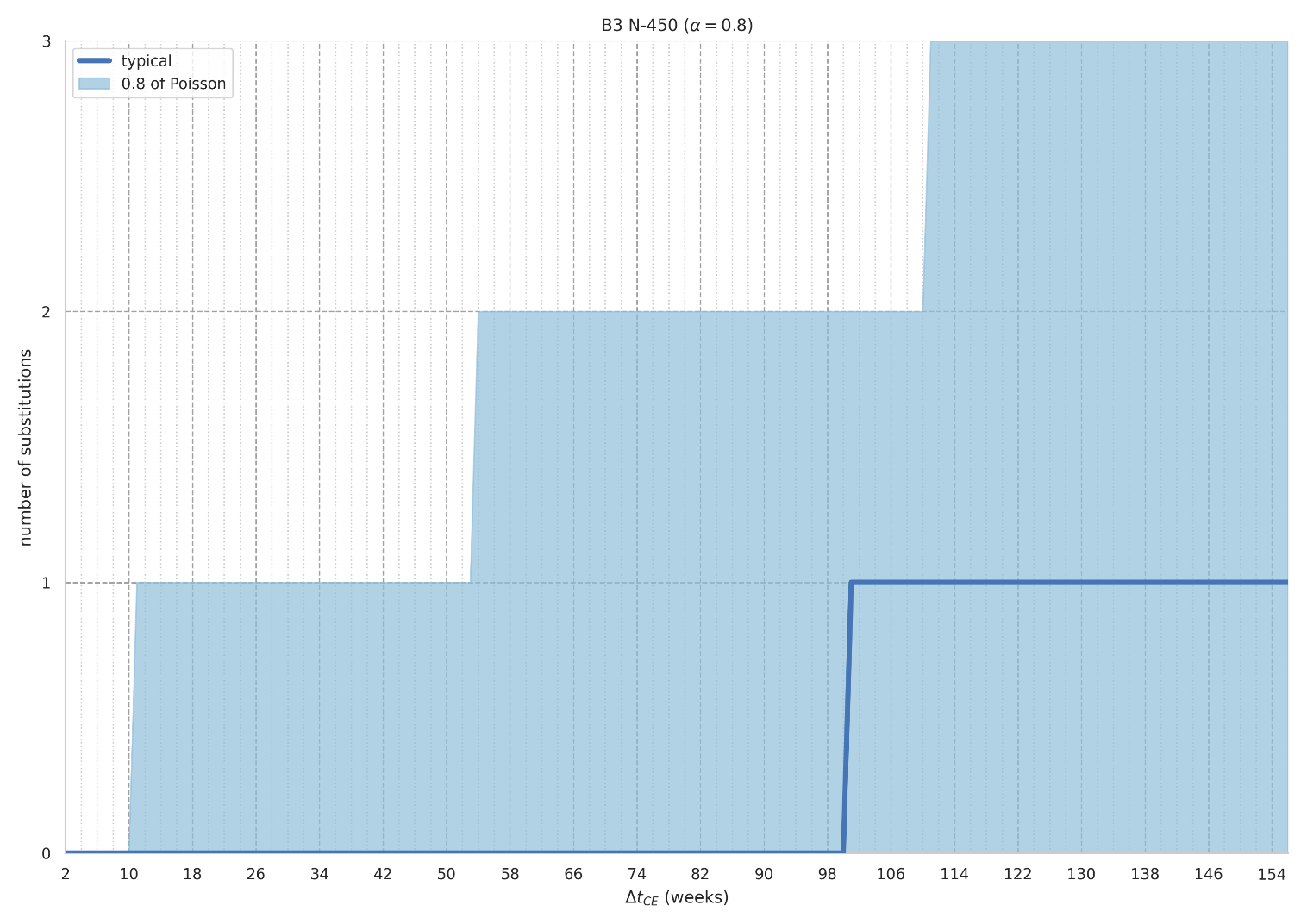

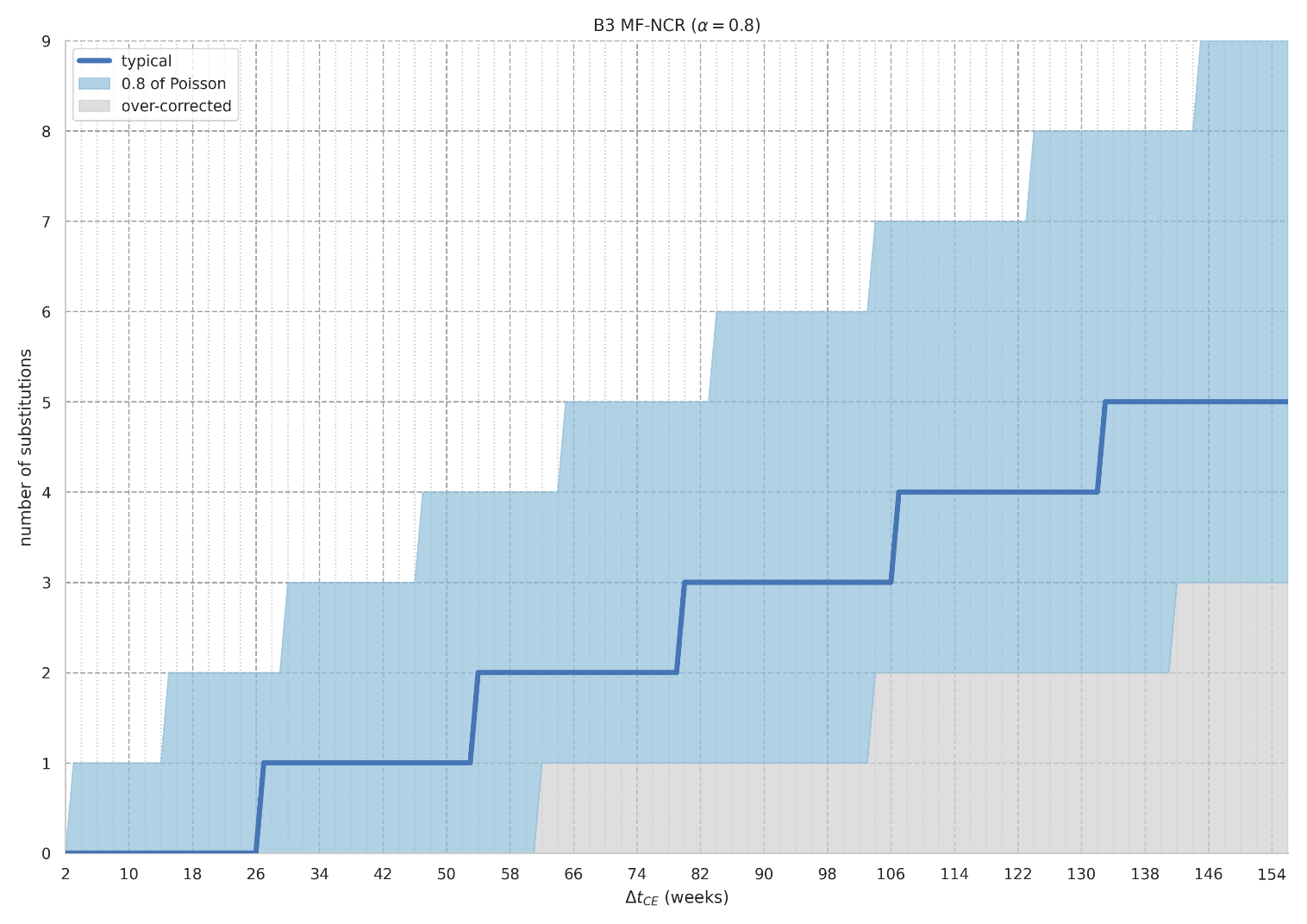


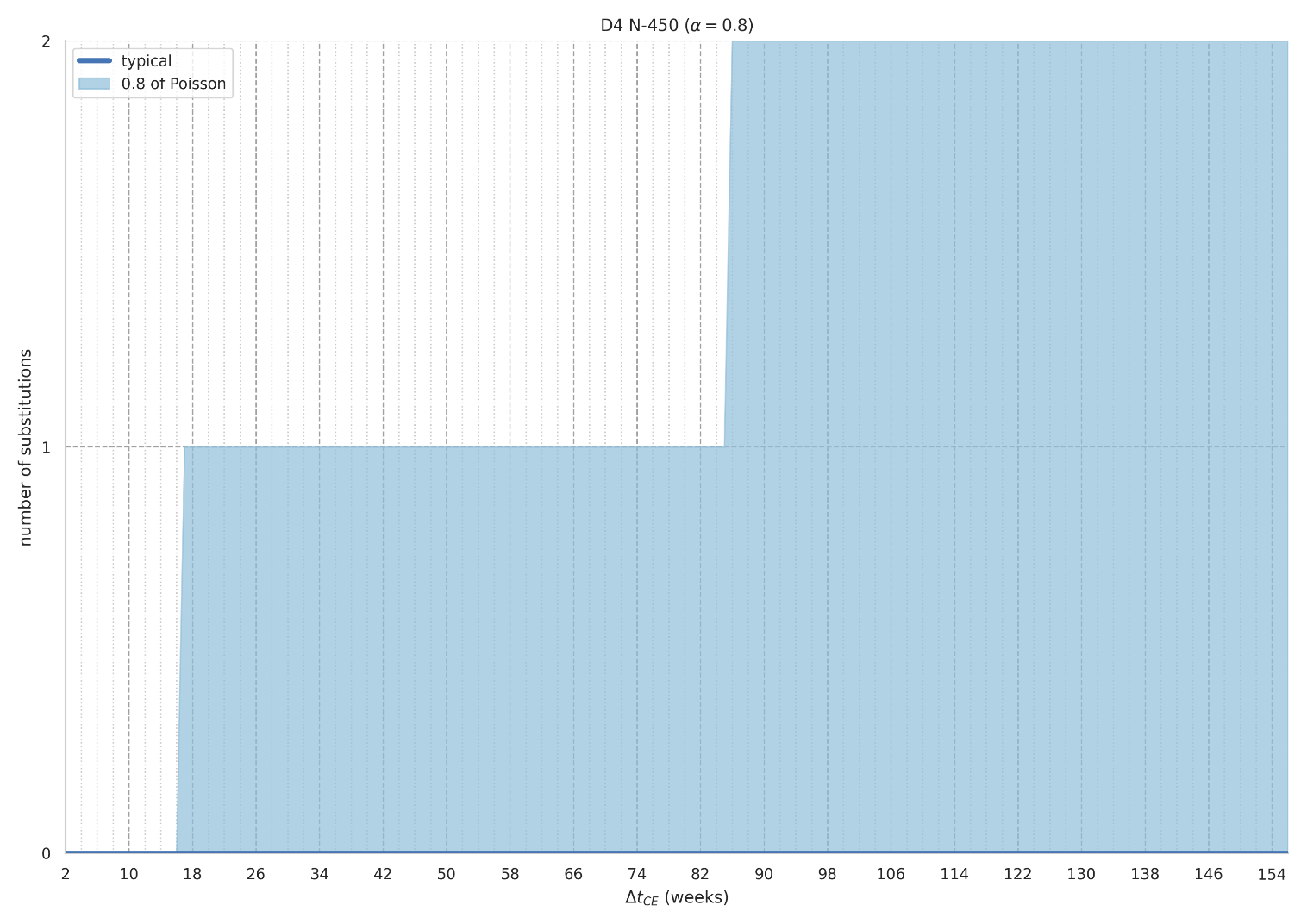

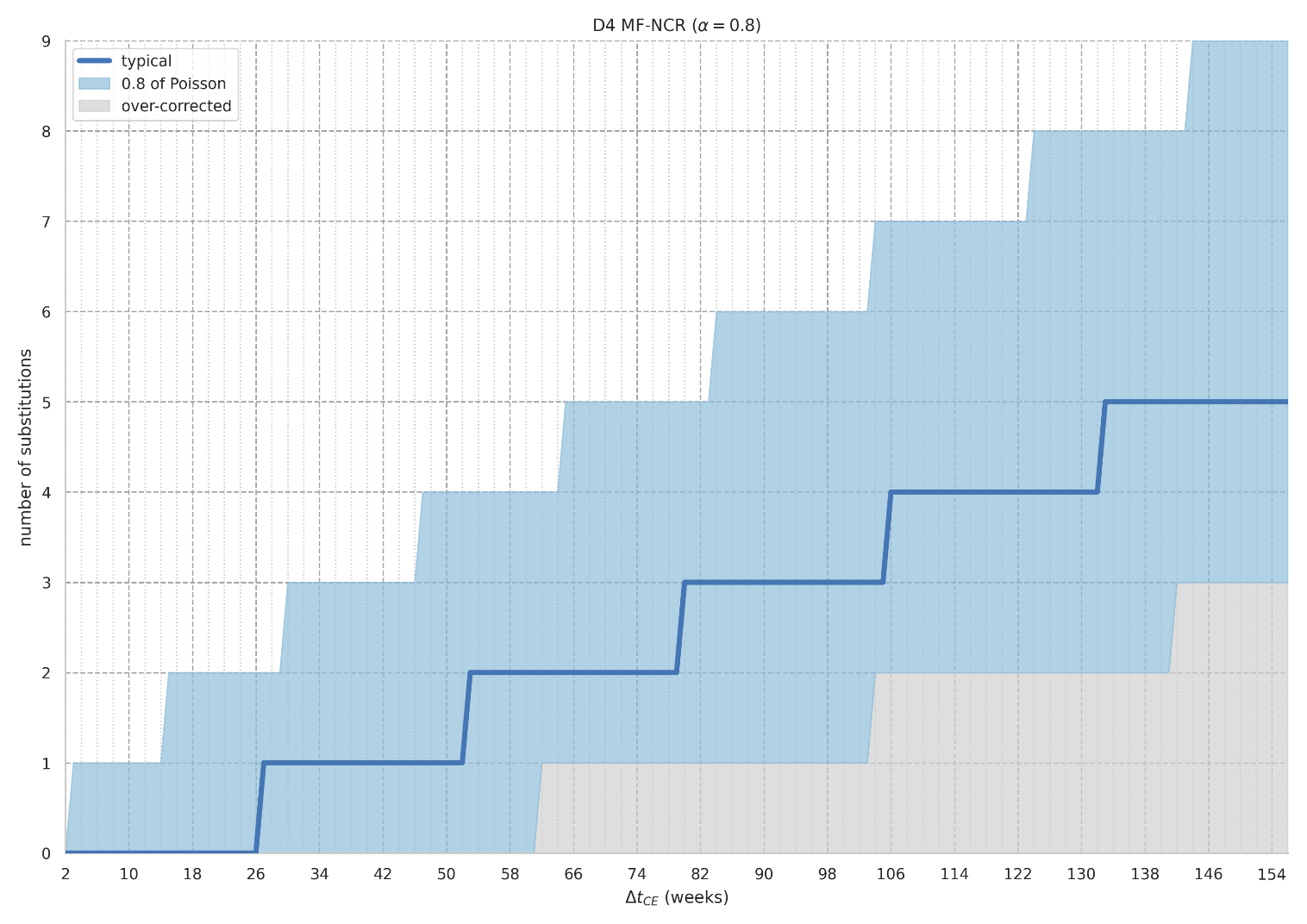

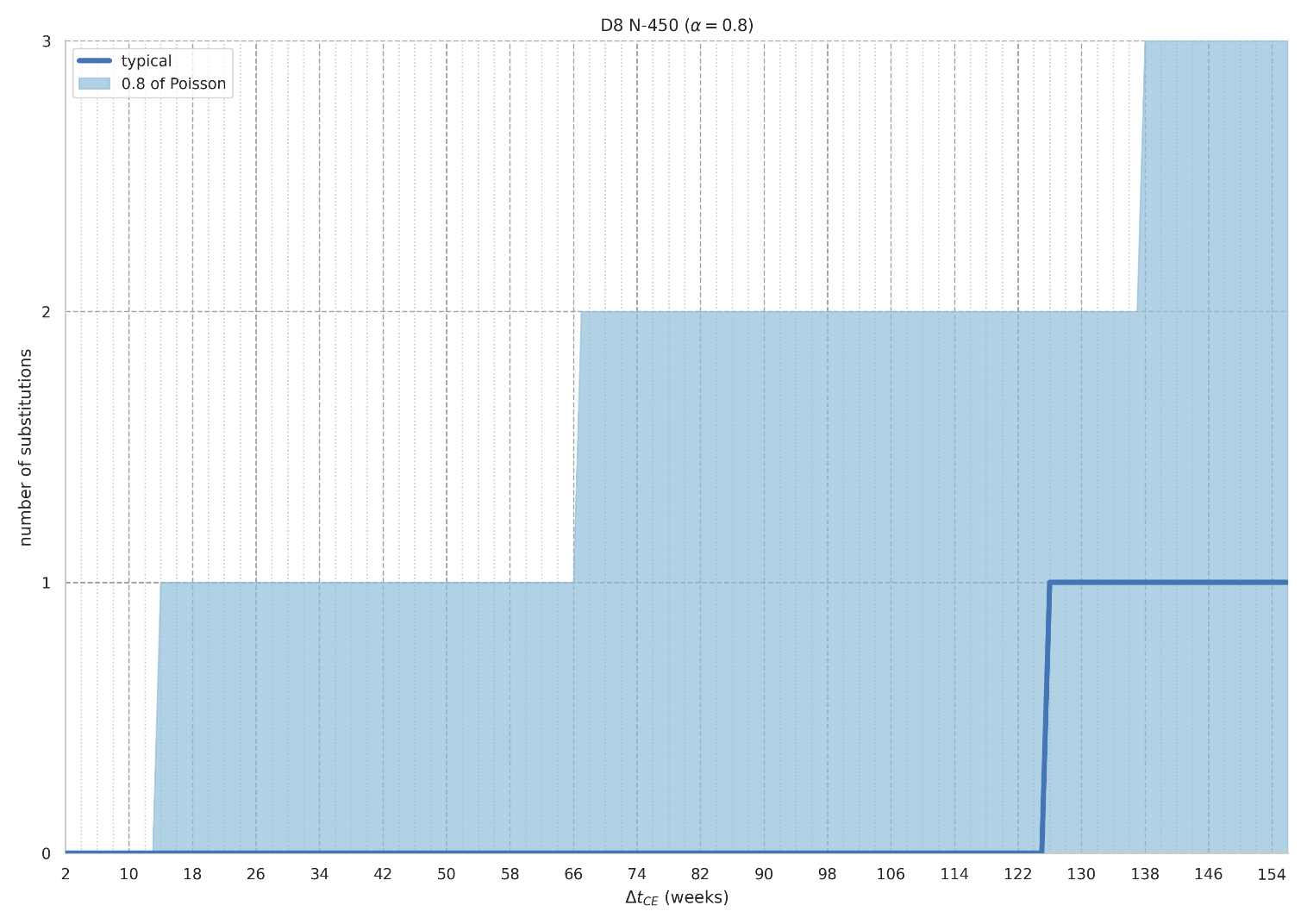

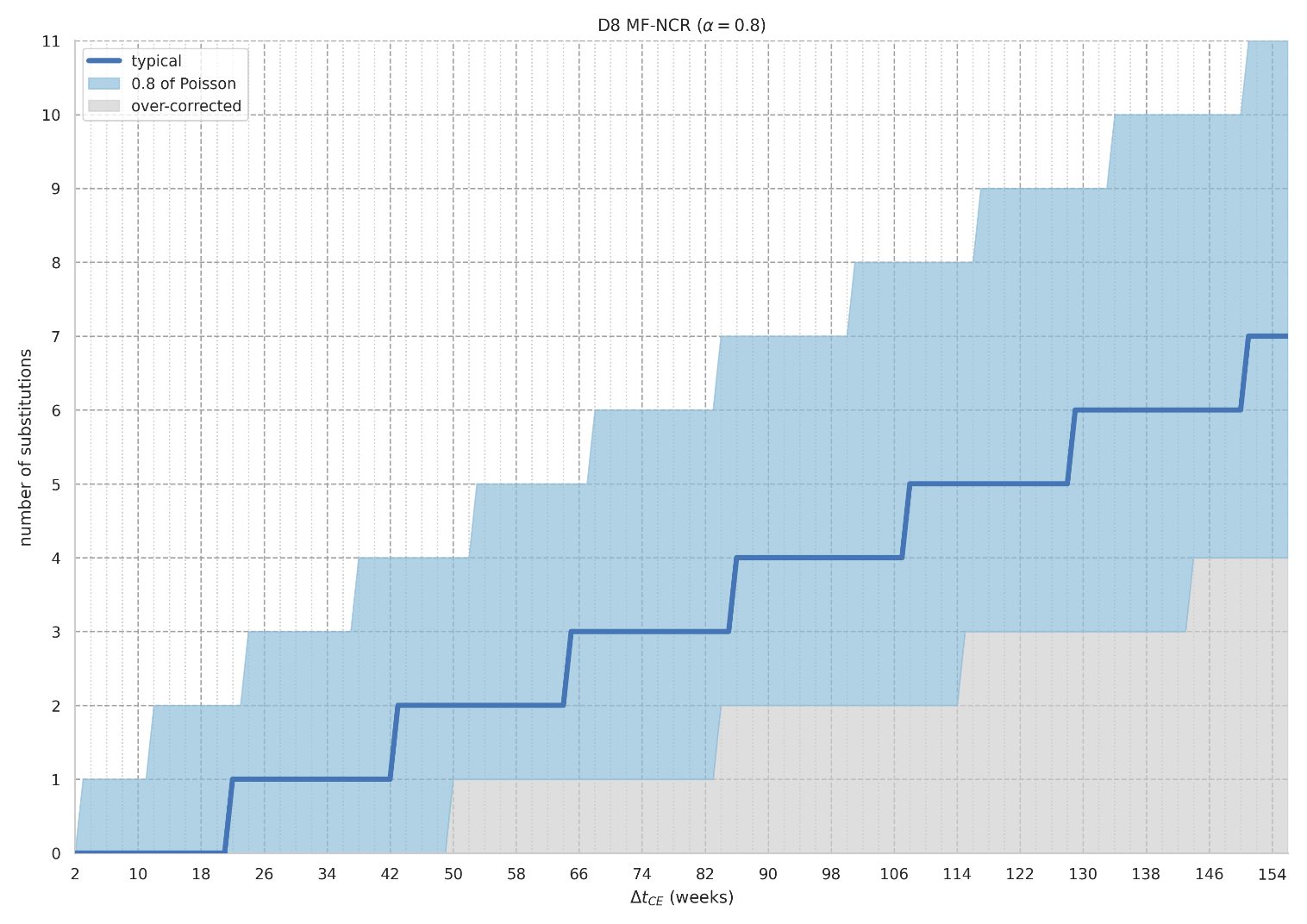

Supplement: Supplementary file 4 [file mmc4.docx]
