# Supplementary material for: Mind your Ps: A probabilistic model to aid the interpretation of molecular epidemiology data
Source: eBioMedicine. 2022 Apr 7;79:103989. doi: 10.1016/j.ebiom.2022.103989 (PMC9006250; doi:10.1016/j.ebiom.2022.103989)
Supplement: Supplementary file 6 [file mmc6.docx]

Supplement S6: Supplementary figures and tables

[Methods 1](#_Toc89015345)

[BEAST time-scaled phylogenetic trees 2](#_Toc89015346)

[Substitution rates 4](#_Toc89015347)

[Model statistics 5](#_Toc89015348)

[Verification datasets 6](#_Toc89015349)

[Validation datasets 8](#_Toc89015350)

# Methods


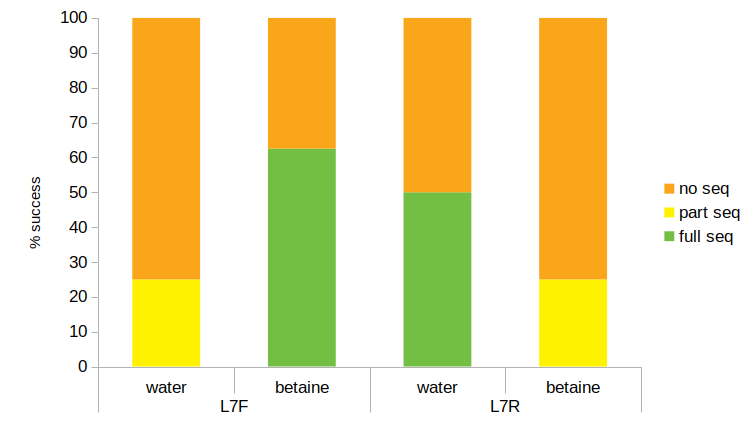


**Fig S6.1:** Sequencing success for 8 patient samples using the L7 amplicon primers diluted in either nuclease-free water or betaine.

# BEAST time-scaled phylogenetic trees

**
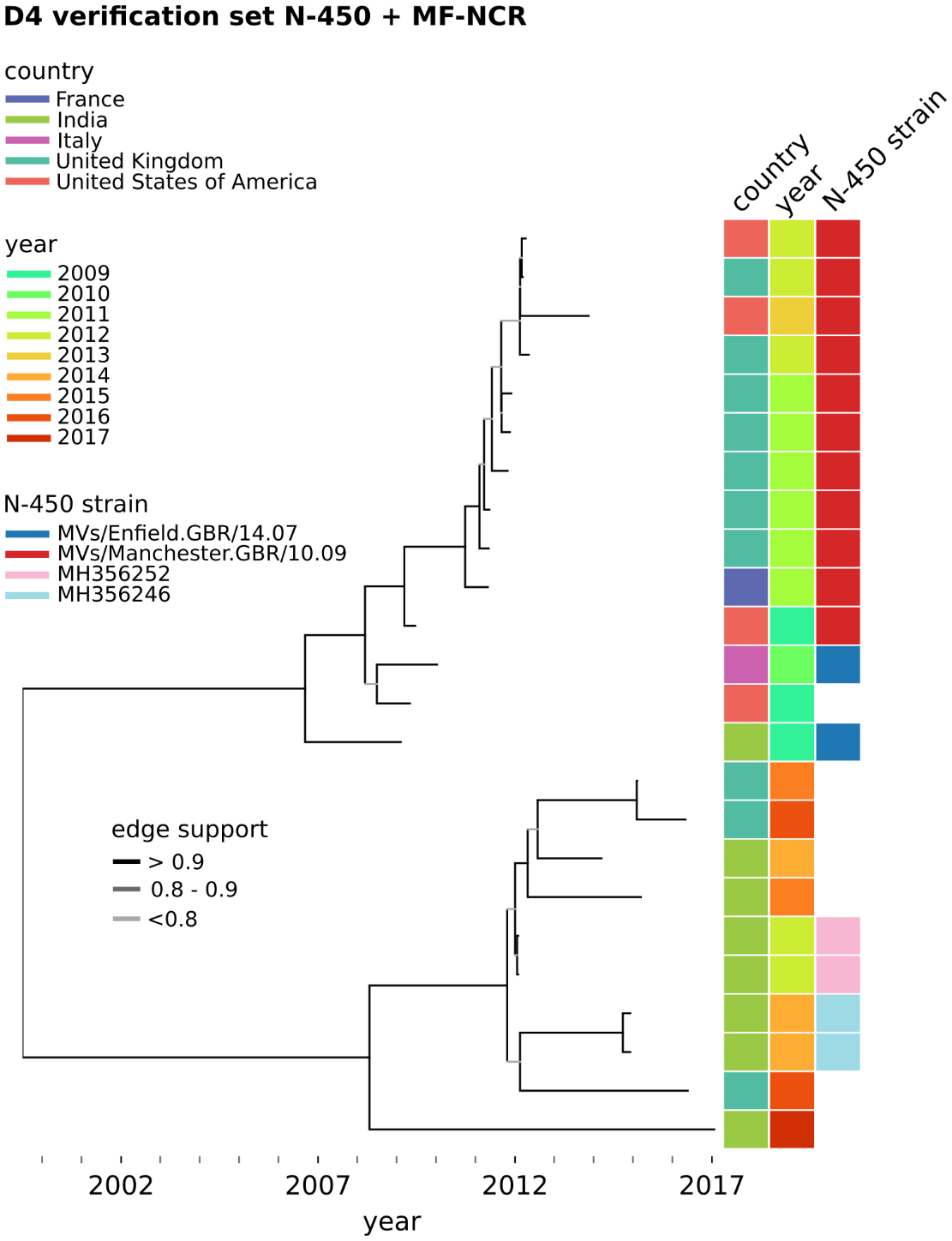
**

**Fig S6.2:** BEAST maximum credibility time-scaled phylogenetic tree for MeV genotype D4 samples in the verification set, used to obtain the substitution rates in Table S6.1. The N-450 and MF-NCR sequences were concatenated for the analysis. Tree tip labels are colour-coded according to country, sample year and the N-450 sequence (blank if no known matching named strain, or if no more than one sequence is found across datasets). Named strains are a WHO convention to identify widely circulating MeV strains with identical N-450 sequences.^18^ Samples with non-named N-450 sequences are labelled with the GenBank accession number for one of the sequences. Edge support values are given by the BEAST tree posterior values. N-450 named strain and DSID for all samples are included in Supplement S1.


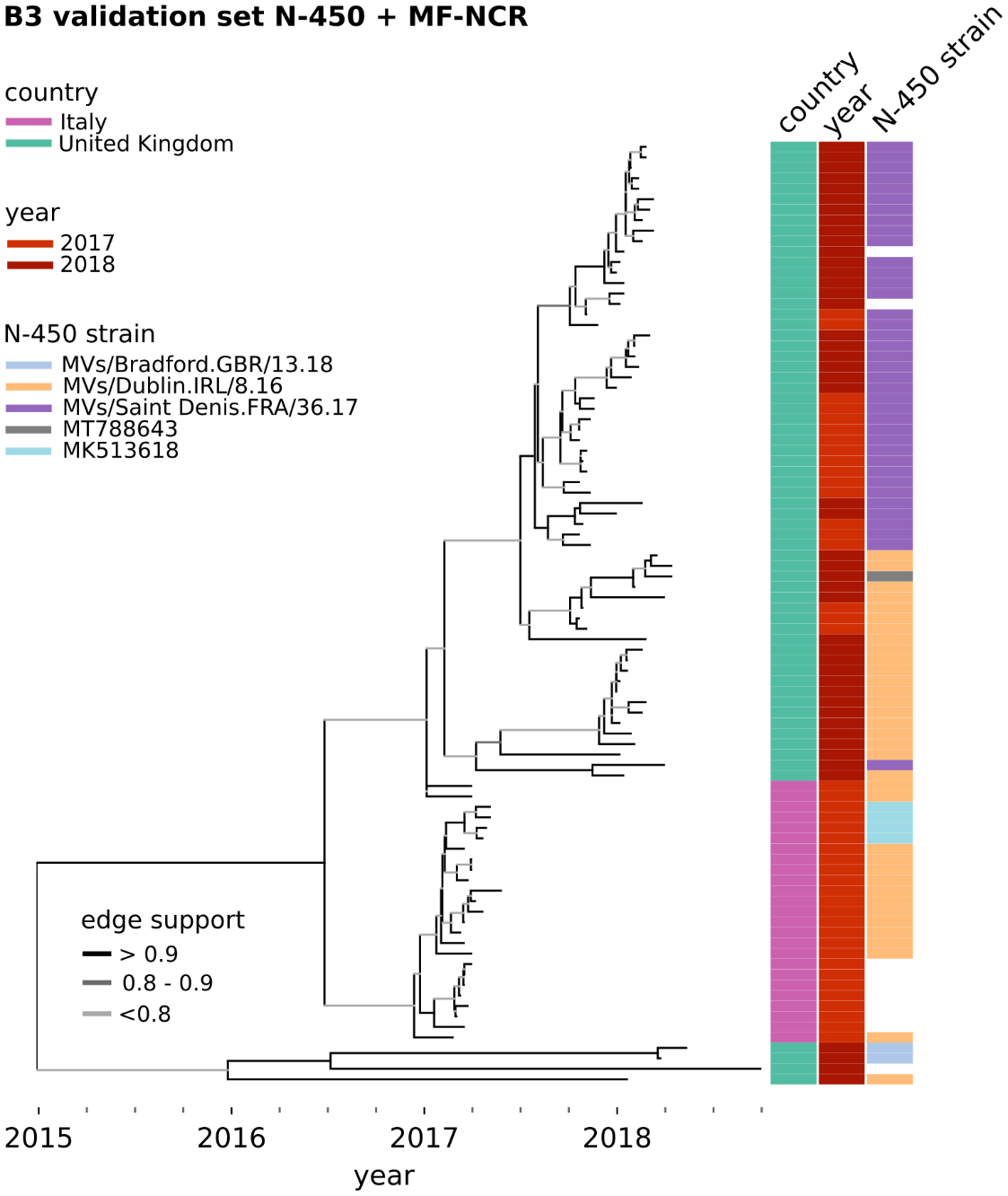


**Fig S6.3:** BEAST maximum credibility time-scaled phylogenetic tree for MeV genotype B3 samples used in the method validation. Please refer to Fig S6.2’s legend for details.

**
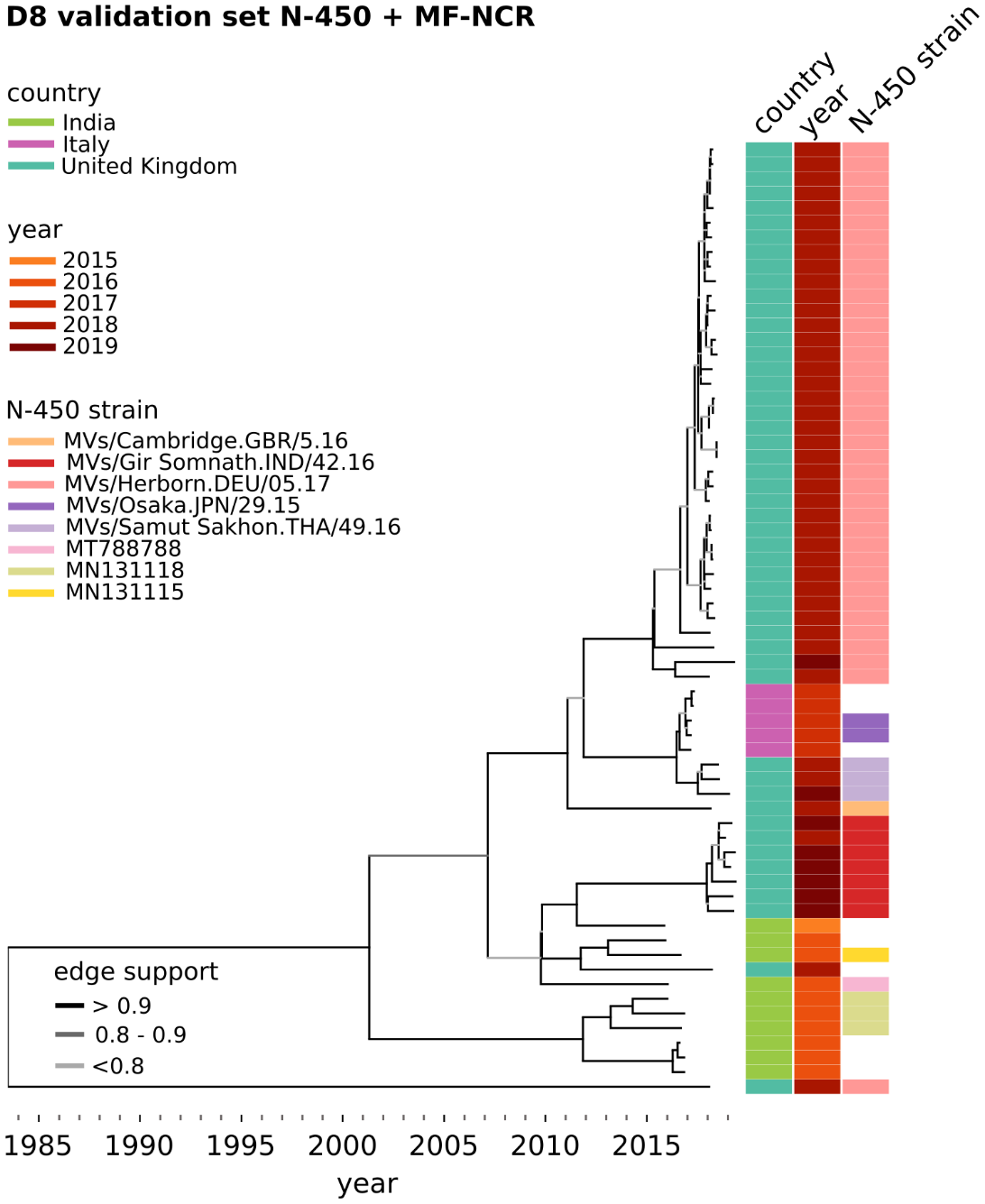
**

**Fig S6.4:** BEAST maximum credibility time-scaled phylogenetic tree for MeV genotype D8 samples used in the method validation. Please refer to Fig S6.2’s legend for details.

# Substitution rates

**Table S6.1:** Substitution rates in substitutions / (site.year) obtained from the BEAST analyses for all datasets. The intervals containing the 95% highest posterior intervals for the rate estimates based on the BEAST posterior distributions and the number of sites in the multiple sequence alignments used for the analyses are also listed.

| **Dataset** | **Genotype** | **Region** | **Substitution Rate** | **95% Highest Posterior Density** | | **Number of Sites^a^** |
| --- | --- | --- | --- | --- | --- | --- |
|  |  |  |  | **lower** | **upper** |  |
|  |  |  | **subs/(site.year)** | | |  |
| verification | B3 | N-450 | 1·15 x 10^-3^ | 7·62 x 10^-4^ | 1·60 x 10^-3^ | 450 |
|  |  | MF-NCR | 1·94 x 10^-3^ | 1·51 x 10^-3^ | 2·39 x 10^-3^ | 1012 |
|  | D4 | N-450 | 7·22 x 10^-4^ | 3·55 x 10^-4^ | 1·15 x 10^-3^ | 450 |
|  |  | MF-NCR | 1·93 x 10^-3^ | 1·28 x 10^-3^ | 2·60 x 10^-3^ | 1019 |
|  | D8 | N-450 | 9·23 x 10^-4^ | 6·53 x 10^-4^ | 1·20 x 10^-3^ | 450 |
|  |  | MF-NCR | 2·39 x 10^-3^ | 1·95 x 10^-3^ | 2·82 x 10^-3^ | 1012 |
| validation | B3 | N-450 | 9·98 x 10^-4^ | 3·49 x 10^-4^ | 1·77 x 10^-3^ | 450 |
|  |  | MF-NCR | 4·44 x 10^-3^ | 2·44 x 10^-3^ | 6·40 x 10^-3^ | 1012 |
|  | D8 | N-450 | 6·24 x 10^-4^ | 3·02 x 10^-4^ | 9·86 x 10^-4^ | 450 |
|  |  | MF-NCR | 1·99 x 10^-3^ | 1·06 x 10^-3^ | 2·89 x 10^-3^ | 1012 |

^a^ Number of sites in the multiple sequence alignment. Some D4 strains’ MF-NCR sequences contain insertions and deletions.

 
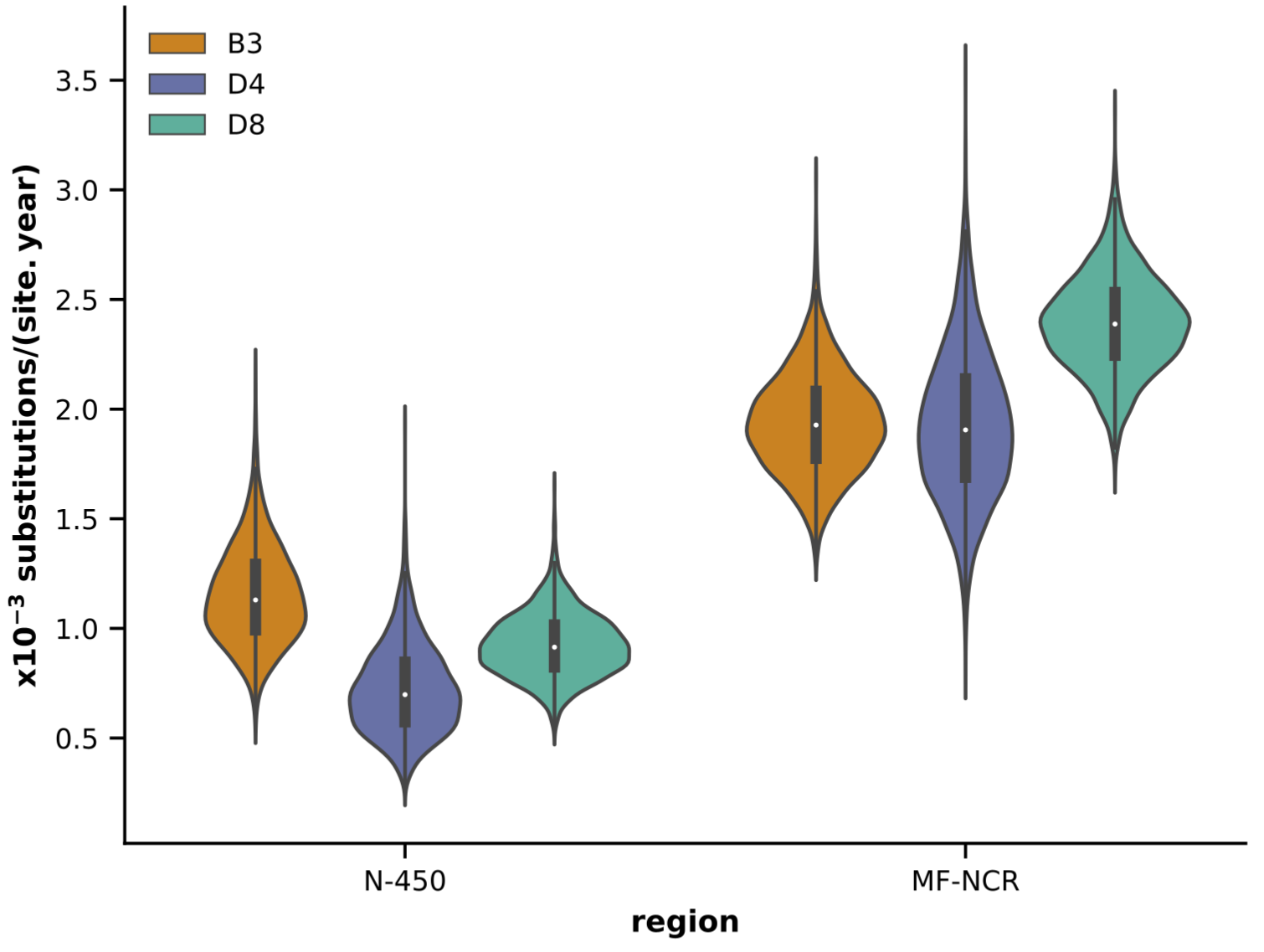


**Fig S6.5:** N-450 and MF-NCR genomic regions substitution rates of MeV genotypes B3, D4 and D8 estimated for verification datasets. Box-and-whisker plots show the minimum, maximum, quartiles and median of all rate estimates across the 3604 trees estimated in the logged BEAST chains (posterior). Violin plots show the distribution of each rate in the BEAST posterior for each dataset (see Table S6.1 for the 95% highest posterior density intervals).


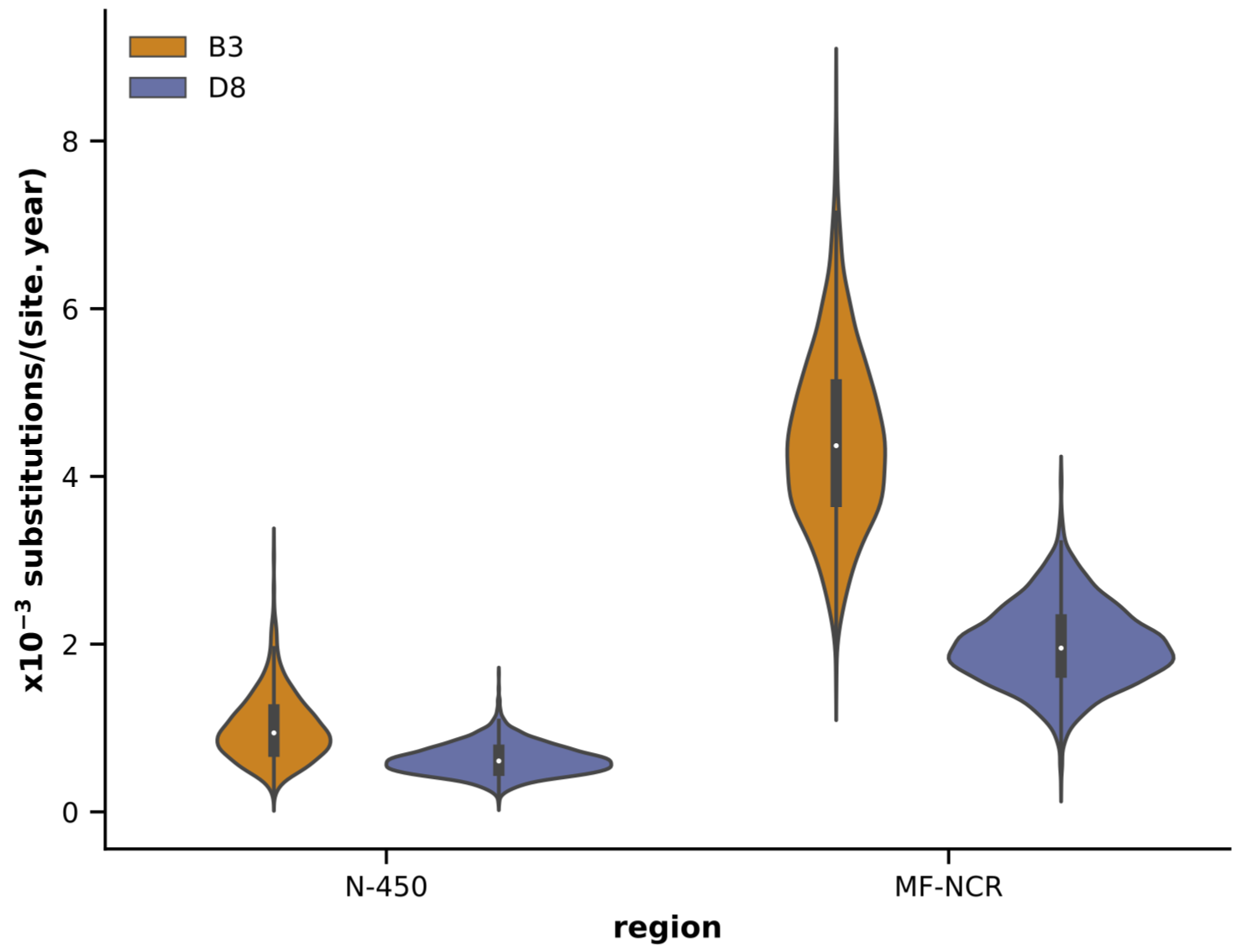


**Fig S6.6:** N-450 and MF-NCR genomic regions substitution rates of MeV genotypes B3 and D8 estimated for validation datasets. Refer to Fig S6.5’s legend for details.

# Model statistics


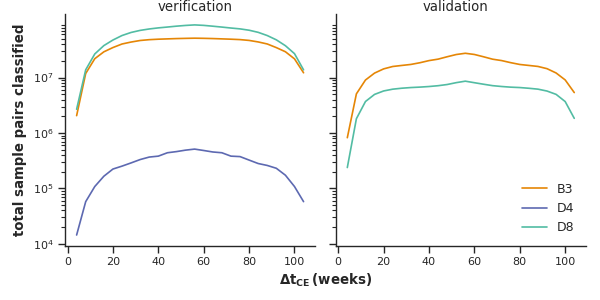


**Fig S6.7:** Number of sample pairs classified per Δt_CE_ for verification and validation datasets. This is equal to the number of combinations of sequences in pairs multiplied by the number of BEAST posterior trees. When the t_pCA_ is more recent than the older sample in the pair, the pair is not classified for the time point.

**Table S6.2:** Total number of validation dataset sample pairs classified as true negative, false negative, false positive or true positive for all cumulative evolutionary times.

|  |  | **Model** | |
| --- | --- | --- | --- |
|  |  | **R** | **U** |
| **BEAST** | **R** | 266,110,783 | 5,693,741 |
|  | **U** | 402,434,821 | 198,007,943 |

**Table S6.3:** True negative, false negative, false positive and true positive rates for the validation dataset for all cumulative evolutionary times.

|  |  | **Model** | | |
| --- | --- | --- | --- | --- |
|  |  | **R** | **U** |  |
| **BEAST** | **R** | 97.9 | 2.1 |  |
|  | **U** | 67.0 | 33.0 |  |

## Verification datasets

**
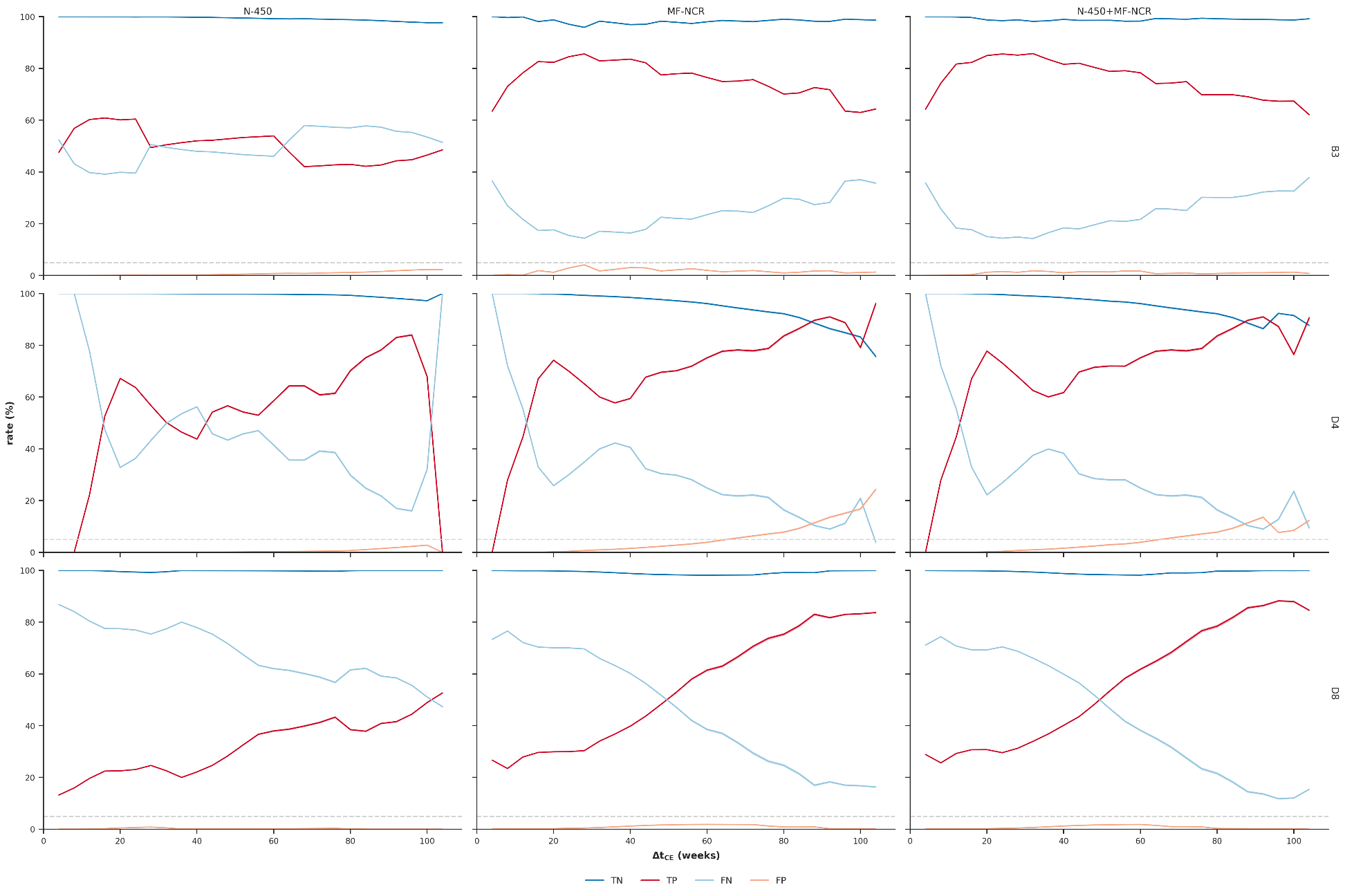
**

**Fig S6.8:** True negative (TN), false negative (FN), true positive (TP) and false positive (FP) rates for the verification set containing UK, Romania and GenBank N-450 and MF-NCR sequences used to calculate the substitution rate employed in the prediction of relatedness between samples. Lines represent mean rate and shaded areas the 95% HPD interval (very narrow intervals).


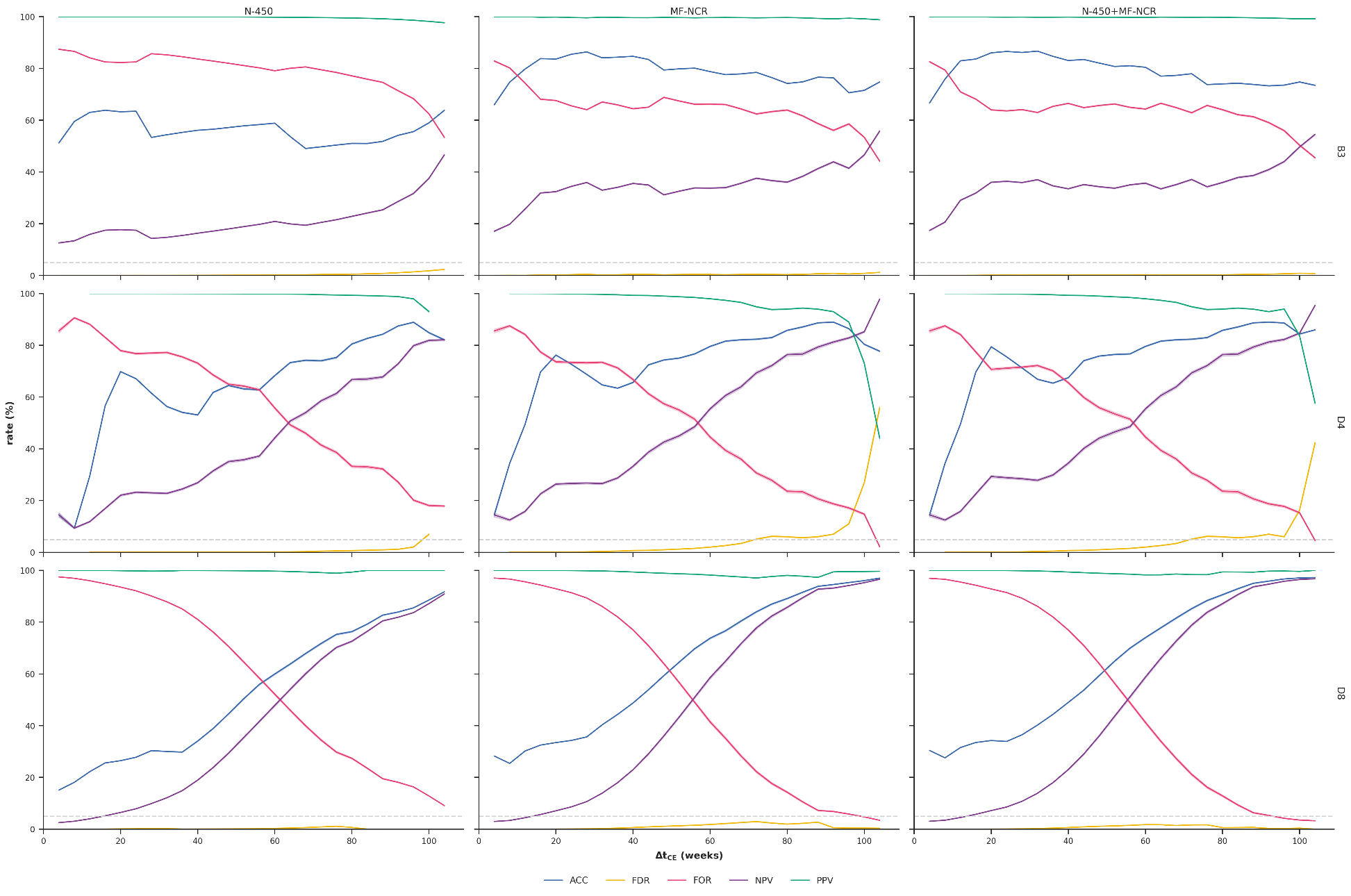


**Fig S6.9:** Accuracy (ACC), false detection rate (FDR), false omission rate (FOR), negative predictive value (NPV) and positive predictive value (PPV) rates for the verification set containing UK, Romania and GenBank N-450 and MF-NCR sequences used to calculate the substitution rate employed in the prediction of relatedness between samples. Lines represent mean rate and shaded areas the 95% HPD interval (very narrow intervals).

## Validation datasets

**
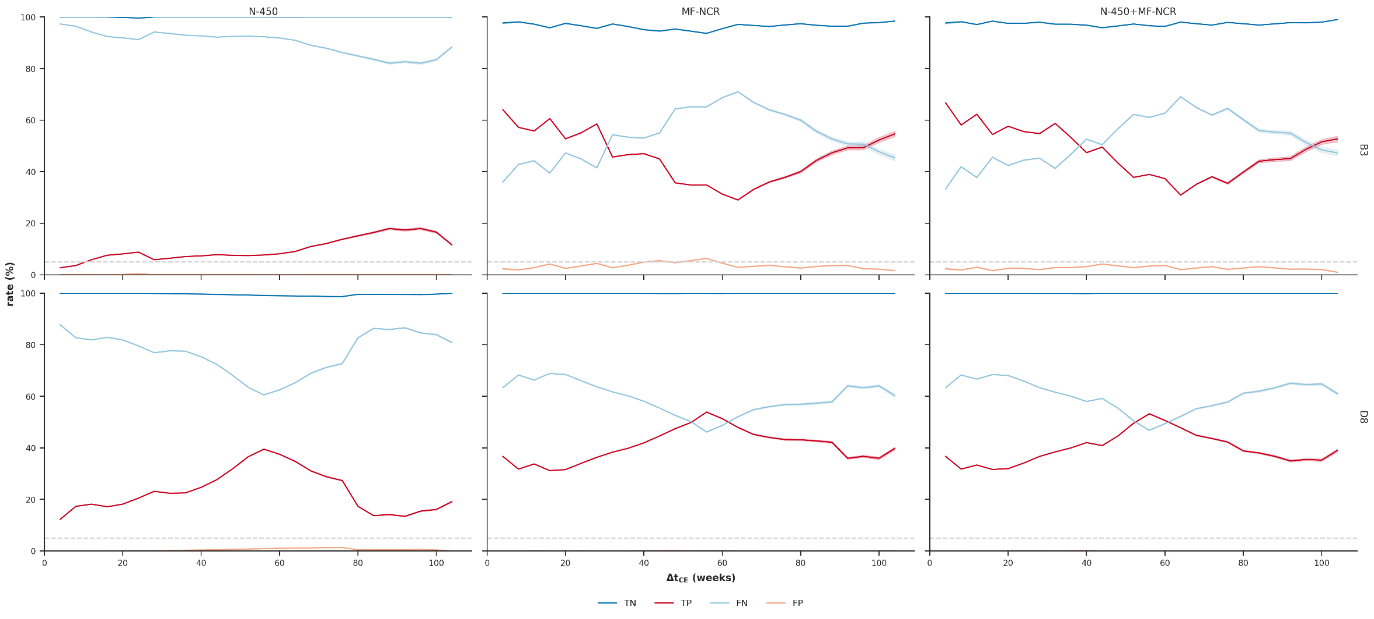
**

**Fig S6.10:** True negative (TN), false negative (FN), true positive (TP) and false positive (FP) rates for the validation set containing UK and GenBank N-450 and MF-NCR sequences. Lines represent mean rate and shaded areas the 95% HPD interval (very narrow intervals).

**
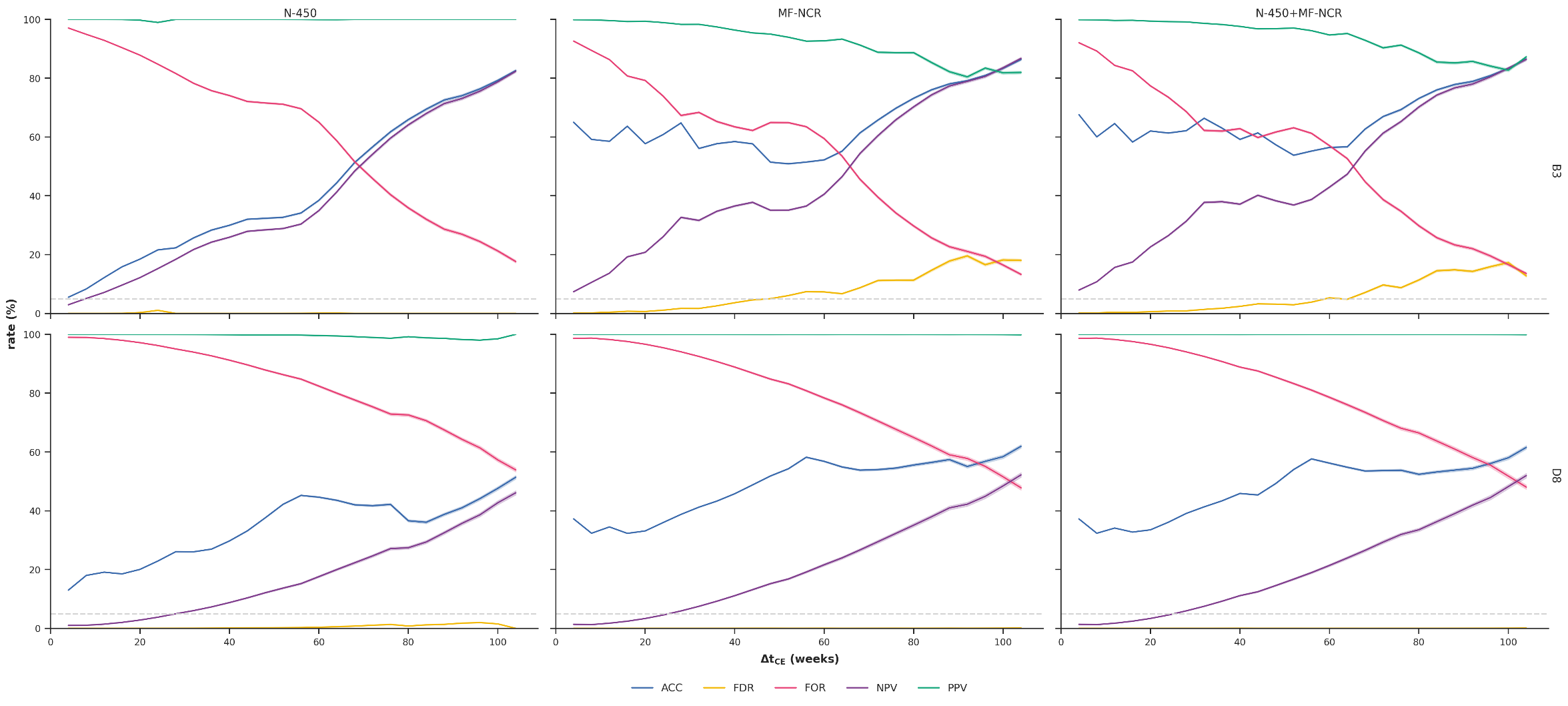
**

**Fig S6.11:** Accuracy (ACC), false detection rate (FDR), false omission rate (FOR), negative predictive value (NPV) and positive predictive value (PPV or precision) rates for the validation set containing UK and GenBank N-450 and MF-NCR sequences. Lines represent mean rate and shaded areas the 95% HPD interval (very narrow intervals).
