# Supplementary material for: Mind your Ps: A probabilistic model to aid the interpretation of molecular epidemiology data
Source: eBioMedicine. 2022 Apr 7;79:103989. doi: 10.1016/j.ebiom.2022.103989 (PMC9006250; doi:10.1016/j.ebiom.2022.103989)
Supplement: Supplementary file 7 [file mmc7.docx]

Supplement S1: Samples metadata.

Supplement S2: RT-PCR and PCR primers, mixes and conditions.

Supplement S3: Bioinformatics methods.

Supplement S4: Protocol and example application.

Supplement S5: Calculating expected substitutions spreadsheet.

Supplement S6: Supplementary figures and tables.
